# Calycindaphines A–J, *Daphniphyllum* alkaloids from the roots of *Daphniphyllum calycinum*†

**DOI:** 10.1039/d1ra00107h

**Published:** 2021-03-01

**Authors:** Ji Yang, Xin Liu, Jing Fu, Hao-Yuan Lyu, Li-Ping Bai, Zhi-Hong Jiang, Guo-Yuan Zhu

**Affiliations:** State Key Laboratory of Quality Research in Chinese Medicine, Guangdong-Hong Kong-Macao Joint Laboratory of Respiratory Infectious Disease, Macau Institute for Applied Research in Medicine and Health, Macau University of Science and Technology Macau People's Republic of China zhjiang@must.edu.mo gyzhu@must.edu.mo; Biology Institute, Qilu University of Technology (Shandong Academy of Sciences) Jinan 250103 China

## Abstract

Ten new *Daphniphyllum* alkaloids, calycindaphines A–J (1–10), together with seventeen known alkaloids were isolated from the roots of *Daphniphyllum calycinum*. Their structures were established by extensive spectroscopic methods and compared with data from literature. Compound 1 is a novel alkaloid with a new rearrangement C_22_ skeleton with the 5/8/7/5/5 ring system. Compound 2 represents the second example of calyciphylline G-type alkaloids. Compound 10 is the first example of secodaphniphylline-type alkaloid absent of the oxygen-bridge between C-25/C-29. The possible biogenetic pathways of 1 and 2 were also proposed. All the isolated compounds were evaluated for their bioactivities in three cell models. Compounds 22, 23, and 26 showed significant NF-κB transcriptional inhibitory activity at a concentration of 50 μM. Compounds 16 and 18 exhibited significant TGF-β inhibitory activity in HepG2 cells. Compounds 24 and 26 induced autophagic puncta and mediated the autophagic marker LC3-II conversion in HEK293 cells.

## Abstract 2

Ten new *Daphniphyllum* alkaloids, calycindaphines A–J (1–10), were isolated from *Daphniphyllum calycinum*. Compound 1 possesses a unique C_22_ skeleton with the 5/8/7/5/5 ring system and might be a key intermediate for daphhimalenine A formation.

## Graphical abstract

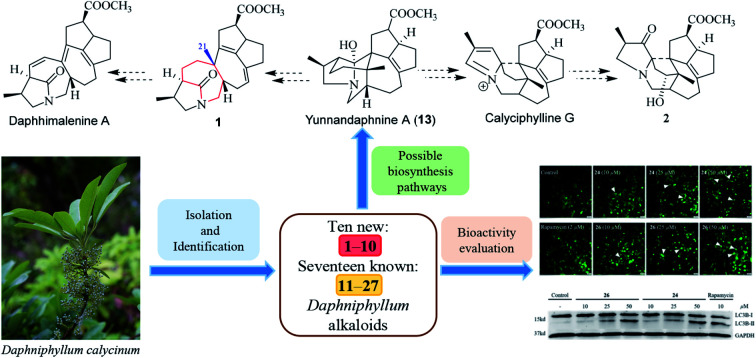

## Introduction

The *Daphniphyllum* alkaloids were characteristically distributed in plants of the *Daphniphyllum* family.^1–4^ To date, more than 200 *Daphniphyllum* alkaloids with nearly 20 skeletons have been isolated from ten species. *Daphniphyllum* alkaloids not only provided abundant structural types but also enlighten novel strategies for the total synthesis and biosynthesis of unusual skeletons.^5–10^*Daphniphyllum calycinum*, an evergreen tree native to southern China, provided an abundant resource of *Daphniphyllum* alkaloids.^11,12^ The leaves, stems, and roots of *D. calycinum* are used in Traditional Chinese Medicine to treat several symptoms including fever, asthma, inflammation, and influenza.^11–13^ Previous phytochemical investigations on the leaves and stems of *D. calycinum* led to the isolation of more than 50 *Daphniphyllum* alkaloids with 15 different skeletons. However, the chemical from the roots of the *D. calycinum* has not been reported. To seek potential bioactive alkaloids from *D. calycinum*, the roots of *D. calycinum* were first deeply studied. As a result, ten new *Daphniphyllum* alkaloids, named calycindaphines A–J (1–10), as well as 17 known alkaloids (11–27) were isolated from the roots of *D. calycinum*. In this paper, we reported the isolation, structural elucidation, and bioactivities of these isolates.

## Results and discussion

**Fig. 1:**
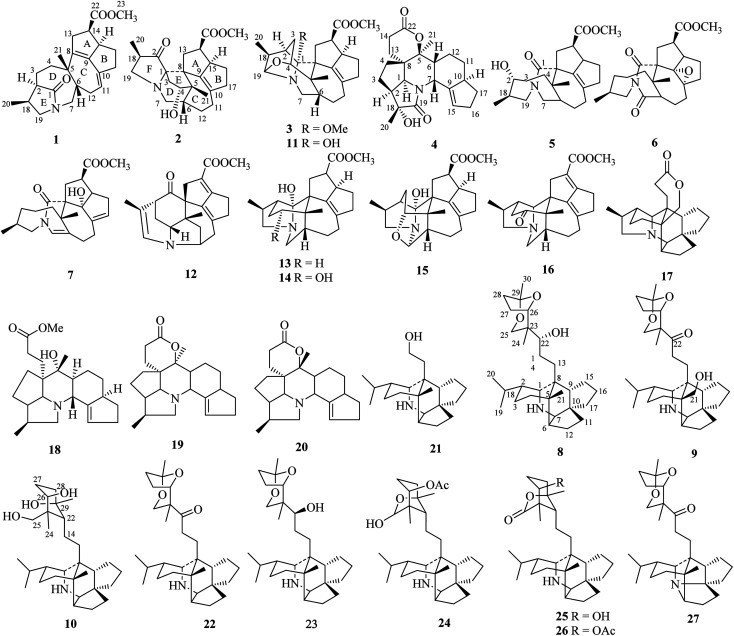
Chemical structures of compounds 1–27.

**Fig. 2:**
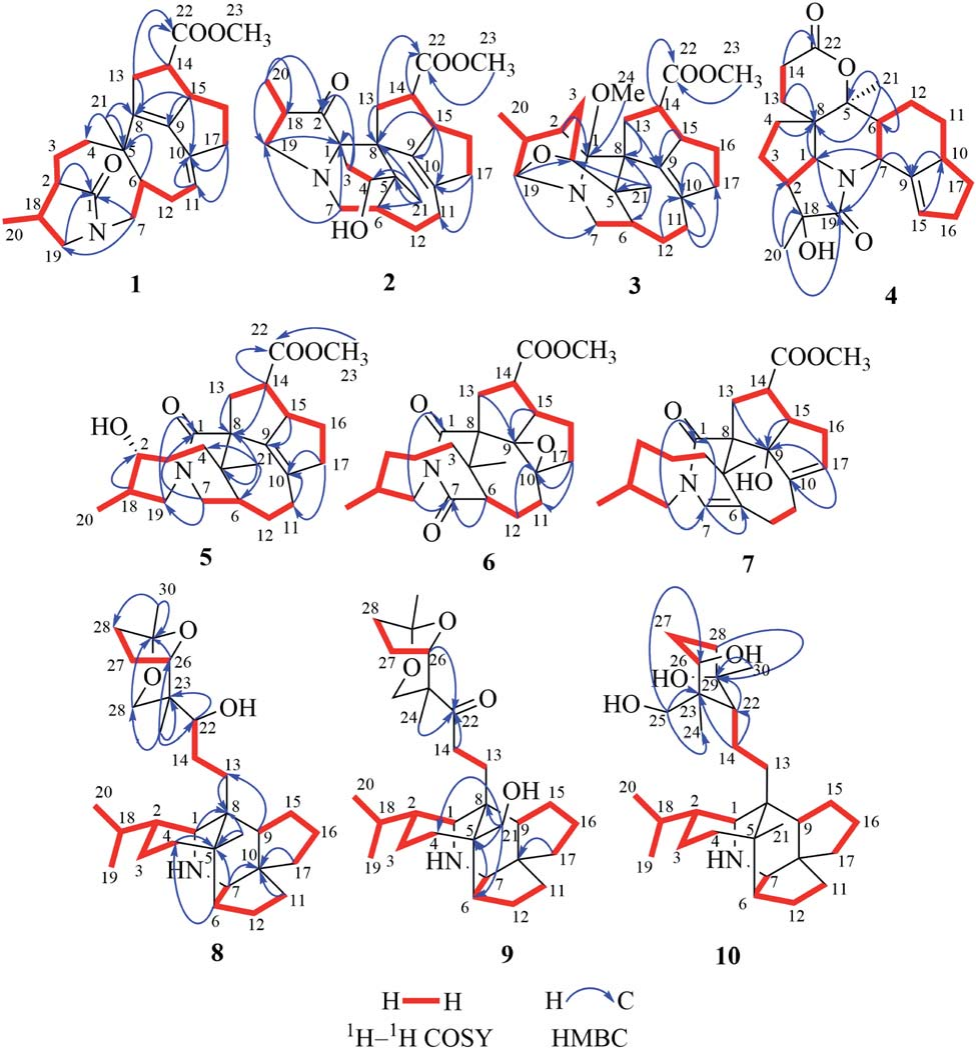
Key ^1^H–^1^H COSY and HMBC correlations of compounds 1–10.

**Fig. 3:**
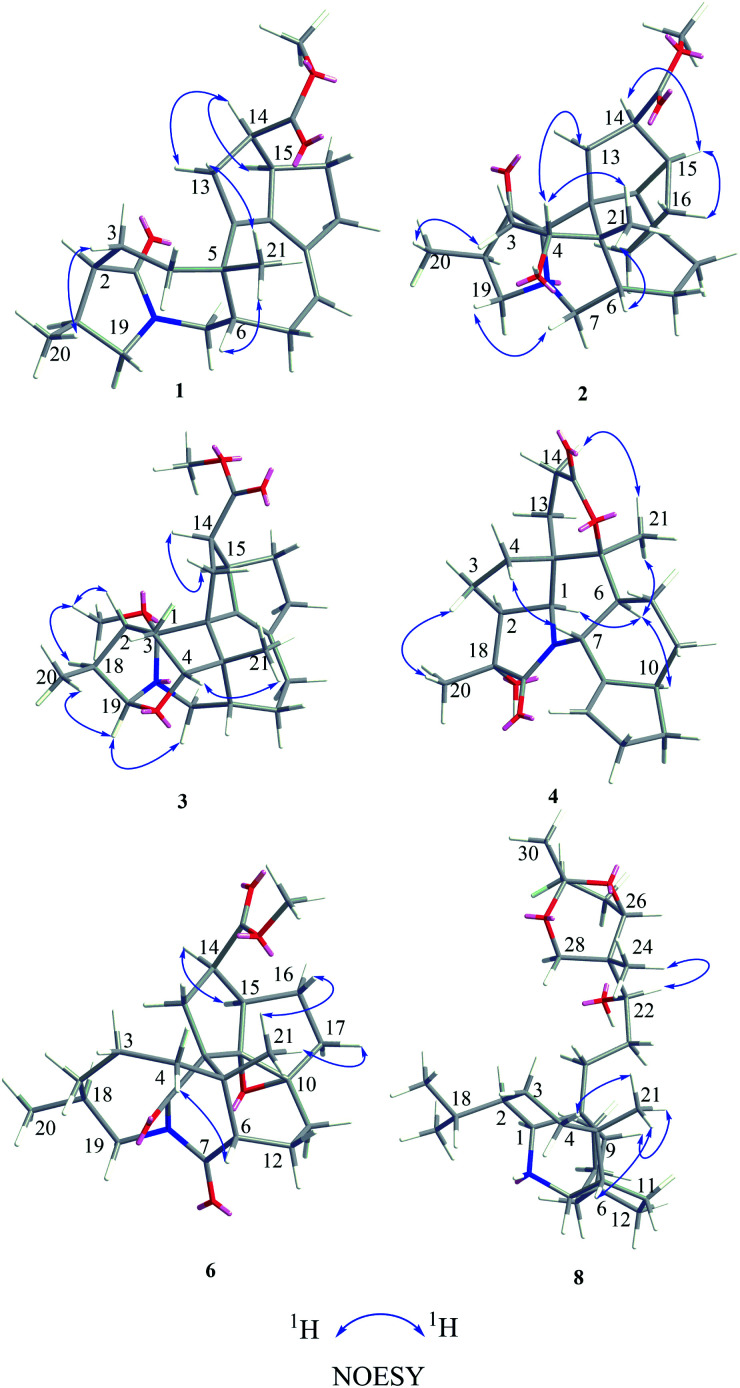
Key NOESY correlations of compounds 1–4, 6, and 8.

**Fig. 4:**
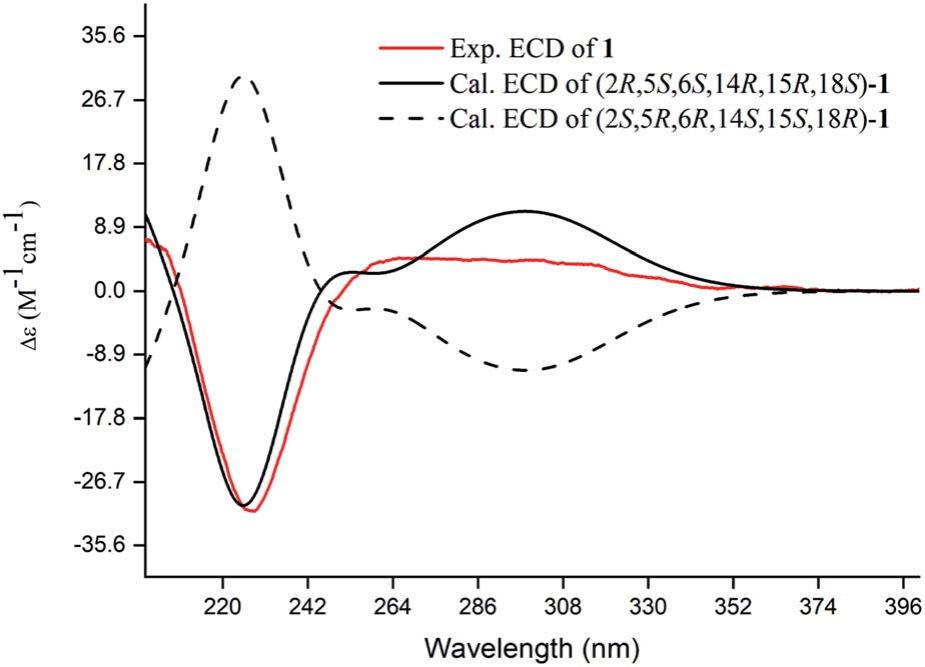
Experimental and calculated ECD spectra of compound 1.

Compound 1, a white amorphous powder, has a molecular formula of C_23_H_31_O_3_N as established by its HRESIMS data (*m*/*z* 370.2375 [M + H]^+^, calcd for 370.2377), with 9 degrees of unsaturation. IR absorptions implied the presence of an ester carbonyl (1733 cm^−1^) and a lactam (1644 cm^−1^).^14^ The ^1^H NMR data (Table 1) of 1 exhibited proton resonances of a methoxy (*δ*_H_ 3.61, s), a methyl singlet (*δ*_H_ 1.10, s), a methyl doublet (*δ*_H_ 1.15, d, *J* = 6.7 Hz), an olefinic proton (*δ*_H_ 5.42, d, *J* = 5.2 Hz). Its ^13^C NMR, DEPT, and HSQC spectra showed the occurrence of 23 carbon resonances (Table 2), which consisted of three methyls (a methoxy at *δ*_C_ 50.9), nine methylenes (two *N*-methylenes at *δ*_C_ 46.8 and 52.5), five methines (an olefinic methine at *δ*_C_ 118.9), six quaternary carbons (two carbonyls at *δ*_C_ 182.9 and 175.2, and three olefinic quaternary carbons at *δ*_C_ 140.6, 139.0, and 135.5). The above-mentioned spectroscopic data suggested that 1 possesses two carbonyls and two double bonds which accounting for four out of nine indices of hydrogen deficiency, and the remaining five degrees of unsaturation were speculated for the presence of a pentacyclic system in 1. The ^1^H–^1^H COSY spectrum of 1 suggested three proton-bearing structural moieties: C-4/C-3/C-2/C-18/C-19/20, C-7/C-1/C-12/C-11, and C-13/C-14/C-15/C-16/C-17 (Fig. 2). These three fragments, quaternary carbons, and the nitrogen atom were then connected by the detailed HMBC analysis (Fig. 2). The HMBC correlations from H-13/H-14 to C-8/C-9/C-22, H-16 to C-9/C-10, H-17 to C-9/C-10/C-11, H-11 to C-10/C-17, and H-23 to C-22, led to the assignment of rings A and B. Moreover, HMBC cross-peaks from H-21 to C-4/C-5/C-6/C-8 and H-6 to C-4/C-5/C-8 indicated the presence of C-5/C-6/C-8 linkage which form a seven-membered ring (ring C). HMBC correlations from H-2/H-7/H-19 to C-1, H-3/H-4/H-6 to C-5, H-7 to C-5/C-19, and H-19 to C-7 established the connectivity of rings D and E. Thus, the planar structure of 1 was assigned as shown in Fig. 2. Compound 1 has a fused 5/8/7/5/5 ring system, which contains a 1-azabicyclo[5.2.1]decane ring (rings D/E) with a ketone at C-1 and two methyls at C-18 and C-2, a cyclohepta-1,3-diene ring (ring C) with two double bonds between C-8/C-9 and C-10/C-11, and a bicyclo[3.3.0]octane ring (rings A/B) with a methoxy carbonyl at C-14 as shown in Fig. 2. Comparing with the literature, the ring system of 1 was the same as those of daphhimalenine A.^14^ However, compound 1 possesses C_22_ carbon skeleton like most of *Daphniphyllum* alkaloids, while daphhimalenine A loses C-21 methyl to form a C_21_ carbon skeleton. The ^3^*J*_H-13a/H-14_ = 7.5 Hz suggest that H-15/H-14 of 1 was α-orientation and same as that of daphhimalenine A.^14^ Based on the same biosynthetic origin as *Daphniphyllum* alkaloids, the configuration of H-6 was identified as β-orientation.^11,13^ The relative configurations of C-20, C-21, and C-2–C-3 bond was dissented as β-orientation by the key NOESY correlations (Fig. 3) from H-21 to H-6/H-13b and H-20 to H-3 as well as the comparison with the literature.^14^ To determine the absolute configuration of 1, the calculated ECD were performed using the time-dependent density functional theory at the PBE0-D3(BJ)/def2-SVP level for (2*R*,5*S*,6*S*,14*R*,15*R*,18*S*)-1 and (2*S*,5*R*,6*R*,14*S*,15*S*,18*R*)-1. The ECD curve of 1 showed a negative Cotton effect at 217 (−51.89) nm, which was consistent with the calculated ECD spectrum of (2*R*,5*S*,6*S*,14*R*,15*R*,18*S*)-1 (Fig. 4). Accordingly, the absolute configuration of 1 was determined as 2*R*,5*S*,6*S*,14*R*,15*R*,18*S*. The structure of 1 was thereby established (Fig. 1) and named calycindaphine A.

**Table 1:** ^1^H NMR (600 MHz) spectroscopic data of compounds 1–10 in CDCl_3_

No.	1	2	3	4	5	6	7	8	9	10
1				3.80 (d, 4.2)				3.03 (s)	2.99 (s)	3.11 (s)
2a	2.24 (m)		2.15 (m)	2.65 (m)	3.59 (m)	1.40 (m)	1.69 (m)	1.03 (m)	0.96 (m)	1.09 (m)
2b						0.84 (m)	1.32 (m)			
3a	2.51 (m)	2.00 (m)	2.03 (m)	2.06 (m)	1.60 (m)	1.70 (m)	1.50 (m)	1.89 (m)	1.55 (m)	1.50 (m)
3b	1.94 (m)	1.66 (dd, 14.4, 4.4)	1.74 (m)	1.66 (m)	2.00 (m)	1.42 (m)		1.40 (m)		1.29 (m)
4a	2.02 (m)	4.07 (dd, 10.5, 4.4)	3.22 (t, 3.1)	2.00 (m)	1.61 (m)	1.83 (m)	1.81 (m)	1.17 (m)	1.67 (m)	1.59 (m)
4b	1.63 (m)			1.42 (m)	1.76 (m)	1.35 (m)	1.38 (m)		1.60 (m)	1.15 (m)
6	2.33 (m)	2.01 (m)	2.55 (m)	2.79 (m)	2.38 (m)			1.91 (t, 5.3)	1.99 (t, 5.3)	1.91 (t, 5.1)
7a	4.32 (t, 13.1)	2.88 (dd, 14.2, 8.9)	3.40 (dd, 14.3, 9.8)	4.98 (m)	3.36 (dd, 13.6, 10.1)	2.78 (m)	5.97 (s)	2.30 (d, 5.9)	2.60 (t, 4.6)	2.56 (d, 3.5)
7b	2.48 (m)		3.14 (m)		3.73 (dd, 13.6, 8.2)					
9								1.72 (m)	1.75 (m)	1.03 (t, 3.4)
10				2.54 (m)						
11a	5.32 (d, 5.2)	2.42 (m)	2.17 (m)	1.83 (m)	2.24 (m)	1.97 (m)	2.55 (dd, 12.9, 5.8)	1.65 (m)	1.68 (m)	1.67 (m)
11b		1.99 (m)		1.29 (m)		1.74 (m)	2.21 (m)	1.48 (m)	1.55 (m)	1.49 (m)
12a	2.67 (m)	2.01 (m)	1.90 (m)	2.07 (m)	1.60 (m)	2.31 (m)	2.38 (m)	1.59 (m)	1.79 (m)	1.59 (m)
12b	1.97 (m)	1.61 (m)	1.61 (m)	1.87 (m)	2.24 (m)	1.97 (m)		1.40 (m)	1.61 (m)	1.41 (m)
13a	3.45 (m)	2.79 (m)	2.53 (m)	1.84 (m)	2.31 (m)	2.65 (dd, 13.8, 7.9)	2.27 (m)	2.04 (m)	1.65 (m)	1.69 (m)
13b	2.89 (d, 15.5)	2.35 (m)	2.28 (dd, 13.5, 8.8)	1.68 (m)	2.61 (m)	2.30 (m)	2.07 (m)	1.96 (m)		1.54 (m)
14a	3.18 (t, 7.6)	2.93 (m)	3.18 (m)	2.74 (m)	3.27 (dt, 11.3, 8.1)	3.41 (dt, 12.2, 7.0)	3.61 (m)	2.92 (m)	2.86 (m)	1.30 (m)
14b								1.28 (m)	2.65 (m)	1.41 (m)
15a	3.63 (m)	2.93 (m)	3.62 (m)	5.48 (m)	3.38 (m)	2.78 (m)	2.75 (q, 8.0)	1.78 (m)	1.69 (m)	1.67 (m)
15b								1.72 (m)	1.61 (m)	1.78 (m)
16a	1.80 (m)	1.79 (m)	1.83 (m)	2.18 (m)	1.22 (m)	1.78 (m)	2.29 (m)	1.73 (m)	1.73 (m)	1.75 (m)
16b	1.00 (m)	1.17 (m)	1.17 (m)		1.88 (dt, 12.1, 6.8)	1.48 (m)	2.05 (m)	1.44 (m)	1.46 (m)	1.45 (m)
17a	2.68 (m)	2.53 (m)	2.49 (m)	2.09 (m)	2.21 (m)	2.35 (m)	5.54 (m)	1.66 (m)	1.69 (m)	1.47 (m)
17b	2.46 (m)	2.23 (m)	2.21 (m)	1.42 (m)	2.46 (m)	1.90 (m)		1.54 (m)	1.56 (m)	1.16 (m)
18	2.49 (m)	2.28 (m)	2.35 (t, 6.3)		2.24 (m)	2.15 (m)	2.15 (m)	1.49 (m)	1.47 (m)	1.51 (m)
19a	3.32 (d, 9.4)	3.22 (m)	4.42 (s)		2.29 (dd, 13.6, 8.6)	4.04 (dd, 13.1, 7.9)	4.37 (dd, 13.2, 5.6)	0.90 (d, 6.5)	0.91 (d, 6.5)	0.94 (d, 6.5)
19b	3.27 (d, 9.4)	2.02 (m)			4.46 (dd, 13.6, 7.7)	3.89 (dd, 13.1, 8.9)	2.31 (m)			
20	1.15 (d, 6.7)	1.02 (d, 7.1)	1.13 (d, 6.7)	1.49 (s)	0.99 (d, 6.7)	0.93 (d, 6.9)	0.87 (d, 6.8)	0.89 (d, 6.5)	0.90 (d, 6.5)	0.89 (m)
21a	1.10 (s)	1.22 (s)	0.97 (s)	1.47 (s)	1.16 (s)	1.22 (s)	1.08 (s)	0.78 (s)	3.72 (d, 10.6)	0.76 (s)
21b									3.49 (d, 10.6)	
22								3.94 (m)		1.68 (m)
23	3.61 (s)	3.61 (s)	3.62 (s)		3.65 (s)	3.70 (s)	3.68 (s)			
24			3.25 (s)					0.59 (s)	0.78 (s)	0.89 (s)
25a								3.58 (dd, 11.8, 1.5)	4.25 (d, 12.1)	3.65 (m)
25b								3.51 (d, 11.8)	3.52 (d, 12.1)	3.49 (d, 10.3)
26								4.49 (d, 6.0)	4.66 (d, 6.9)	3.74 (dd, 11.2, 4.0)
27a								2.07 (m)	2.07 (m)	1.74 (m)
27b								1.96 (m)	1.03 (m)	1.50 (m)
28a								2.05 (m)	2.07 (m)	1.76 (m)
28b								1.83 (m)	1.87 (m)	1.47 (m)
30								1.47 (s)	1.43 (s)	1.20 (s)

**Table 2:** ^13^C NMR (150 MHz) spectroscopic data of compounds 1–10 in CDCl_3_

No.	1	2	3	4	5	6	7	8	9	10
1	182.9	73.6	98.7	63.6	174.4	175.7	175.6	48.2	48.6	47.7
2	46.3	220.8	45.5	47.8	72.9	32.3	32.3	42.7	42.6	42.6
3	30.2	33.3	23.5	22.9	37.1	26.2	23.0	20.8	20.0	20.7
4	41.6	79.1	82.8	27.3	42.7	48.3	40.0	39.2	32.3	39.1
5	42.9	51.0	45.9	84.2	57.1	41.9	40.7	36.9	41.7	36.7
6	46.5	36.0	39.1	33.9	40.0	53.5	130.6	47.8	45.7	47.6
7	46.8	44.5	44.7	47.1	50.9	176.9	126.9	60.1	59.7	59.8
8	140.6	60.6	34.1	49.0	41.8	62.9	65.5	36.9	45.7	37.2
9	135.5	143.1	143.2	143.0	139.1	96.0	97.2	53.9	52.8	54.2
10	139.0	136.4	135.0	44.7	133.5	84.7	145.9	50.5	50.5	50.6
11	118.9	26.3	25.9	25.4	24.5	32.5	31.3	39.9	39.4	40.0
12	33.7	27.0	22.9	21.2	25.5	24.1	27.8	22.9	23.2	23.0
13	42.1	33.7	38.2	23.1	34.8	31.5	31.1	25.0	25.8	36.2
14	46.7	55.1	42.8	26.4	42.0	43.2	43.7	30.1	34.1	20.7
15	54.2	43.0	54.5	124.8	52.9	54.0	56.8	30.8	29.7	30.6
16	28.4	28.6	28.5	31.0	28.5	20.2	31.4	26.8	26.7	26.9
17	38.9	42.5	42.6	34.5	42.1	34.8	130.9	36.3	36.2	36.3
18	28.1	43.8	36.3	77.3	38.4	33.8	28.8	28.8	28.7	28.8
19	52.5	54.2	96.5	174.4	52.4	45.1	51.9	21.2	21.0	21.0
20	13.3	11.8	10.8	20.5	18.1	26.1	20.4	21.1	21.0	21.0
21	33.3	19.1	20.6	19.7	25.0	21.4	22.9	21.3	66.2	21.1
22	175.2	176.3	176.5	170.8	176.1	174.1	174.4	75.5	213.0	52.2
23	50.9	51.0	51.1		51.3	51.7	51.5	39.2	49.8	43.8
24			50.4					14.8	17.7	10.7
25								67.6	65.4	72.0
26								80.0	81.0	76.7
27								25.0	24.6	28.7
28								33.6	33.8	40.7
29								105.1	105.3	73.9
30								23.8	23.7	23.9

**Scheme 1:**
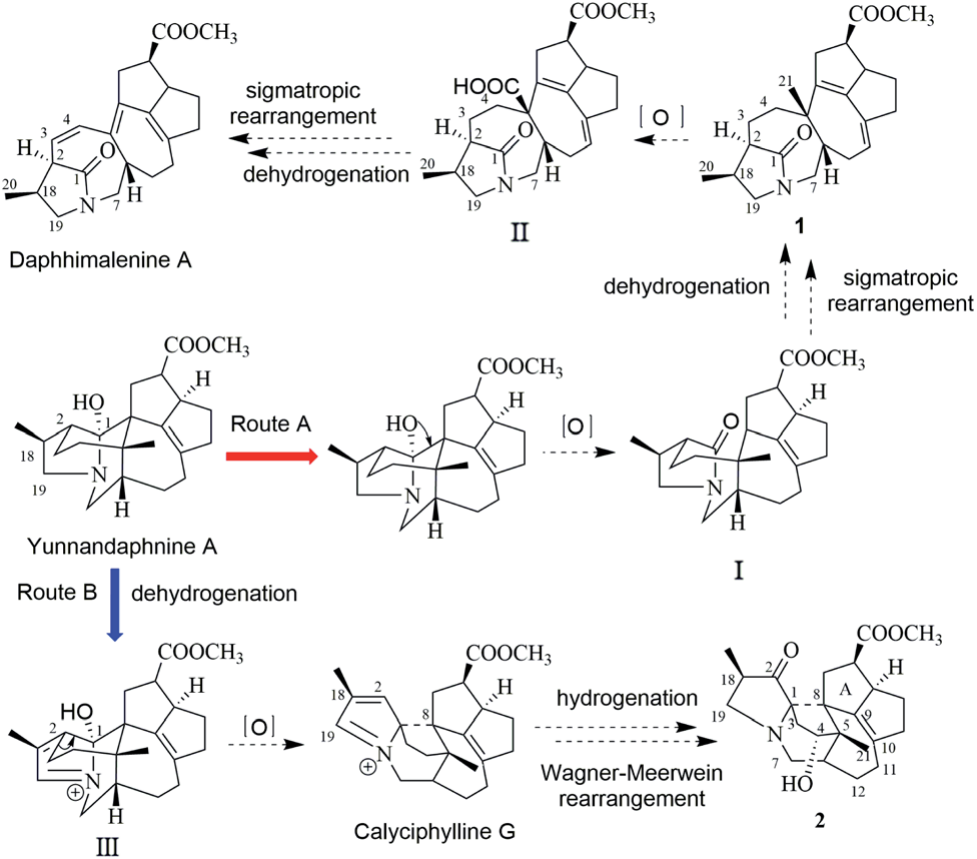
Biogenetic pathway proposed for compounds 1 and 2, daphhimalenine A, and calyciphylline G.

In the early report, the possible biogenetic pathway of daphhimalenine A was proposed that its precursor daphhimalenine B underwent multi-step oxidation to lose the C-21 and rearranged to form 1-azabicyclo[5.2.1]decane ring system.^14^ Calycindaphine A (1) has the same ring system with daphhimalenine A but reserving the key C-21 methyl. According to the structure of 1, another possible biogenetic pathway to form 1-azabicyclo[5.2.1]decane ring system was proposed, and shown in Scheme 1. The biogenetic origin of 1 and daphhimalenine A seems to be modified from a yuzurimine-type alkaloid, yunnandaphnine A (13).^7^ Yunnandaphnine A might undergo the oxidation of C-1 and the breakdown of C-1/C-8 bond to form 1-azabicyclo[5.2.1]decane ring. Then, the intermediate I should undergo the dehydrogenation and sigmatropic rearrangement procedures to yield 1 (C_22_ skeleton). The C-21 methyl of 1 should further be oxidated to give II. Then the oxidative decarboxylation and sigmatropic rearrangement of II afford daphhimalenine A (C_21_ skeleton).

Compound 2 was isolated as a white amorphous powder. The molecular formula of 2 was assigned as C_23_H_31_O_4_N based on the quasi-molecular ion peak at *m*/*z* 386.2324 [M + H]^+^ in its HRESIMS spectrum, requiring nine indices of hydrogen deficiency. The ^1^H NMR spectrum (Table 1) of 2 revealed a methoxy (*δ*_H_ 3.61, s), a methyl singlet (*δ*_H_ 1.22, s), a methine doublet (*δ*_H_ 1.02, d, *J* = 7.1 Hz), an oxidized methine (*δ*_H_ 4.07, dd, *J* = 10.5, 4.4 Hz). The ^13^C, DEPT, and HSQC spectra of 2 described 23 carbon resonances constituting with three methyls (a methoxy at *δ*_C_ 51.0), eight methylenes (two *N*-methylenes at *δ*_C_ 54.3 and 44.5), five methines (an oxidized methine at *δ*_C_ 79.2) and seven quaternary carbons (a carbonyl at *δ*_C_ 220.8, an ester carbonyl at *δ*_C_ 176.3, a couple of double bond carbons at *δ*_C_ 143.1 and 136.4, and an *O*/*N*-quaternary carbon at *δ*_C_ 73.6), shown in Table 2. Comparing with the known *Daphniphyllum* alkaloids, the spectroscopic data of 2 is similar to those of calyciphylline G^15^ except for the absence of *Δ*_2,18_ and *Δ*_19,*N*_ and the presence of additional carbonyl and hydroxyl groups at C-2 and C-4 in 2, respectively. These function groups were assigned by the HMBC correlations from H-3/H-18/H-19/H-20 to C-2 and H-3/H-6/H-21 to C-4 as well as the chemical shift of C-4 (Fig. 2). The relative stereochemistry of 2 was elucidated by the NOESY experiment. The correlations of H-4/H-21/H-13b and H-21/H-6 suggested that they are on the same side and β-oriented (Fig. 3), which is the same as those of calyciphylline G.^15^ Accordingly, the hydroxyl group at C-4 was placed at α-orientation. Thus, the structure of 2 was determined as shown in Fig. 1 and named calycindaphine B.

The calyciphylline G was isolated as a quaternary amine alkaloid containing a 5-azatricyclo[6.2.1.01,5]undecane ring in 2007.^15^ However, the possible biogenetic pathway of this unprecedented fused-hexacyclic skeleton has not been described. Comparison of the structural features of calyciphylline G and 2 suggested that the calyciphylline G might be regarded as the key intermediate for 2. A plausible biogenetic pathway for this fused-hexacyclic skeleton is proposed as shown in Scheme 1. Calycindaphine B (2) and calyciphylline G might also be generated from yunnandaphnine A,^7^ which might be dehydrated in ring E to form intermediated III. Then, the intermediated III should be reduced and undergo the Wagner–Meerwein rearrangement to yield calyciphylline G. Following, hydrogenation of *Δ*_2,18_/*Δ*_19,*N*_ and oxidation of C-2 and C-4 result in the formation of 2.

Compound 3 has a molecular formula of C_24_H_33_O_4_N with nine degrees of unsaturation. Analysis of spectroscopic data of 3 suggested that 3 have the same skeleton as that of calyciphylline E (11).^16^ The major difference is the presence of an additional methoxy group in 3. Based on the HMBC correlation from *H*-methoxy (*δ*_H_ 3.25, s) to C-1 (*δ*_C_ 98.3), the methoxy group was placed at C-1 (Fig. 2). The NOESY correlations (Fig. 3) between *H*-methoxy to H-2/H-18 suggest that they are on the same side and assigned as an α-orientation. Consequently, the structure of 3 was identified as shown in Fig. 1, and named calycindaphine C.

The molecular formula of compound 4 was deduced as C_22_H_29_O_4_N on the basis of its HRESIMS data. The NMR spectroscopic data of 4 were closely related to that of oldhamiphylline A^17^ except that the hydroxylated methine at *δ*_C_ 75.8, the methylene at *δ*_C_ 37.1, and the *N*-substituted methylene at *δ*_C_ 61.34 in oldhamiphylline A are replaced by a methylene at *δ*_C_ 25.4 (C-11), a hydroxylated quaternary carbon at *δ*_C_ 77.4 (C-18), and a lactam carbonyl carbon at *δ*_C_ 174.4 (C-19) in 4, respectively. These changes were proved by the HMBC correlations from H-10/H-12 to C-11, H-1/H-2/H-3/H-20 to C-18, and H-1/H-7/H-20 to C-19 (Fig. 2). The significant NOESY cross-peak of H-21/H-1 demonstrated the H-21 and H-1 were cofacial and placed C-21 to α-orientation (Fig. 3). Furthermore, the NOESY correlation from H-20 to H-3 indicated there are on the same side and placed C-20 to β-orientation. Accordingly, the hydroxyl group attaching to C-18 was assigned as β-orientation. Therefore, the structure of 4 was identified as shown in Fig. 1, and named calycindaphine D.

Calycindaphines E–G (5–7) possess the molecular formula C_23_H_33_O_4_N, C_23_H_31_O_5_N, and C_23_H_31_O_4_N, respectively. Their NMR data analysis suggested that compounds 5–7 belong to daphnezomine F-type skeleton.^18^ The NMR data of 5 are similar to those of daphlongeranine C^18^ except that the hydroxy methylene at C-21 in daphlongeranine C was replaced by a singlet methyl in 5. This change was supported by the HMBC correlations from H-21 to C-4/C-5/C-6/C-8 and H-4/H-6 to C-5/C-21. Compound 6 is structurally like 5 except that the C-9/C-10 double bond, the methylene at C-7, and the *O*-methine at C-2 in 5 are absented in 6, and an acylamide, two oxygenated quaternary carbons, and a methylene were presented in 6, respectively. The oxygenated C-9/C-10 in 6 were devised as an epoxy three-membered ring by the chemical shifts of C-9 and C-10 combined with the exclusive molecular formula from the exact result of HRESIMS. In addition, the HMBC correlations from H-6/H-12/H-19 to the extra acylamide (C-7), H-15/H-16/H-17 to the pair of oxygenated quaternary carbons (C-9/C-10), and H-20/H-18/H-4/H-3 to C-2 supported the above conjectures. Comparison of the chemical shifts of C-9 and C-10 with those of alkaloids containing epoxy group at C-9 and C-10 suggested that the epoxy group in 6 is α-oriented.^19–21^ Careful analysis of NMR data of 7 indicated that 7 is a daphnezomine F-type alkaloid with two double bonds and a hydroxylated quaternary carbon. HMBC correlations from H-7 to C-1/C-5/C-6/C-12/C-19, H-4/H-12/H-21 to C-6, H-15/H-16/H-11 to C-10/C-17, and H-14/H-15 to C-9 implied that two double bonds were placed at C-7/C-6 and C-10/C-17, and the hydroxylated quaternary carbon was fixed at C-9. Thus, the structures of 5–7 were determined as shown (Fig. 1).

The 1D NMR data of 8 suggested that compound 8 was closely related to 23.^11^ The major differences between 8 and 23 were that the chemical shift of H-22 was down-shielded from *δ*_H_ 3.33 (in 23) to *δ*_H_ 3.94 (in 8), which might be caused by the different configuration of C-22. Furthermore, analysis of the ^1^H–^1^H COSY and HMBC spectra implied that 8 and 23 have same planar structure. The NOESY cross-peak (Fig. 3) between H-22/H-24 in 8 illustrating that these protons are in cofacial and assigned to be β-orientation, which is opposite to that of 23. Furthermore, the optical value of 8 and 23 was measured as [*α*]^22.5^_D_ +26.8 (*c* = 0.5, MeOH) and [*α*]^22.5^_D_ −49.4 (*c* = 0.5, MeOH) respectively, which also provided evidence for the different configuration of C-22 in 8 and 23. Accordingly, compound 8 was elucidated as shown in Fig. 1 and named calycindaphine H.

Calycindaphine I (9) has a molecular formula C_30_H_47_O_4_N. Comparison of its 1D NMR spectra with those of 8 showed that the hydroxylated methine at *δ*_C_ 75.5 (C-22) and methyl at *δ*_C_ 21.3 (C-21) in 8 are replaced by a carbonyl at *δ*_C_ 213.0 (C-22) and a hydroxylated methylene carbon at *δ*_C_ 66.2 (C-21) in 9, respectively. These changes were further confirmed by the HMBC correlations from H-21 to C-4/C-5/C-6/C-8 and H-13/H-14/H-24 to C-22 (Fig. 2). Thus, the structure of 9 was determined as shown in Fig. 1.

Compound 10 showed a protonated [M + H]^+^ molecular ion at *m*/*z* 474.3958, corresponding to a molecular formula of C_30_H_51_O_3_N, with six indices of hydrogen deficiency. The 1D NMR data of 10 are similar to those of daphnioldhanine F,^22^ except that the characteristic hemiacetal carbon at C-25 in daphnioldhanine F was replaced by an oxidized methylene [*δ*_C_ 72.0, and *δ*_H_ 3.65 (m), 3.49 (d, *J* = 10.3 Hz)] in 10. The HMBC correlations (Fig. 3) from H-25 to C-22/C-23/C-24 and H-24 to C-22/C-23/C-25/C-26 confirmed the assignment. The data of HRESIMS suggested that 10 has one less degree of unsaturation and a more H_2_O unit than that of the daphnioldhanine F. The chemical shift of C-29 in 10 appears in upfield shift (*ca.* 11 ppm) than the similar alkaloids possessing the linkage C-25–O–C-29,^22,23^ which supported that the specific linkage of C-25–O–C-29 in secodaphniphylline-type alkaloids is broken to form a hydroxy at C-25 and C-29 in 10, respectively. The above spectroscopic evidence deduced the structure of 10 as depicted in Fig. 1, and named calycindaphine J.

By NMR data analysis and comparison of the reported spectroscopic data, 17 known compounds (11–27) were identified as calyciphylline E (11),^16^ calyciphylline Q (12),^24^ yunnandaphnine A (13),^7^ macrodaphniphyllamine (14),^7^ yunnandaphnine E (15),^7^ caldaphnidine A (16),^11^ (−)-bukittinggine (17),^25^ longistylumphylline C (18),^26^ deoxyisocalyciphylline B (19),^27^ deoxycalyciphylline B (20),^25,27^ caldaphnidine D (21),^11^ secodaphniphylline (22),^28^ caldaphnidine E (23),^11^ daphnioldhanin G (24),^22^ daphnioldhanin D (25),^29^ daphnioldhanin E (26),^22^ and calyciphylline D (27).^30^

**Fig. 5:**
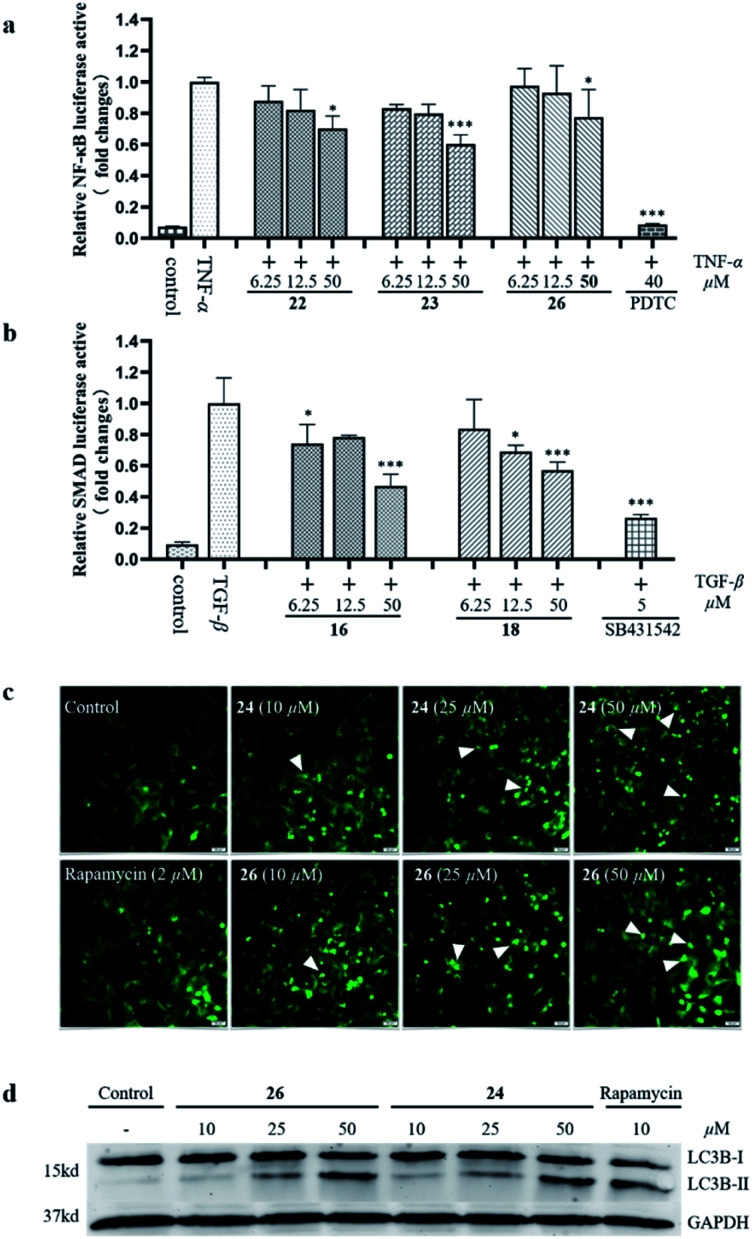
The effects of isolated alkaloids on TNFα-induced NF-κB activation (a), TGF-β/SMAD pathway (b), and cell autophagy (c and d).

Previous phytochemical studies have shown that *Daphniphyllum* alkaloids from *D. calycinum* mainly focused on the anti-cancer effect.^15,31^ But, in the traditional folk medicine, the plants of *D. calycinum* are extensively used to treat different diseases which are closely related to inflammation.^11–13^ To provide more evidences for the pharmacological action of alkaloids from *D. calycinum*, all isolated alkaloids were evaluated for their effects on TNFα-induced NF-κB activation, TGF-β pathway, and cell autophagy. The bioassay results showed that compounds 22, 23, and 26 inhibited TNFα-induced NF-κB activation in a dose dependent manner (Fig. 5a). Compounds 16 and 18 exhibited significant inhibitory activity on TGF-β/SMAD pathway at a concentration of 50 μM in HepG2 cells (Fig. 5b). Two compounds (24 and 26) revealed their autophagy modulating activities by inducing autophagic puncta and upregulating the autophagy marker LC3-II levels in HEK293 cells (Fig. 5c/d).

## Conclusions

In conclusion, ten new *Daphniphyllum* alkaloids, calycindaphines A–J (1–10), together with 17 known alkaloids were isolated from the roots of *D. calycinum*. Compound 1 is a novel alkaloid with a new C_22_ skeleton with a rare 5/8/7/5/5 ring system containing a unique 1-azabicyclo[5.2.1]decane. Compound 2 is the second example for the unique skeleton with 5/6/5/8/5/5 ring system. Compound 10 is the first example of secodaphniphylline-type alkaloid absent of the oxygen-bridge between C-25/C-29.

Compounds 16, 18, 22–24, and 26 exhibited their potential bioactivities on NF-κB or TGF-β inhibition and/or cell autophagic induction. Our findings not only revealed the chemicals from the roots of *D. calycinum* for the first time, but also give a new insight into the complex polycyclic skeletons and structural diversity of *Daphniphyllum* alkaloids.

## Experimental section

### General experimental procedures

Optical rotations were measured on a Rudolph Research Analytical Autopol I automatic polarimeter. Ultraviolet (UV) and CD spectra were recorded on a Jasco J-1500 Circular Dichroism Spectrometer. IR spectra were carried on an Agilent Cary 660 series FT-IR spectrometer (KBr). HRESIMS spectra were obtained on an Agilent 6230 HRESIMS spectrometer. 1D and 2D NMR spectra were performed on a Bruker Ascend 600 NMR spectrometer. The chemical shifts were expressed in *δ* (ppm) with TMS as an internal reference. Column chromatography was performed on Silica gel (40–60 mesh, Grace, USA) column. Thin layer chromatography was carried on precoated silica gel 60 F_254_ plates (200 μm thick, Merck KGaA, Germany). MPLC was performed using a Buchi Sepacore flash system with a RP-18 column (SilicBond C_18_, 36 × 460 mm ID, 40–63 μm particle size). Semi-preparative HPLC was conducted on an Agilent 1100/1200 liquid chromatography instrument with a Waters Xbridge Prep C_18_ column (10 × 250 mm, 5 μm) or Xbridge Prep C_8_ column (10 × 250 mm, 5 μm). UHPLC analyses were conducted on an Agilent 1290 system using a ZORBAX RRHD Eclipse Plus C_18_ column (1.8 μm, 2.1 × 50 mm, Agilent).

### Plant material

The roots of *D. calycinum* were collected from Guigang city of Guangxi Province, People's Republic of China, in October 2018, and identified by one of the authors, Dr Zhu G.-Y. A voucher specimen (DC-201810) was deposited at State Key Laboratory of Quality Research in Chinese Medicine, Macau University of Science and Technology.

### Extraction and isolation

The air-dried, powdered roots (30 kg) of *D. calycinum* were extracted three times with 80% EtOH. The combined filtrates were concentrated under vacuum to afford a dark extract, which was adjusted to pH 2 with HCl. The acidic mixture was centrifuged to remove dark brown precipitates. The aqueous layer was basified to pH 10 with NaHCO_3_ and then exhaustively extracted with EtOAc to afford the crude alkaloids (170.0 g). The crude alkaloids were subjected to a Silica gel column (CHCl_3_/MeOH, 1 : 0–0 : 1) to obtain 10 fractions (Fr.A–Fr.F).

Fr.A (22.5 g) was further chromatographed on a silica gel (40–60 mesh) column (CHCl_3_/MeOH, 1 : 0–1 : 20) to give 10 subfractions (Fr.A1–Fr.A10). Compounds 16 (560.0 mg) and 17 (11.0 mg) were isolated and purified by semipreparative reversed-phase (RP) HPLC with C-18 column eluted with 75% MeCN/0.1% DEA from the fraction Fr.A1. The fraction Fr.A2 was separated into two subfractions (Fr.A2a and Fr.A2b) by MPLC on C-18 column eluting with a gradient of MeCN/0.1% DEA–H_2_O (20 : 80 to 100 : 0, v/v). Fr.A2a was subjected to RP-HPLC with C-18 column (75% MeCN/0.1% DEA) to obtain 4 (6.2 mg). Fr.A2b was purified by semi-preparative HPLC with 72% MeCN/0.1% DEA to give 5 (29.0 mg) and 6 (5.2 mg). Fr.A3 was submitted to CC on silica gel to yield the subfraction (Fr.A3a). Fr.A3a was further purified by RP-HPLC with C-8 column eluted with 80% MeCN/0.1% DEA to afford 18 (95.0 mg), 24 (11.1 mg), 25 (11.0 mg), and 26 (35.0 mg). Fr.A4 was purified by semi-preparative HPLC with a C-18 column to give 22 (88.0 mg). Compound 27 (24.0 mg) was acquired by means of recrystallization from Fr.A10.

Fr.B (20.1 g) was subjected to MPLC on C-18 column and eluted with a gradient of MeCN/0.1% DEA–H_2_O (20 : 80 to 100 : 0, v/v) to give four subfractions (Fr.B1–Fr.B4). Fr.B1 was isolated by semi-preparative HPLC with C-18 column (60% MeCN/0.1% DEA) to obtain three alkaloids, 2 (3.6 mg), 19 (76.8 mg), and 20 (16.3 mg). Fr.B2 was separated by RP-HPLC with C-18 column (75% MeCN/0.1% DEA) to yield Fr.B2a and Fr.B2b. Fr.B2a was purified by RP-HPLC with C-18 column (54% MeCN/0.1% DEA) to give 7 (3.1 mg). Fr.B2b was purified by RP-HPLC (62% MeCN/0.1% DEA) to give 1 (7.0 mg). Fr.B3 was isolated by RP-HPLC with C-8 column (65% MeCN/0.1% DEA) to yield 13 (15.7 mg). Fr.C (19.3 g) was isolated by the MPLC on C-18 to give Fr.C8 and then was purified by RP-HPLC with C-18 column (55% MeCN/0.1% DEA) to afford 12 (2.6 mg). Fr.D (9.2 g) was separated by MPLC with a C-18 column and eluted with a gradient of MeCN/0.1% DEA–H_2_O (10 : 80 to 100 : 0, v/v) to give the main subfraction of Fr.D1. And then, the subfraction was separated by the RP-HPLC with C-18 column (58% MeCN/0.1% DEA) to afford 3 (2.5 mg), 9 (4.0 mg), 11 (16.0 mg), 14 (3.6 mg), 15 (45.0 mg), 21 (6.2 mg), and 23 (22.0 mg). Alkaloids of Fr.F (6.0 g) were enriched by the MPLC to give the main subfraction Fr.F7 which was purified by RP-HPLC with C-18 column to obtained 8 (2.7 mg) and 10 (5.2 mg).

#### Calycindaphine A (1)

White amorphous powder; [*α*]^22.5^_D_ −12.5 (*c* = 0.5, MeOH); UV (MeOH) *λ*_max_ (log *ε*): 200.4 (7.45), 304.8 (0.620) nm; IR (KBr) *ν*_max_: 3730, 3700, 3625, 3595, 3444, 2956, 1768, 1708, 1448, 1388, 1266, 1200, 754 cm^−1^; ECD (MeOH) *λ*_max_ (log *ε*): 217.2 (−51.89), 270.6 (32.30), 352.2 (−36.27) nm; ^1^H NMR and ^13^C NMR see Tables 1 and 2; HRESIMS *m*/*z* 370.2340 [M + H]^+^ (calcd for C_23_H_31_NO_4_ 370.2375).

#### Calycindaphine B (2)

White amorphous powder; [*α*]^22.5^_D_ −39.8 (*c* = 0.5, MeOH); UV (MeOH) *λ*_max_ (log *ε*): 201.1 (6.66), 346.1 (1.24) nm; IR (KBr) *ν*_max_: 3730, 3595, 3442, 2929, 1733, 1645, 1562, 1442, 1387, 1268, 1169, 754 cm^−1^; ECD (MeOH) *λ*_max_ (log *ε*): 200.4 (−52.76), 226.8 (166.29) nm; ^1^H NMR and ^13^C NMR see Tables 1 and 2; HRESIMS *m*/*z* 386.2324 [M + H]^+^ (calcd for C_23_H_32_NO_4_ 386.2326).

#### Calycindaphine C (3)

White amorphous powder; [*α*]^22.5^_D_ −52.9 (*c* = 0.5, MeOH); UV (MeOH) *λ*_max_ (log *ε*): 201.3 (1.26), 304.8 (0.66) nm; IR (KBr) *ν*_max_: 3730, 3701, 3626, 3596, 3449, 2933, 2865, 1735, 1649, 1545, 1393, 1358, 756 cm^−1^; ECD (MeOH) *λ*_max_ (log *ε*): 214.2 (−33.48), 247.8 (5.28), 283.2 (−13.25) nm; ^1^H NMR and ^13^C NMR see Tables 1 and 2; HRESIMS *m*/*z* 400.3485 [M + H]^+^ (calcd for C_24_H_34_NO_4_ 400.2482).

#### Calycindaphine D (4)

White amorphous powder; [*α*]^22.5^_D_ −41.5 (*c* = 0.5, MeOH); UV (MeOH) *λ*_max_ (log *ε*): 199.5 (12.9) nm; IR (KBr) *ν*_max_: 3730, 3701, 3625, 3596, 3380, 2949, 2858, 1770, 1699, 1546, 1425, 1388, 1350, 1268, 1128, 1093, 1043, 754 cm^−1^; ECD (MeOH) *λ*_max_ (log *ε*): 209.5 (533.12), 230.2 (−181.22) nm; ^1^H NMR and ^13^C NMR see Tables 1 and 2; HRESIMS *m*/*z* 372.2166 [M + H]^+^ (calcd for C_22_H_30_NO_4_ 372.2169).

#### Calycindaphine E (5)

White amorphous powder; [*α*]^22.5^_D_ −40.5 (*c* = 0.5, MeOH); UV (MeOH) *λ*_max_ (log *ε*): 204.1 (8.95) nm; IR (KBr) *ν*_max_: 3730, 3701, 3625, 3595, 3426, 2929, 1730, 1650, 1484, 1434, 1355, 1316, 1280, 1170, 1036, 741 m^−1^; ECD (MeOH) *λ*_max_ (log *ε*): 205.2 (271.64), 230.4 (−310.91) nm; ^1^H NMR and ^13^C NMR see Tables 1 and 2; HRESIMS *m*/*z* 388.2480 [M + H]^+^ (calcd for C_23_H_34_NO_4_ 388.2482).

#### Calycindaphine F (6)

White amorphous powder; [*α*]^22.5^_D_ −26.3 (*c* = 0.5, MeOH); UV (MeOH) *λ*_max_ (log *ε*): 200.7 (4.12), 222.8 (6.62) nm; IR (KBr) *ν*_max_: 3730, 3701, 3625, 3595, 3446, 2927, 2875, 1725, 1680, 1447, 1380, 1270, 1173, 755 cm^−1^; ECD (MeOH) *λ*_max_ (log *ε*): 229.1 (−413.27), 256.8 (72.60) nm; ^1^H NMR and ^13^C NMR see Tables 1 and 2; HRESIMS *m*/*z* 402.2293 [M + H]^+^ (calcd for C_23_H_32_NO_5_ 402.2275).

#### Calycindaphine G (7)

White amorphous powder; [*α*]^22.5^_D_ −57.0 (*c* = 0.5, MeOH); UV (MeOH) *λ*_max_ (log *ε*): 202.4 (4.54), 264.8 (1.03) nm; IR (KBr) *ν*_max_: 3730, 3701, 3626, 3596, 3446, 3927, 1731, 1644, 1457, 1391, 1271, 755 cm^−1^; ECD (MeOH) *λ*_max_ (log *ε*): 198.0 (−217.81), 234.6 (68.96), 264.0 (−150.08) nm; ^1^H NMR and ^13^C NMR see Tables 1 and 2; HRESIMS *m*/*z* 386.2332 [M + H]^+^ (calcd for C_23_H_32_NO_4_ 386.2326).

#### Calycindaphine H (8)

White amorphous powder; [*α*]^22.5^_D_ +26.8 (*c* = 0.5, MeOH); IR (KBr) *ν*_max_: 3866, 3730, 3701, 3620, 3596, 3447, 2926, 2863, 2379, 2316, 1697, 1650, 1570, 1549, 1518, 1385, 1055, 751, 464, 451 cm^−1^; ECD (MeOH) *λ*_max_ (log *ε*): 223.2 (10.89), 265.8 (−14.92), 294.6 (−4.72), 313.2 (10.71) nm; ^1^H NMR and ^13^C NMR see Tables 1 and 2; HRESIMS *m*/*z* 472.3786 [M + H]^+^ (calcd C_30_H_50_NO_3_ for 472.3785).

#### Calycindaphine I (9)

White amorphous powder; [*α*]^22.5^_D_ −36.8 (*c* = 0.5, MeOH); UV (MeOH) *λ*_max_ (log *ε*): 202.4 (3.28), 300.5 (1.76) nm; IR (KBr) *ν*_max_: 3730, 3701, 3625, 3595, 3446, 2934, 2868, 2316, 1703, 1647, 1545, 1458, 1392, 1268, 755 cm^−1^; ECD (MeOH) *λ*_max_ (log *ε*): 210.6 (−29.16), 232.2 (−16.40), 288.6 (−16.97) nm; ^1^H NMR and ^13^C NMR see Tables 1 and 2; HRESIMS *m*/*z* 486.3562 [M + H]^+^ (calcd for C_30_H_48_NO_4_ 486.3578).

#### Calycindaphine J (10)

White amorphous powder; [*α*]^22.5^_D_ −33.7 (*c* = 0.5, MeOH); IR (KBr) *ν*_max_: 3730, 3701, 3625, 3595, 3446, 2934, 2866, 1648, 1572, 1549, 1518, 1461, 1386, 1386, 1268, 1040, 752 cm^−1^; ^1^H NMR and ^13^C NMR see Tables 1 and 2; HRESIMS *m*/*z* 474.3958 [M + H]^+^ (calcd for C_30_H_52_NO_3_ 474.3942).

### ECD calculations of compound 1

Conformational analyses were carried out *via* random searching in the Sybyl-X 2.0 using the MMFF94S force field with an energy cutoff of 2.5 kcal mol^−1^. The results showed the nine lowest energy conformers for both compounds. Subsequently, the conformers were re-optimized using DFT at the PBE0-D3(BJ)/def2-SVP level in MeOH using the polarizable conductor calculation model (SMD) by the ORCA4.2.1 program. The energies, oscillator strengths, and rotational strengths (velocity) of the first 60 electronic excitations were calculated using the TDDFT methodology at the PBE0/def2-TZVP level in MeOH. The ECD spectra were simulated by the overlapping Gaussian function (half the bandwidth at 1/*e* peak height, sigma = 0.30 for all). To get the final spectra, the simulated spectra of the conformers were averaged according to the Boltzmann distribution theory and their relative Gibbs free energy (Δ*G*).

### Cell lines and cell cultures

The HepG2-NF-κB-Luc cell line is stably transfected with the NF-κB-luciferase gene, which was generously provided by Dr C. H. Leung (University of Macau). Cells were cultivated with DMEM medium supplemented with 10% fetal bovine serum (FBS), 100 U mL^−1^ penicillin, and 100 μg mL^−1^ streptomycin at 37 °C with 5% CO_2_ and 95% air incubator.

SMAD 2/3 responsive luciferase reporter HepG2 stable cell line was purchased from Signosis. Cells were cultivated with DMEM medium supplemented with 5% FBS, 100 U mL^−1^ penicillin, 100 μg mL^−1^ streptomycin, and 100 μg mL^−1^ hygromycin B at 37 °C with 5% CO_2_ and 95% air incubator.

HEK293 cell line stable transfected with GFP-LC3 was kindly provided by Dr X. M. Zhu (Macau University of Science and Technology). The cells were cultured in an α-MEM medium supplemented with 10% FBS under a humidified atmosphere containing 5% CO_2_ at 37 °C.

### NF-κB luciferase assay

The NF-κB activity was determined by NF-κB luciferase assay as described in our previous publication with a slight modification.^32^ Briefly, HepG2-NF-κB-Luc cells were seeded on a 96-well microplate with 1 × 10^4^ cells per well and cultured at 37 °C with 5% CO_2_ incubator for 18 h. Then, cells were pretreated with compounds (6, 12.5, and 50 μM) for 12 h and induced with TNF-α (10 ng mL^−1^) for 4 h. Ammonium pyrrolidinedithiocarbamate (PDTC) was used as the positive control. The firefly luciferase signal was measured with the Bright-Glo Luciferase Reporter Assay System (Promega, Madison, WI) according to the manufacturer's instruction using a multimode reader (SpectraMax iD5, Danaher).

### TGF-β induced SMAD luciferase assay

The effects of compounds on the TGF-β/SMAD were determined by TGF-β/SMAD luciferase assay. Briefly, HepG2/SMAD-Luc cells were seeded on a 96-well microplate with 1 × 10^4^ cells per well and cultured at 37 °C with 5% CO_2_ incubator for 18 h. After adhesion, the cells were pretreated with compounds at different concentration for 6 h and induced with TGF-β (10 ng mL^−1^) for 18 h. Meanwhile, SB-431542, a specific inhibitor of TGF-β Receptor Kinase, was applied as the positive control. The firefly luciferase signal was measured with the Bright-Glo Luciferase Reporter Assay System (Promega, Madison, WI) according to the manufacturer's instruction using a multimode reader (SpectraMax iD5, Danaher).

### LC3 puncta counting

The HEK293-GFP-LC3 cells were applied for visualizing autophagosome formation after treatment with various compounds for 24 hours. During autophagosome formation, GFP-LC3 is processed and recruited to the autophagosome membrane, where it can be imaged as cytophasmic puncta by IncuCyte ZOOM live cell imaging (Olympus, coupled with Hamama Tsu ORCA-Flash 40 LT Plus Scientific CMOS Digital Camera). The percentage of GFP-LC3 positive cells can be determined and is indicative of autophagosome formation.

### Western blot analysis

Primary antibody against LC3II was purchased from Cell Signaling Technology. Primary antibody against glyceraldehyde-3-phosphate dehydrogenase (GAPDH) was purchased from Abcam Inc. (Cambridge, MA). Secondary antibodies, and goat anti-mouse/anti-rabbit IgG H&L (IRDye 800CW) were purchased from Abcam Inc. (Cambridge, MA).

The cells were seeded onto 6-well plates with α-MEM medium and cultured for 24 hours. After treating with various concentrations of compounds 24 and 26, cells were collected and lysed in lysis buffer on ice for 30 minutes. Protein samples were electrophoresed using 15% SDS-PAGE gel and transferred onto a nitrocellulose membrane (NC membrane). The membranes were blocked by a 5% BSA and incubated with the primary antibody overnight at 4 °C, followed by the secondary antibody for 1 hour at room temperature. Protein bands were detected by the LI-COR Odyssey imaging system (Lincoln, NE).

## Conflicts of interest

There are no conflicts to declare.

## Supplementary Material

RA-011-D1RA00107H-s001
