# Supplementary material for: Calycindaphines A–J, Daphniphyllum alkaloids from the roots of Daphniphyllum calycinum
Source: RSC Adv. 2021 Mar 1;11(16):9057–66. doi: 10.1039/d1ra00107h (PMC8695325; doi:10.1039/d1ra00107h)
Supplement: RA-011-D1RA00107H-s001 [file RA-011-D1RA00107H-s001.pdf]

Electronic Supplementary Material (ESI) for RSC Advances.
This journal is © The Royal Society of Chemistry 2021

Supporting Information

Calycindaphines A–J, *Daphniphyllum* alkaloids from the roots of *Daphniphyllum calycinum*

Ji Yang,[a] Xin Liu,[a,b] Jing Fu,[a] Hao-Yuan Lyu,[a] Li-Ping Bai,[a] Zhi-Hong Jiang,*[a] and Guo-Yuan Zhu *[a]

[a] State Key Laboratory of Quality Research in Chinese Medicine, Macau Institute for Applied Research in Medicine and Health, Macau University of Science and Technology, Macau, China

[b] Biology Institute, Qilu University of Technology (Shandong Academy of Sciences), Jinan 250103, China

Fig. S1. HR-ESI-MS spectrum of compound **1** 6
Fig. S2. ^{1}H NMR spectrum for compound **1** 6
Fig. S3. ^{13}C and DEPT135 NMR spectra for compound **1** 7
Fig. S4. HSQC NMR spectrum for compound **1** 7
Fig. S5. ^{1}H–^{1}H COSY NMR spectrum for compound **1** 8
Fig. S6. HMBC NMR spectrum for compound **1** 8
Fig. S7. NOESY NMR spectrum for compound **1** 9
Fig. S8. IR spectrum of compound **1** (KBr) 9
Fig. S9. UV spectrum of compound **1** in MeOH 10
Fig. S10. CD spectrum of compound **1** in MeOH 10
Fig. S11. HR-ESI-MS spectrum of compound **2** 11
Fig. S12. ^{1}H NMR spectrum for compound **2** 11
Fig. S13. ^{13}C and DEPT135 NMR spectra for compound **2** 12
Fig. S14. HSQC NMR spectrum for compound **2** 12
Fig. S15. ^{1}H–^{1}H COSY NMR spectrum for compound **2** 13
Fig. S16. HMBC NMR spectrum for compound **2** 13
Fig. S17. NOESY NMR spectrum for compound **2** 14
Fig. S18. IR spectrum of compound **2** (KBr) 14
Fig. S19. UV spectrum of compound **2** in MeOH 15
Fig. S20. CD spectrum of compound **2** in MeOH 15
Fig. S21. HR-ESI-MS spectrum of compound **3** 16
Fig. S22. ^{1}H NMR spectrum for compound **3** 16
Fig. S23. ^{13}C and DEPT135 NMR spectra for compound **3** 17
Fig. S24. HSQC NMR spectrum for compound **3** 17
Fig. S25. ^{1}H–^{1}H COSY NMR spectrum for compound **3** 18
Fig. S26. HMBC NMR spectrum for compound **3** 18
Fig. S27. NOESY NMR spectrum for compound **3** 19
Fig. S28. IR spectrum of compound **3** (KBr) 19
Fig. S29. UV spectrum of compound **3** in MeOH 20
Fig. S30. CD spectrum of compound **3** in MeOH 20
Fig. S31. HR-ESI-MS spectrum of compound **4** 21

Fig. S32. ^{1}H NMR spectrum for compound **4** 21
Fig. S33. ^{13}C and DEPT135 NMR spectra for compound **4** 22
Fig. S34. HSQC NMR spectrum for compound **4** 22
Fig. S35. ^{1}H–^{1}H COSY NMR spectrum for compound **4** 23
Fig. S36. HMBC NMR spectrum for compound **4** 23
Fig. S37. NOESY NMR spectrum for compound **4** 24
Fig. S38. IR spectrum of compound **4** (KBr) 24
Fig. S39. UV spectrum of compound **4** in MeOH 25
Fig. S40. CD spectrum of compound **4** in MeOH 25
Fig. S41. HR-ESI-MS spectrum of compound **5** 26
Fig. S42. ^{1}H NMR spectrum for compound **5** 26
Fig. S43. ^{13}C and DEPT135 NMR spectra for compound **5** 27
Fig. S44. HSQC NMR spectrum for compound **5** 27
Fig. S45. ^{1}H−^{1}H COSY NMR spectrum for compound **5** 28
Fig. S46. HMBC NMR spectrum for compound **5** 28
Fig. S47. NOESY NMR spectrum for compound **5** 29
Fig. S48. IR spectrum of compound **5** (KBr) 29
Fig. S49. UV spectrum of compound **5** in MeOH 30
Fig. S50. CD spectrum of compound **5** in MeOH 30
Fig. S51. HR-ESI-MS spectrum of compound **6** 31
Fig. S52. ^{1}H NMR spectrum for compound **6** 31
Fig. S53. ^{13}C and DEPT135 NMR spectra for compound **6** 32
Fig. S54. HSQC NMR spectrum for compound **6** 32
Fig. S55. ^{1}H–^{1}H COSY NMR spectrum for compound **6** 33
Fig. S56. HMBC NMR spectrum for compound **6** 33
Fig. S57. NOESY NMR spectrum for compound **6** 34
Fig. S58. IR spectrum of compound **6** (KBr) 34
Fig. S59. UV spectrum of compound **6** in MeOH 35
Fig. S60. CD spectrum of compound **6** in MeOH 35
Fig. S61. HR-ESI-MS spectrum of compound **7** 36
Fig. S62. ^{1}H NMR spectrum for compound **7** 36
Fig. S63. ^{13}C and DEPT135 NMR spectra for compound **7** 37

Fig. S64. HSQC NMR spectrum for compound **7** 37
Fig. S65. ^1H–^1H COSY NMR spectrum for compound **7** 38
Fig. S66. HMBC NMR spectrum for compound **7** 38
Fig. S67. NOESY NMR spectrum for compound **7** 39
Fig. S68. IR spectrum of compound **7** (KBr) 39
Fig. S69. UV spectrum of compound **7** in MeOH 40
Fig. S70. CD spectrum of compound **7** in MeOH 40
Fig. S71. HR-ESI-MS spectrum of compound **8** 41
Fig. S72. ^1H NMR spectrum for compound **8** 41
Fig. S73. ^{13}C and DEPT135 NMR spectra for compound **8** 42
Fig. S74. HSQC NMR spectrum for compound **8** 42
Fig. S75. ^1H–^1H COSY NMR spectrum for compound **8** 43
Fig. S76. HMBC NMR spectrum for compound **8** 43
Fig. S77. NOESY NMR spectrum for compound **8** 44
Fig. S78. IR spectrum of compound **8** (KBr) 44
Fig. S79. UV spectrum of compound **8** in MeOH 45
Fig. S80. CD spectrum of compound **8** in MeOH 45
Fig. S81. HR-ESI-MS spectrum of compound **9** 46
Fig. S82. ^1H NMR spectrum for compound **9** 46
Fig. S83. ^{13}C and DEPT135 NMR spectra for compound **9** 47
Fig. S84. HSQC NMR spectrum for compound **9** 47
Fig. S85. ^1H−^1H COSY NMR spectrum for compound **9** 48
Fig. S86. HMBC NMR spectrum for compound **9** 48
Fig. S87. NOESY NMR spectrum for compound **9** 49
Fig. S88. IR spectrum of compound **9** (KBr) 49
Fig. S89. UV spectrum of compound **9** in MeOH 50
Fig. S90. CD spectrum of compound **9** in MeOH 50
Fig. S91. HR-ESI-MS spectrum of compound **10** 51
Fig. S92. ^1H NMR spectrum for compound **10** 51
Fig. S93. ^{13}C and DEPT135 NMR spectra for compound **10** 52
Fig. S94. HSQC NMR spectrum for compound **10** 52
Fig. S95. ^1H–^1H COSY NMR spectrum for compound **10** 53

Fig. S96. HMBC NMR spectrum for compound **10** 53

Fig. S97. NOESY NMR spectrum for compound **10** 54

Fig. S98. IR spectrum of compound **10** (KBr) 54

Fig. S99. UV spectrum of compound **10** in MeOH 55

Fig. S100. CD spectrum of compound **10** in MeOH 55

Fig. S101. Optimized conformers of compound **1** 56

Table S1. Gibbs free energiesa and equilibrium populationsb of low-energy conformers of **1**. 57

Table S2. Cartesian coordinates for the low-energy reoptimized random reseach conformers of **1** at PBE0-D3(BJ)/def2-SVP level of theory in methanol. 57

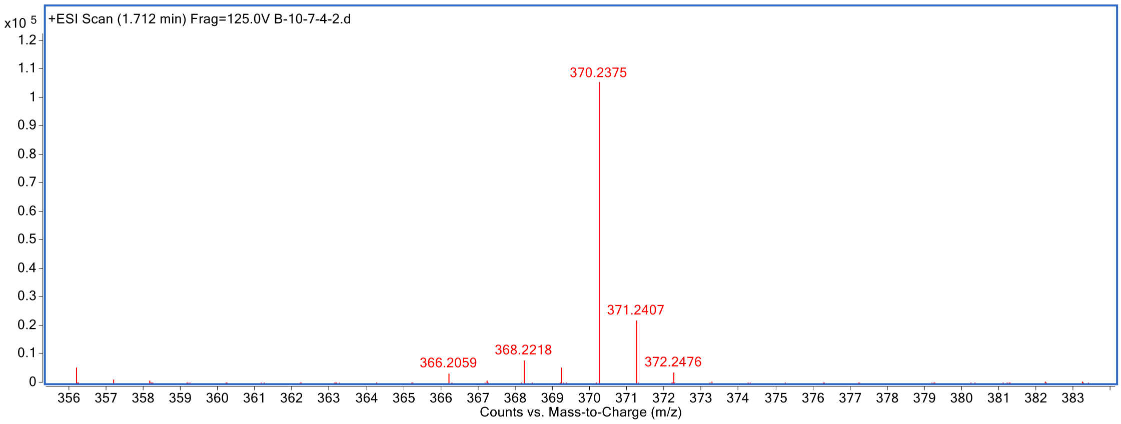

Formula (M)	Score (MFG)	Mass	Mass (MFG)	*m/z* (Calc)	Diff (ppm)	*m/z*
$C_{23}H_{31}NO_3$	99.92	369.2302	369.2304	370.2377	0.17	370.2375

Fig. S1. HR-ESI-MS spectrum of compound **1**

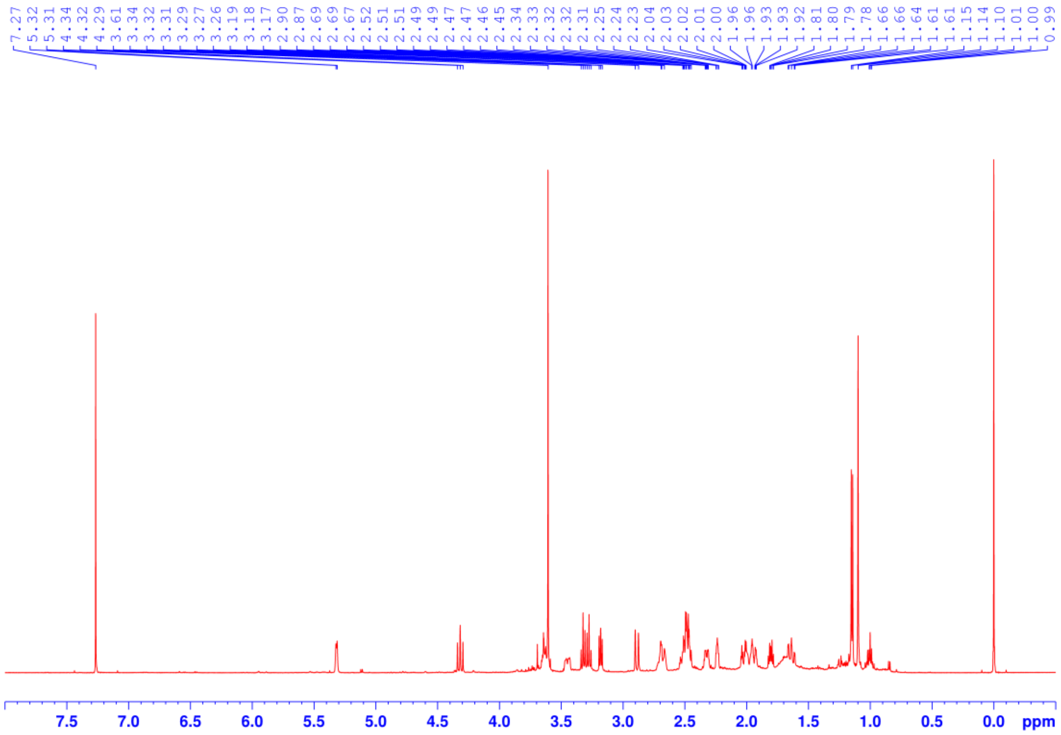

Fig. S2. ^{1}H NMR spectrum for compound **1**

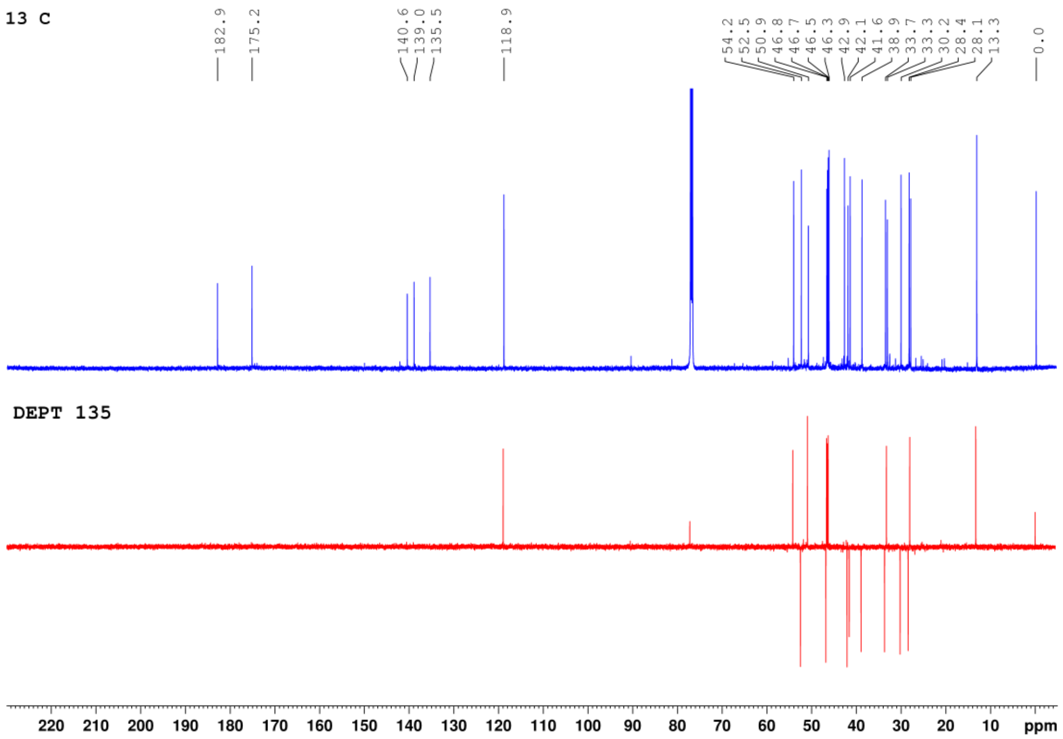

Fig. S3. ^{13}C and DEPT135 NMR spectra for compound **1**

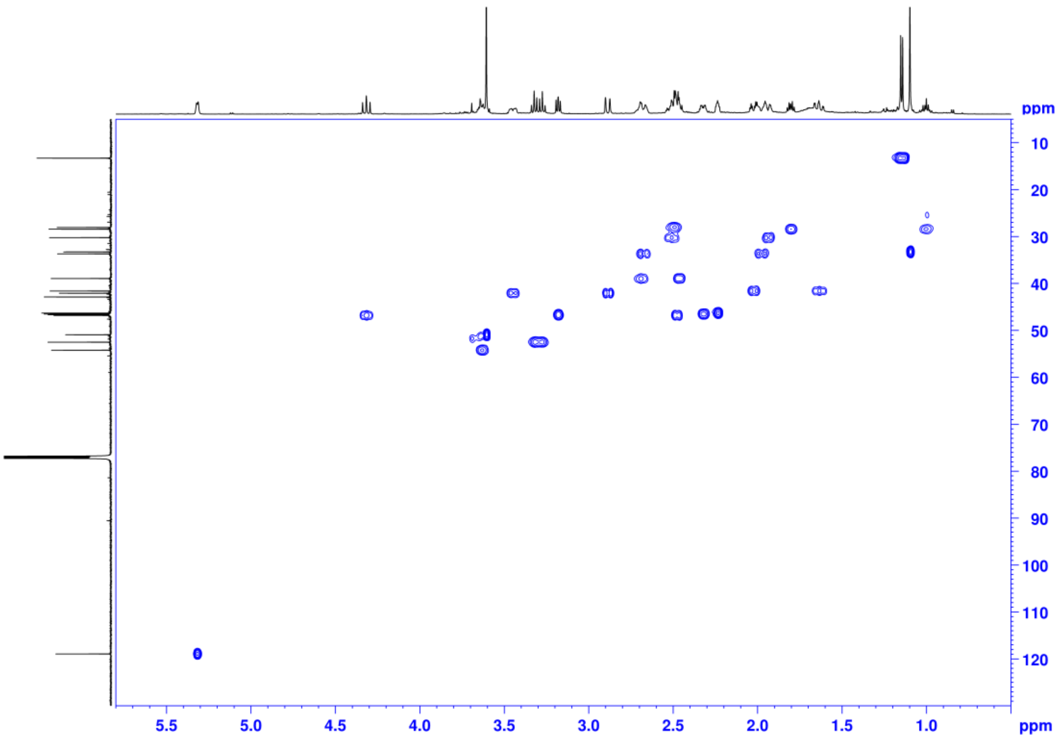

Fig. S4. HSQC NMR spectrum for compound **1**

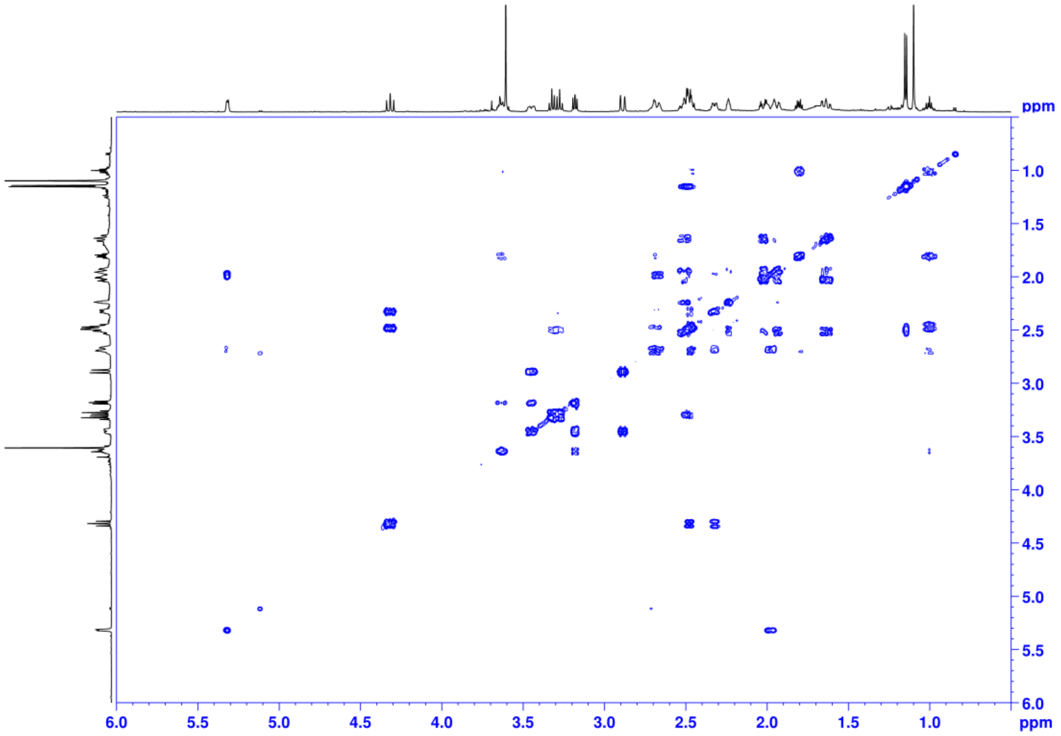

Fig. S5. ^{1}H–^{1}H COSY NMR spectrum for compound **1**

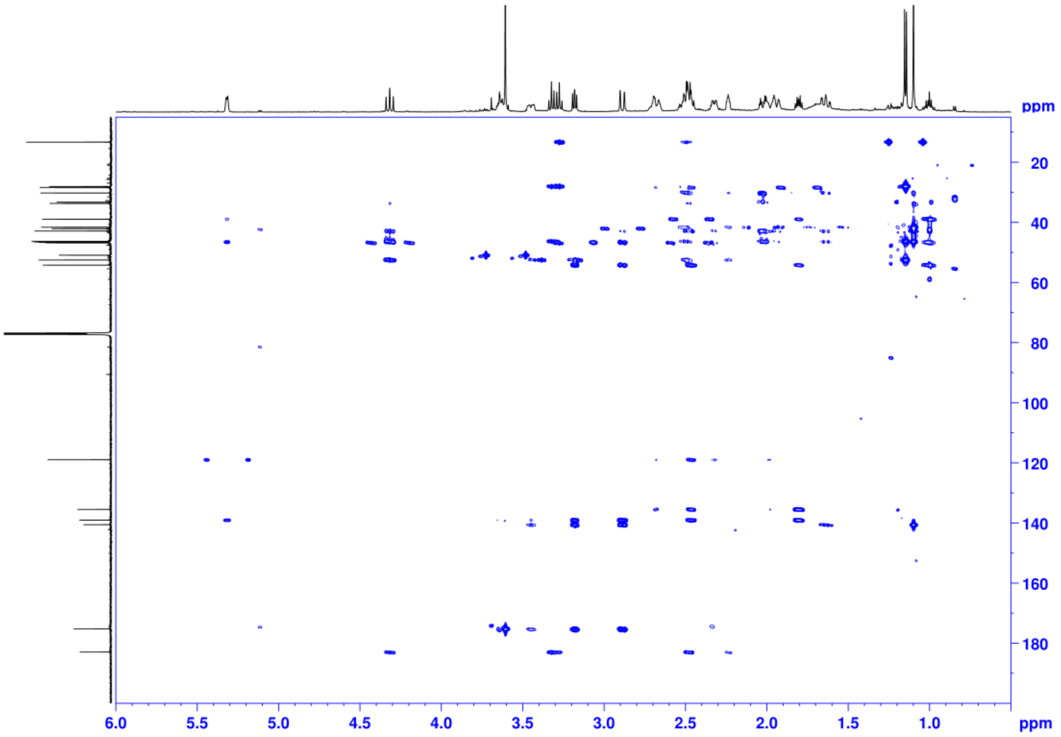

Fig. S6. HMBC NMR spectrum for compound **1**

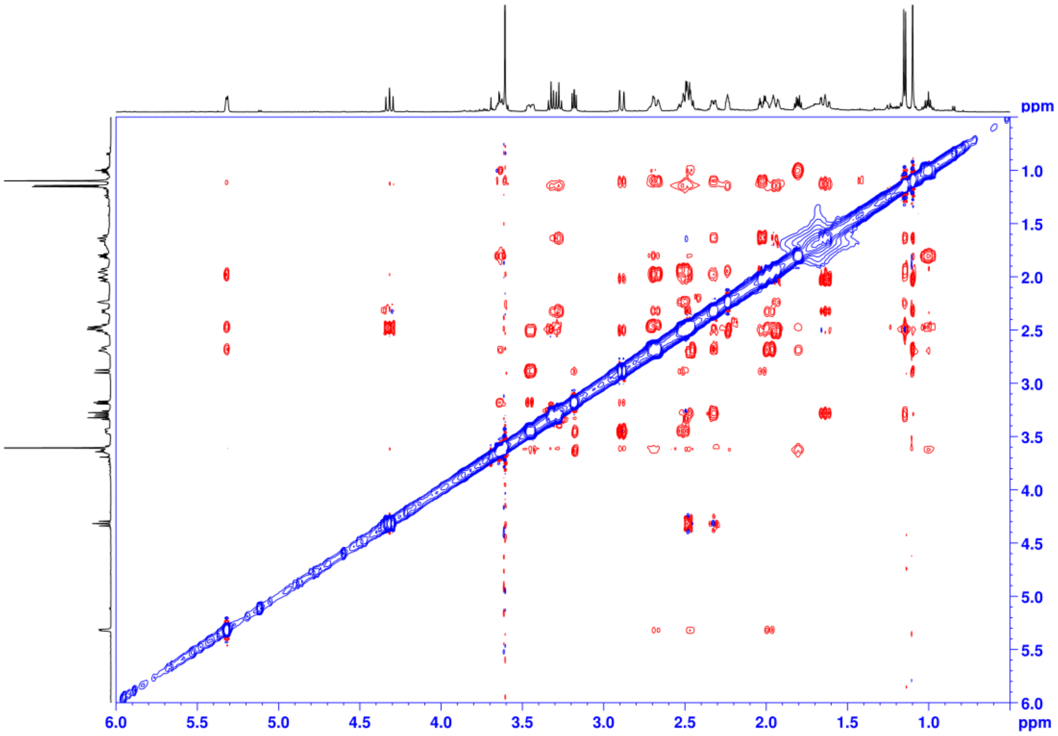

Fig. S7. NOESY NMR spectrum for compound **1**

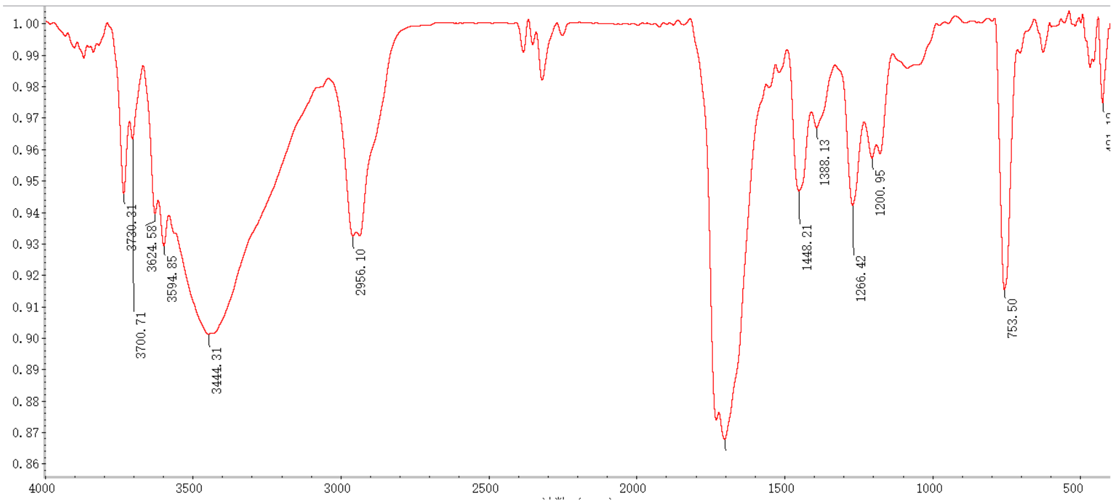

Fig. S8. IR spectrum of compound **1** (KBr)

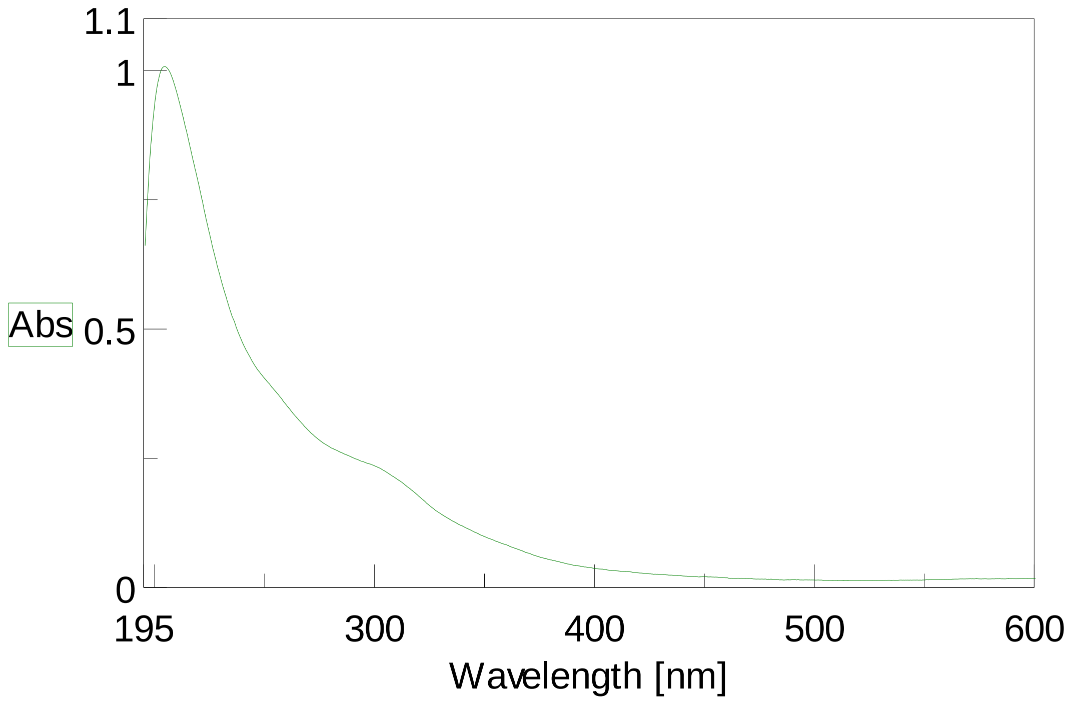

Fig. S9. UV spectrum of compound **1** in MeOH

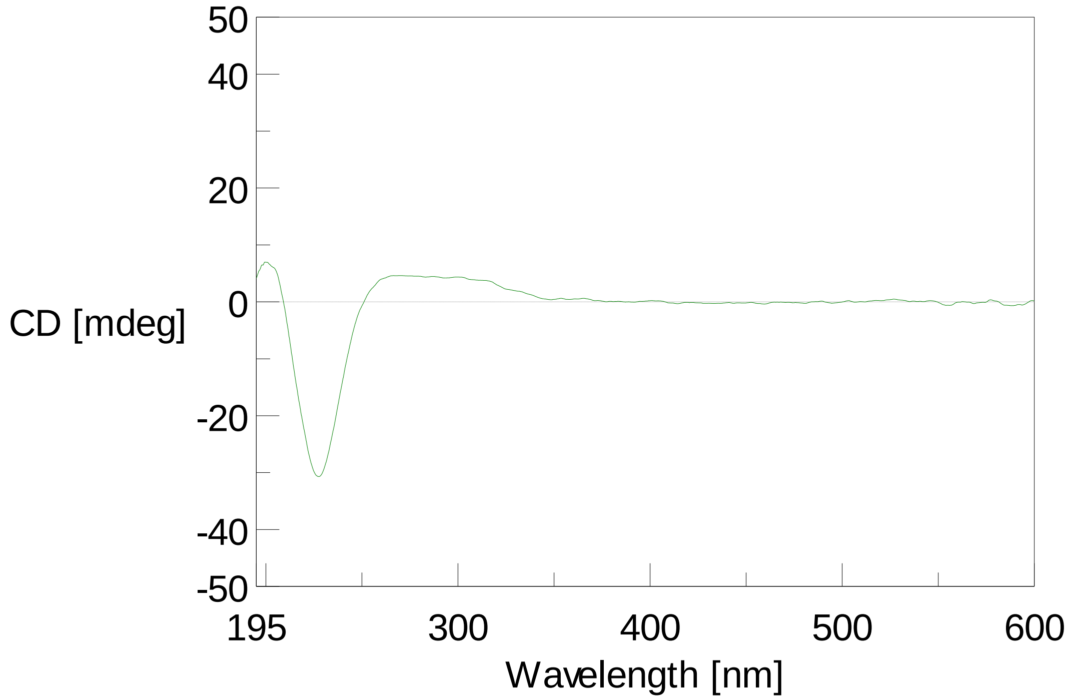

Fig. S10. CD spectrum of compound **1** in MeOH

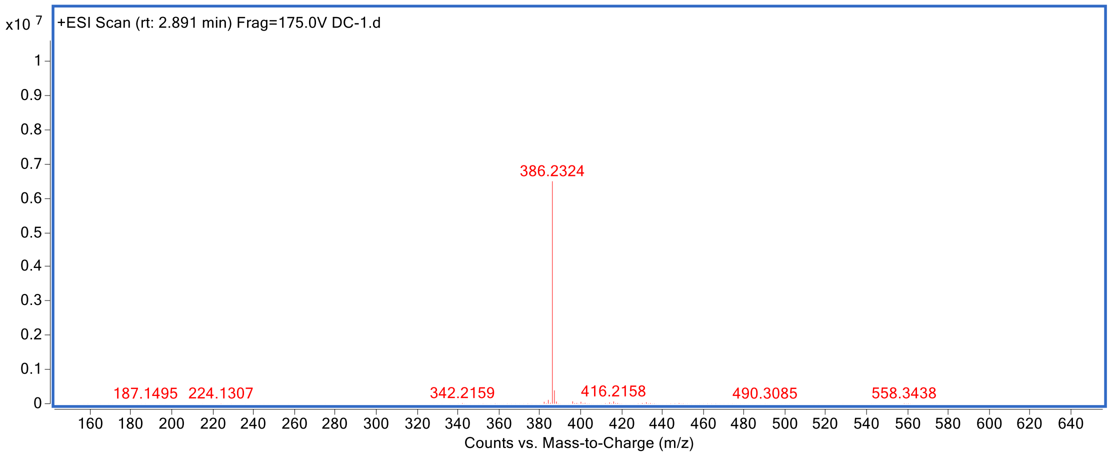

Formula (M)	Score (MFG)	Mass	Mass (MFG)	*m/z* (Calc)	Diff (ppm)	*m/z*
$C_{23}H_{31}NO_4$	99.91	385.2251	385.2253	386.2326	0.48	386.2324

Fig. S11. HR-ESI-MS spectrum of compound **2**

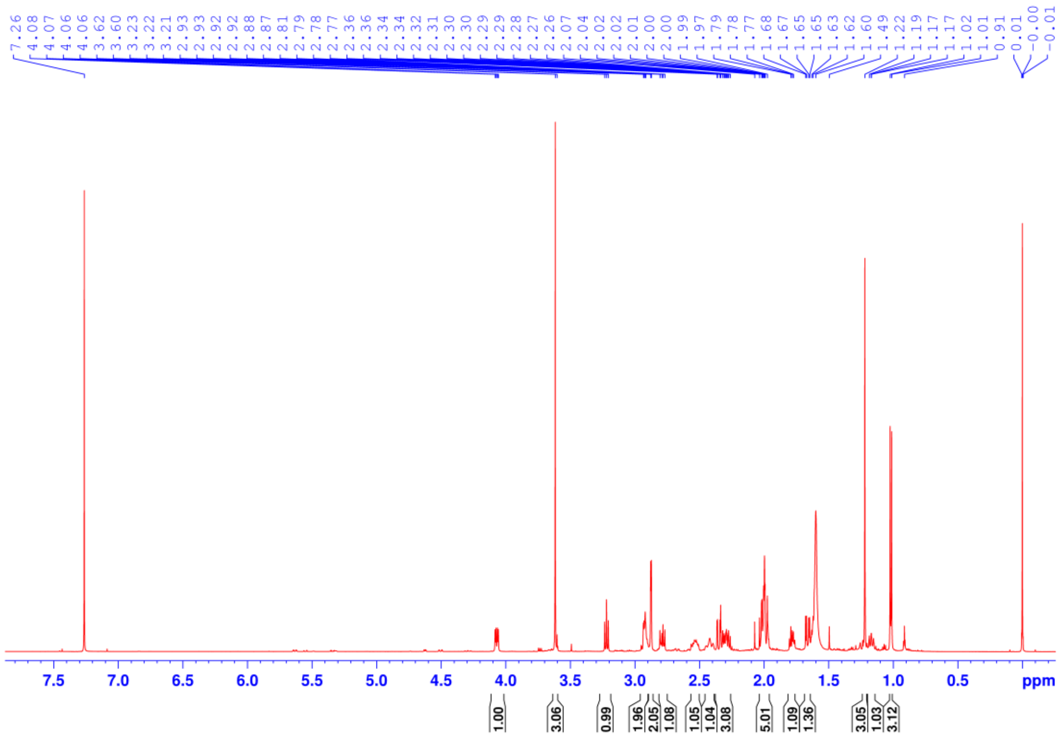

Fig. S12. ^{1}H NMR spectrum for compound **2**

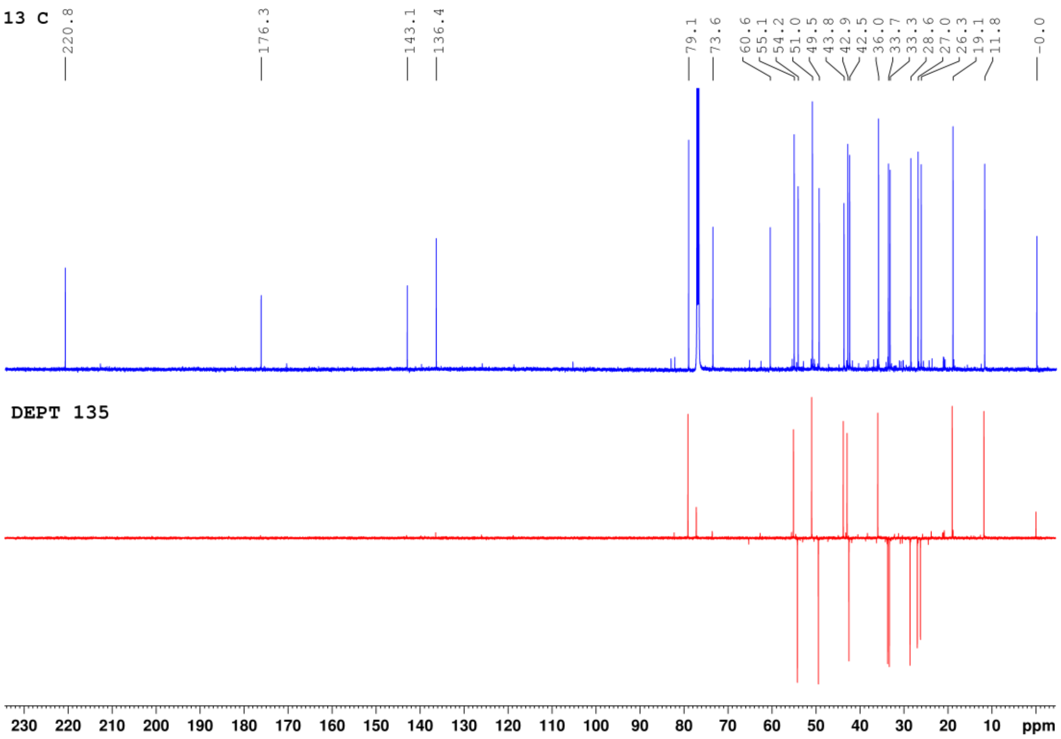

Fig. S13. ^{13}C and DEPT135 NMR spectra for compound **2**

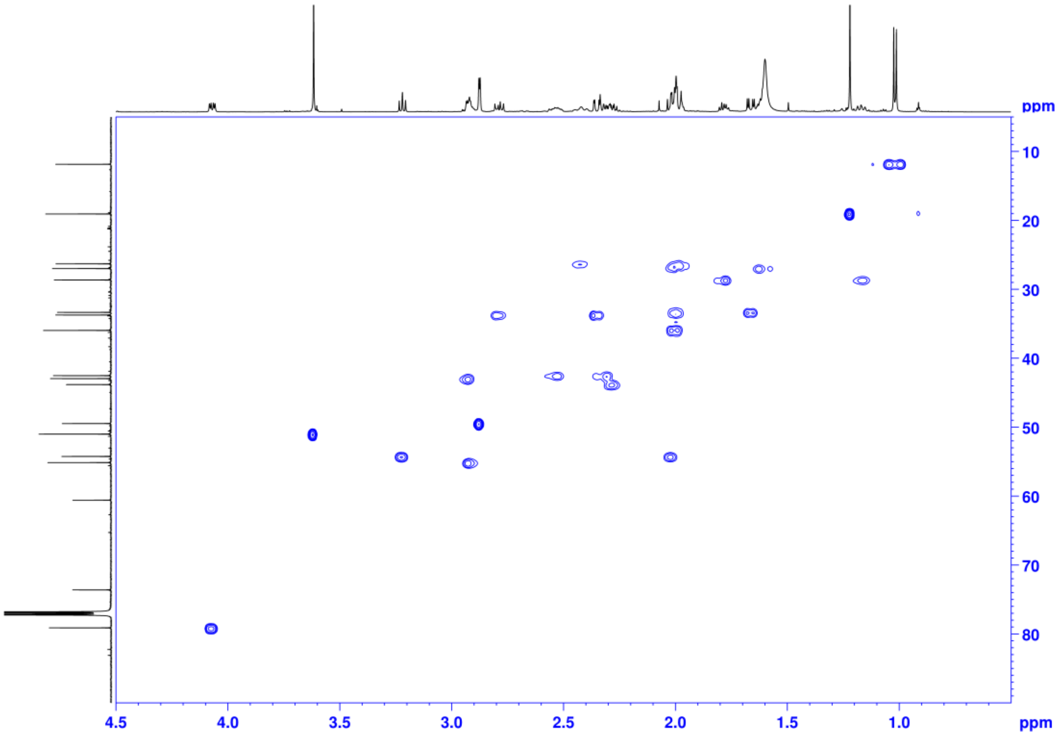

Fig. S14. HSQC NMR spectrum for compound **2**

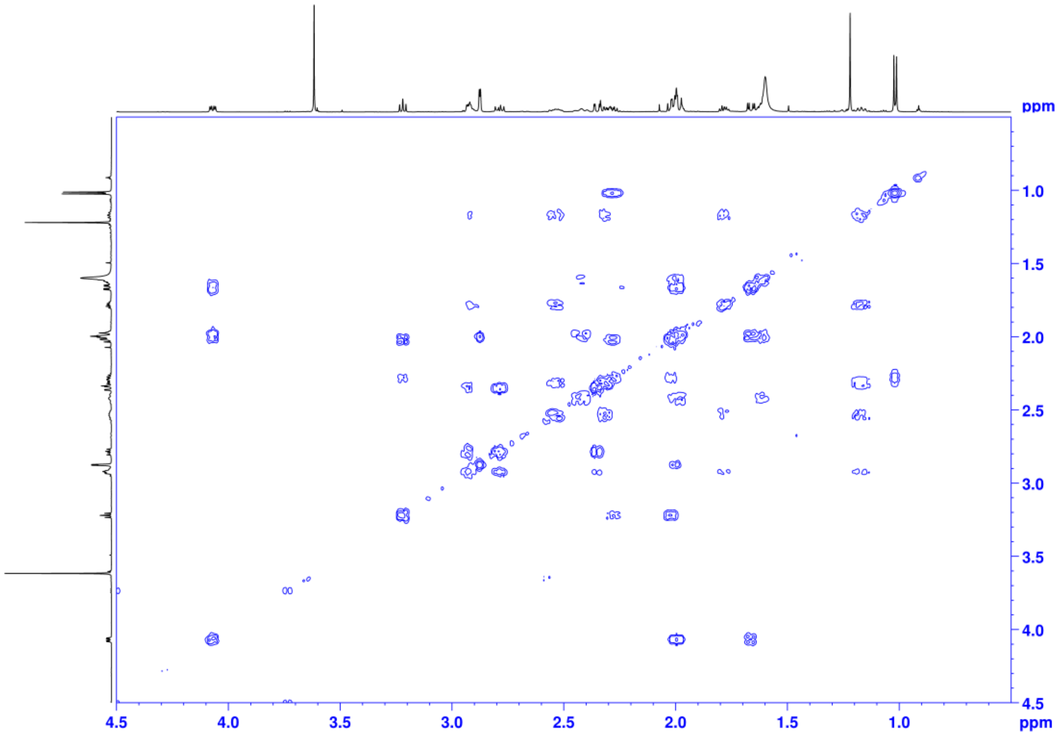

Fig. S15. ¹H–¹H COSY NMR spectrum for compound **2**

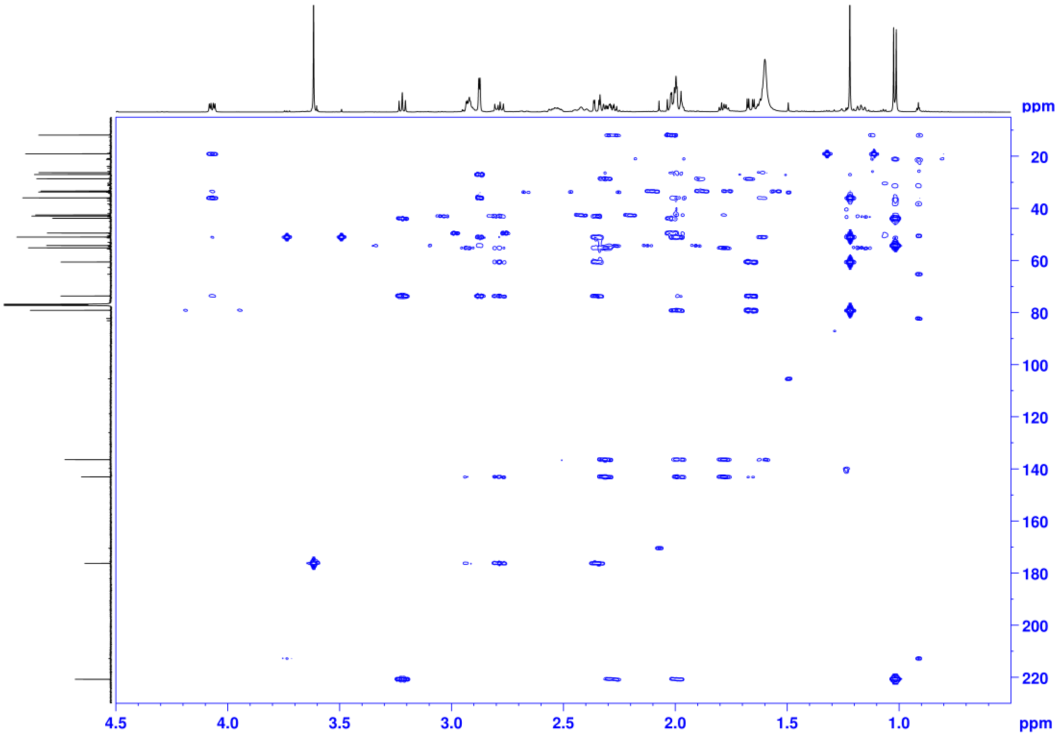

Fig. S16. HMBC NMR spectrum for compound **2**

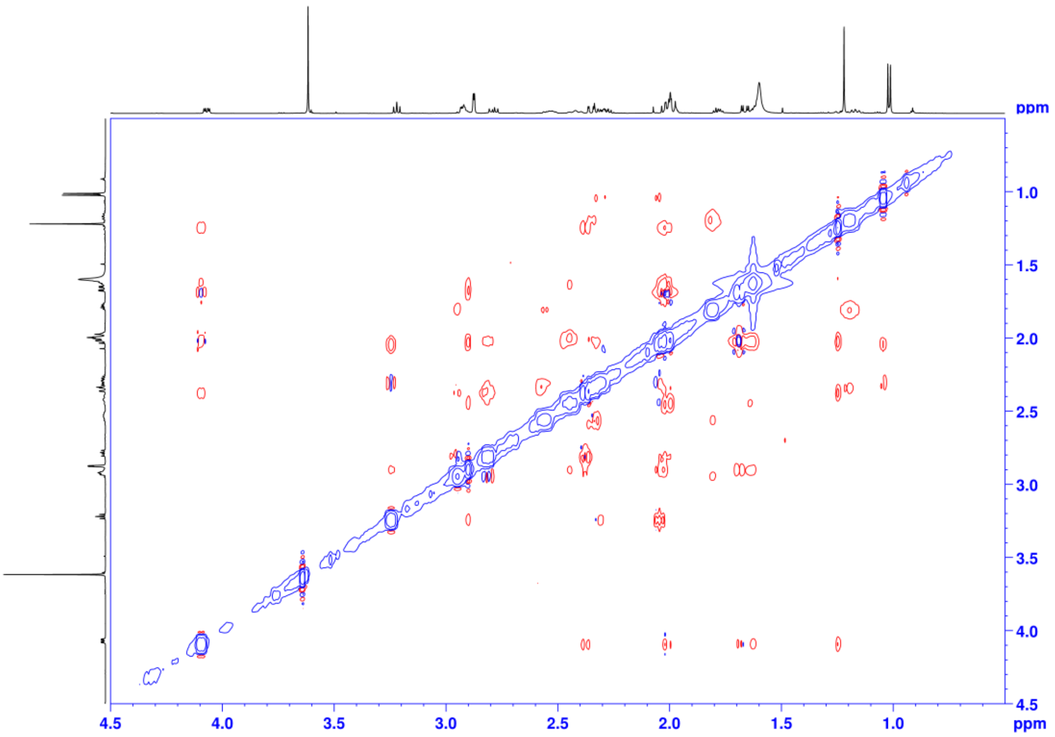

Fig. S17. NOESY NMR spectrum for compound **2**

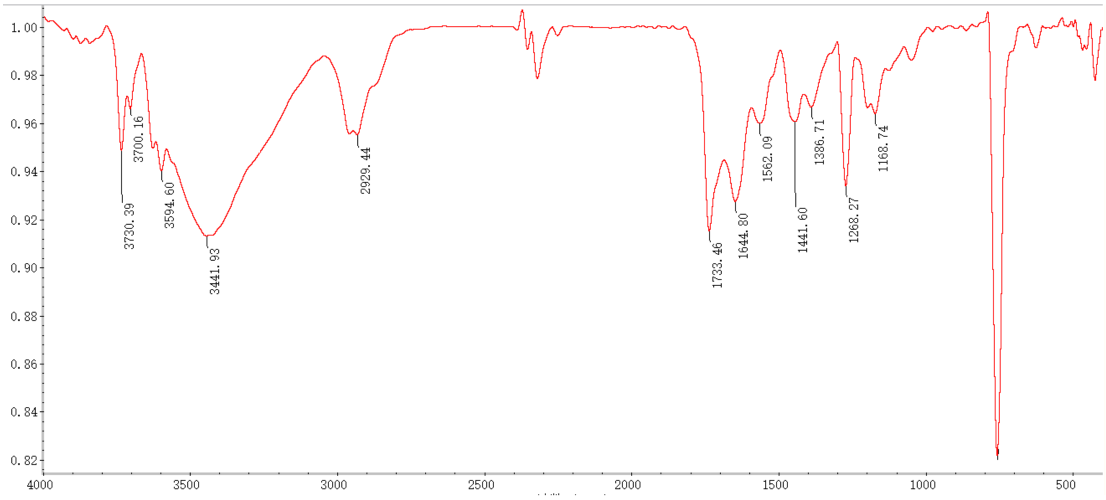

Fig. S18. IR spectrum of compound **2** (KBr)

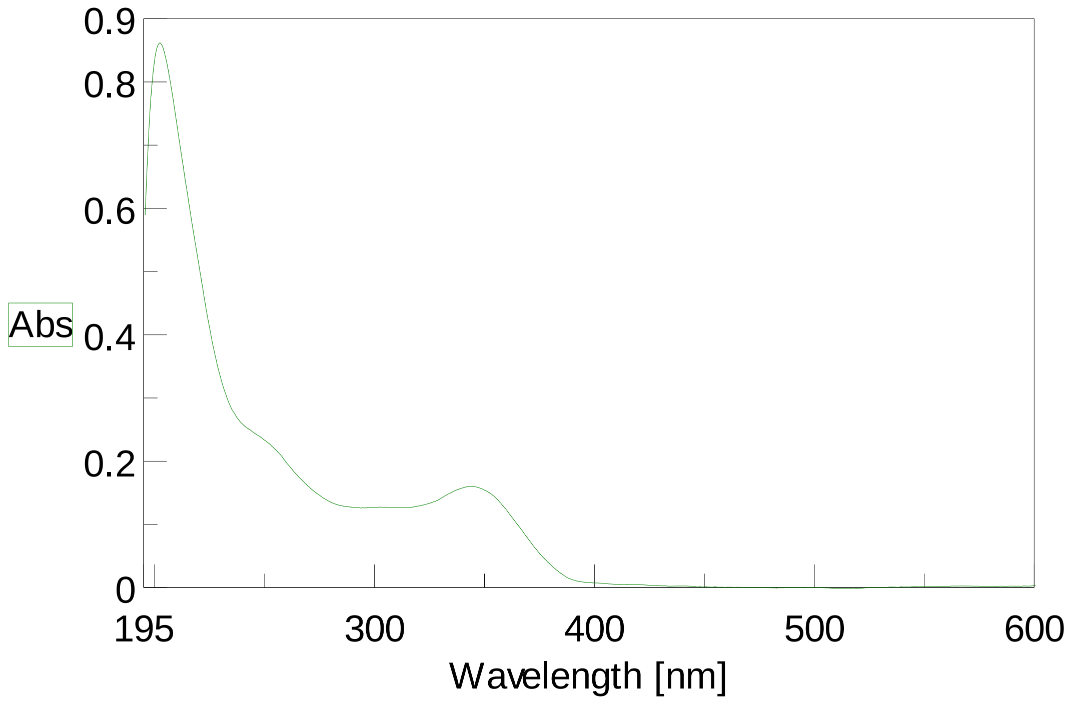

Fig. S19. UV spectrum of compound **2** in MeOH

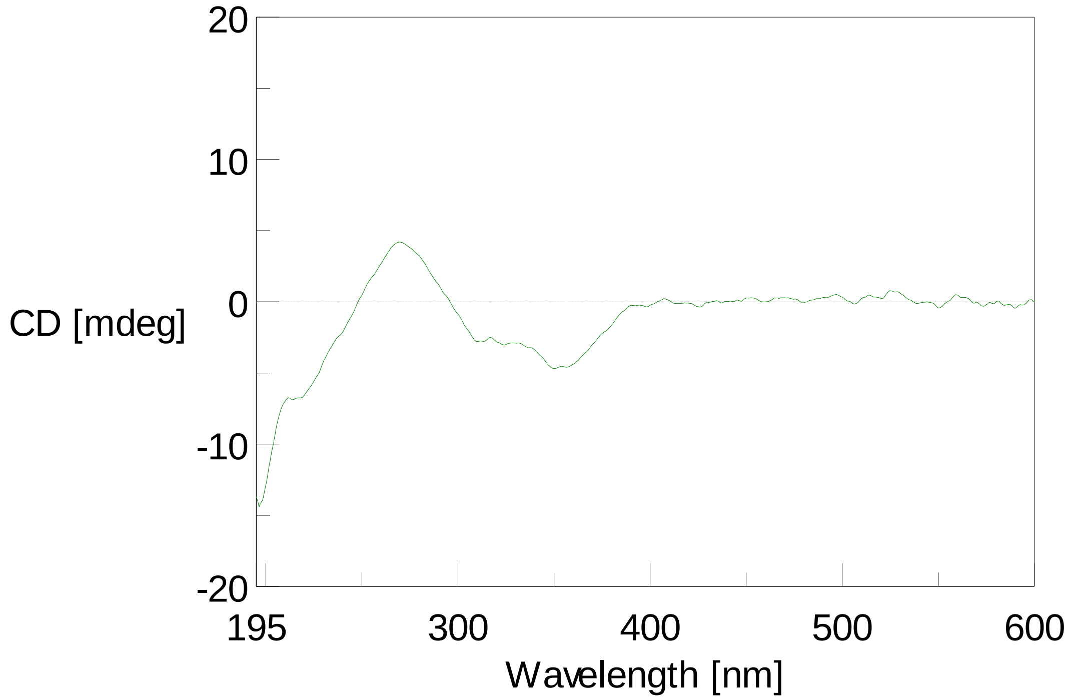

Fig. S20. CD spectrum of compound **2** in MeOH

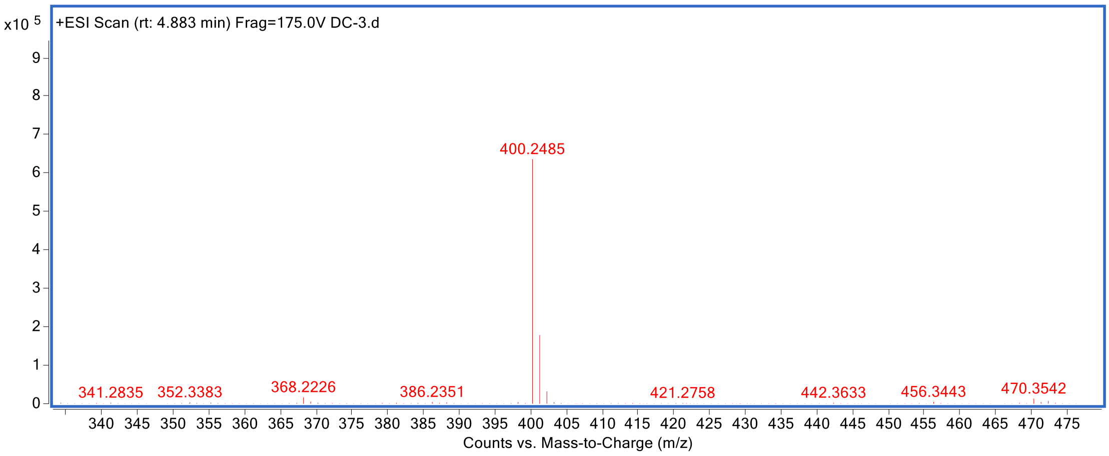

Formula (M)	Score (MFG)	Mass	Mass (MFG)	*m/z* (Calc)	Diff (ppm)	*m/z*
$C_{24}H_{33}NO_4$	99.81	399.2412	399.241	400.2482	-0.66	400.2485

Fig. S21. HR-ESI-MS spectrum of compound **3**

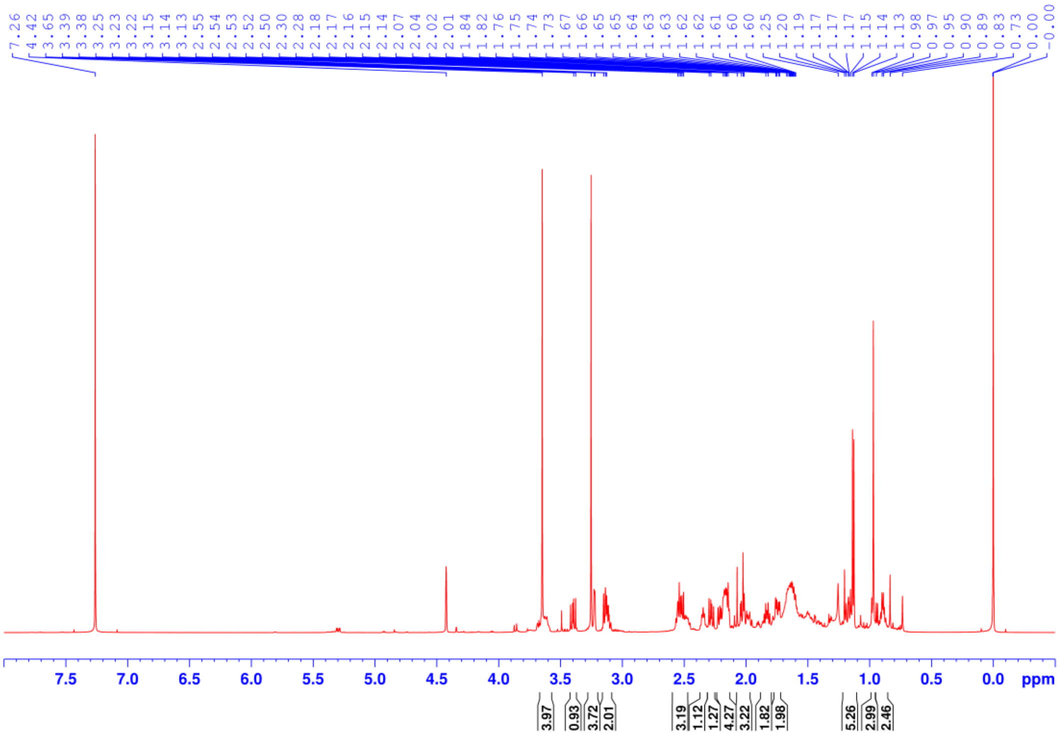

Fig. S22. ^{1}H NMR spectrum for compound **3**

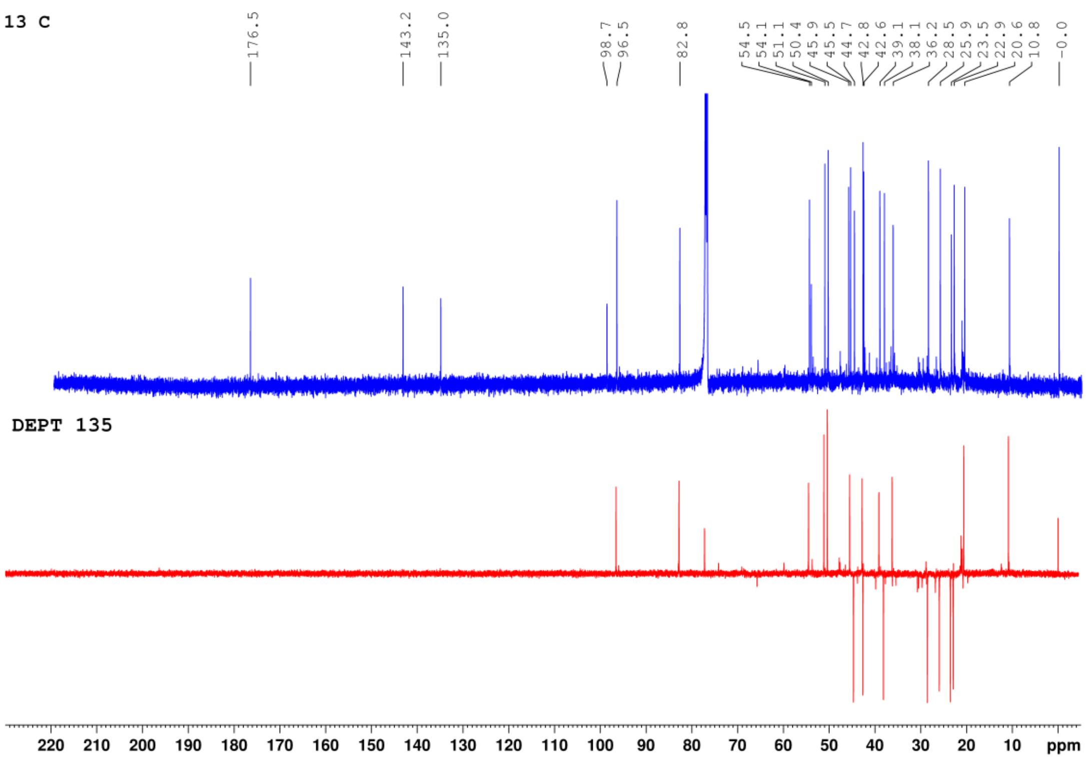

Fig. S23. ^{13}C and DEPT135 NMR spectra for compound **3**

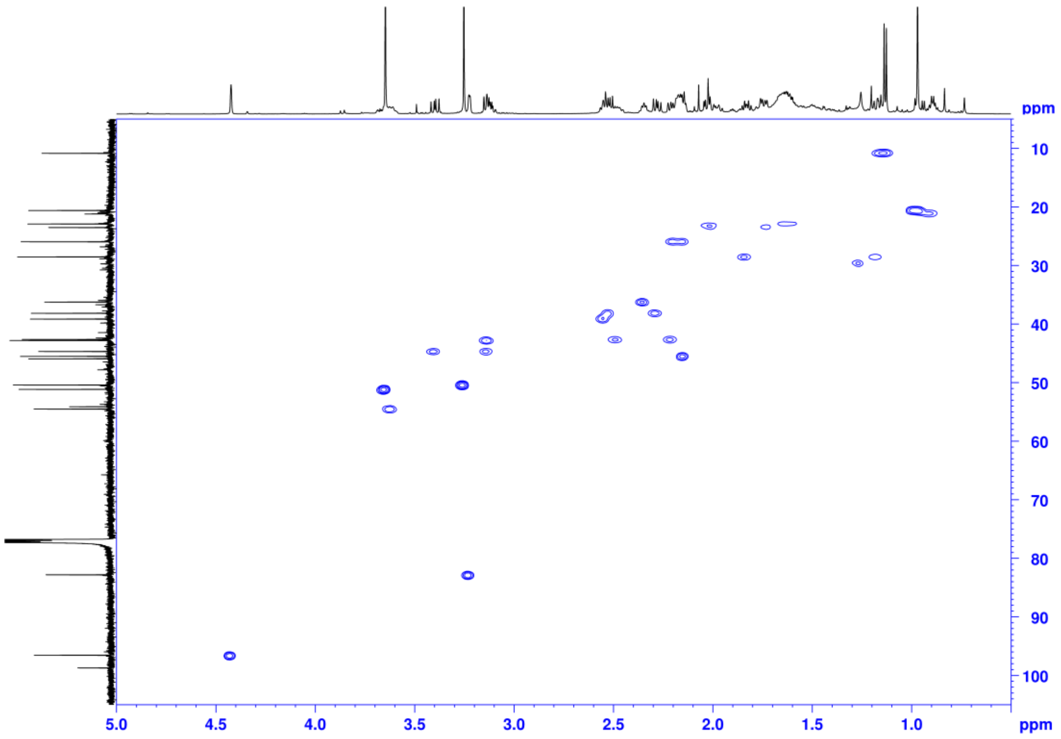

Fig. S24. HSQC NMR spectrum for compound **3**

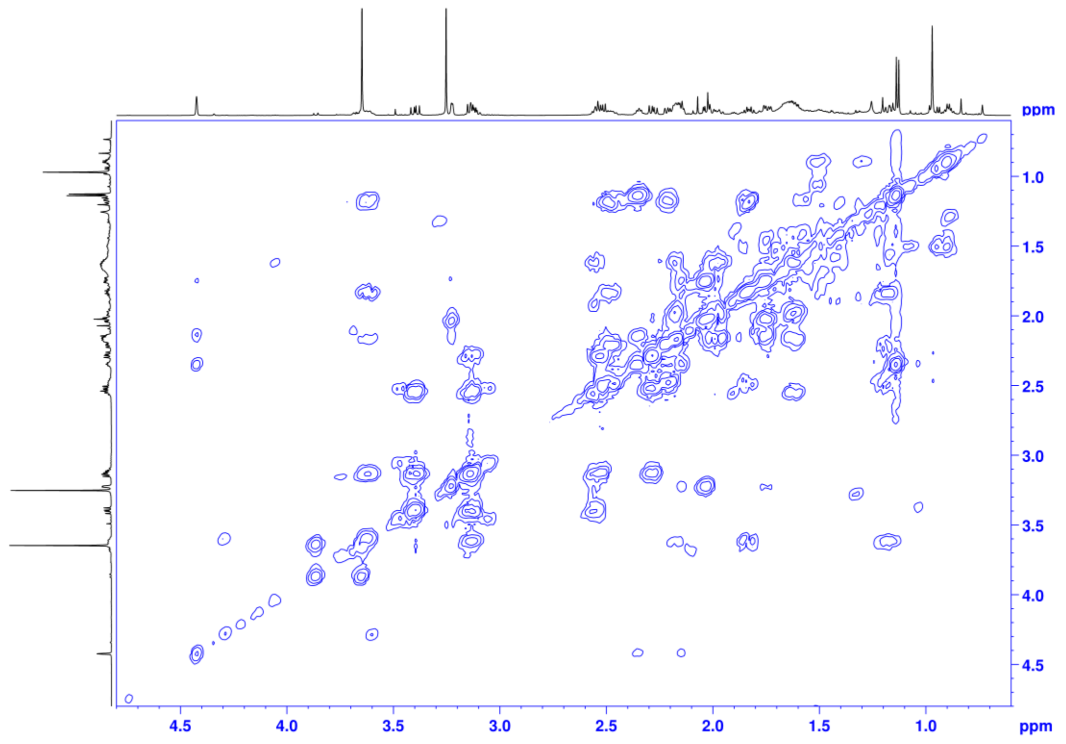

Fig. S25. ^{1}H–^{1}H COSY NMR spectrum for compound **3**

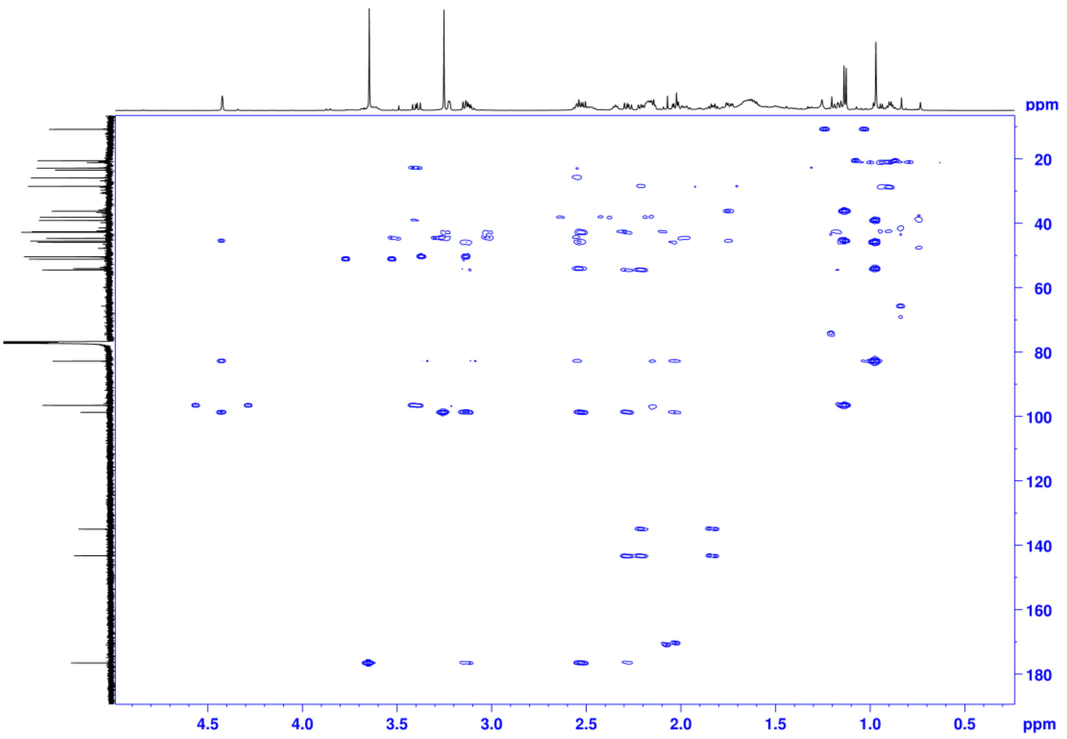

Fig. S26. HMBC NMR spectrum for compound **3**

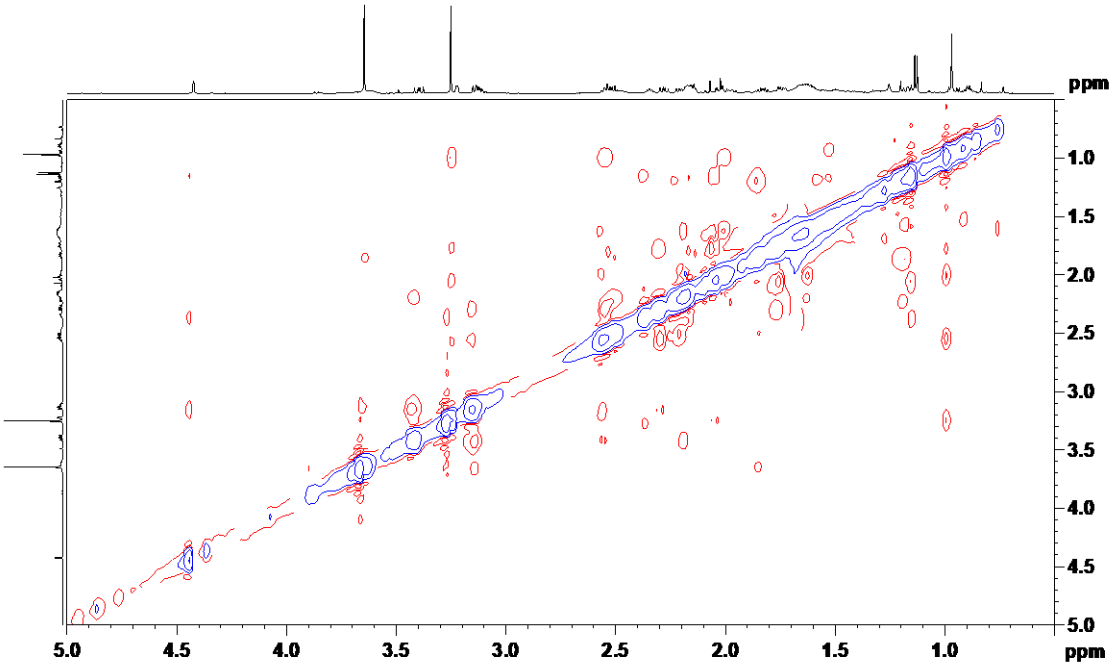

Fig. S27. NOESY NMR spectrum for compound **3**

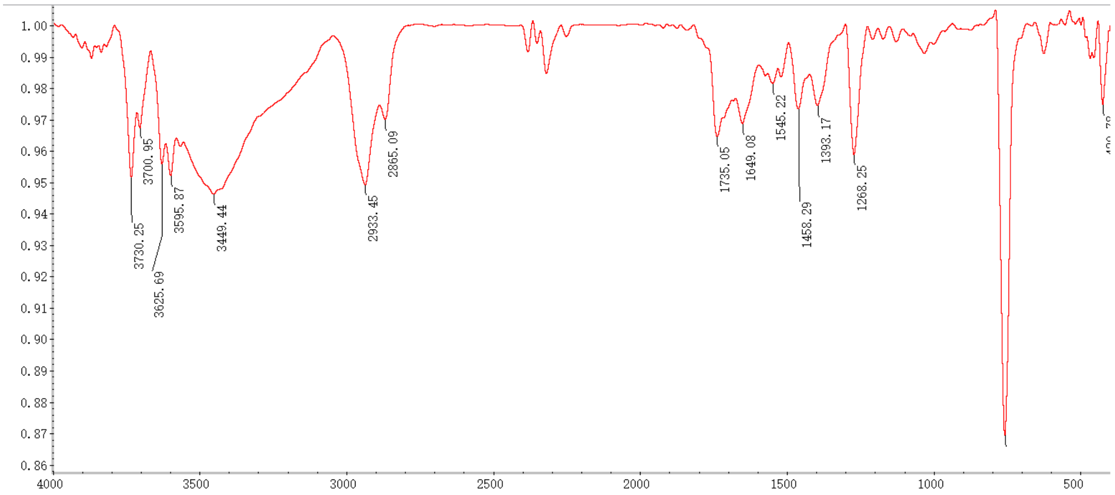

Fig. S28. IR spectrum of compound **3** (KBr)

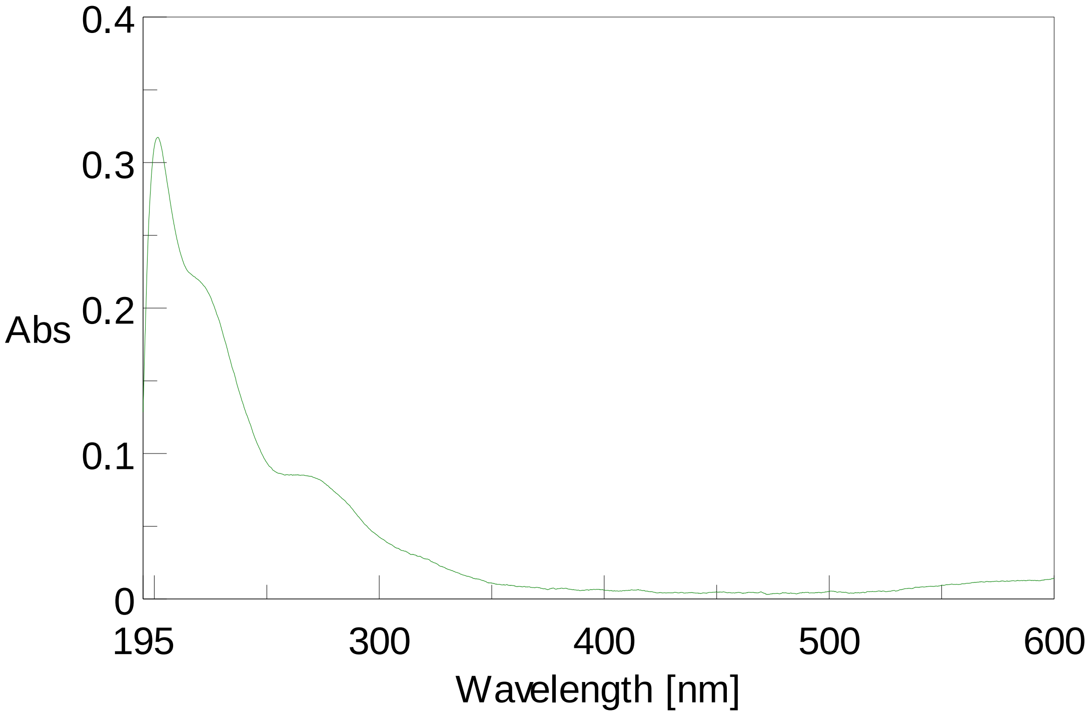

Fig. S29. UV spectrum of compound **3** in MeOH

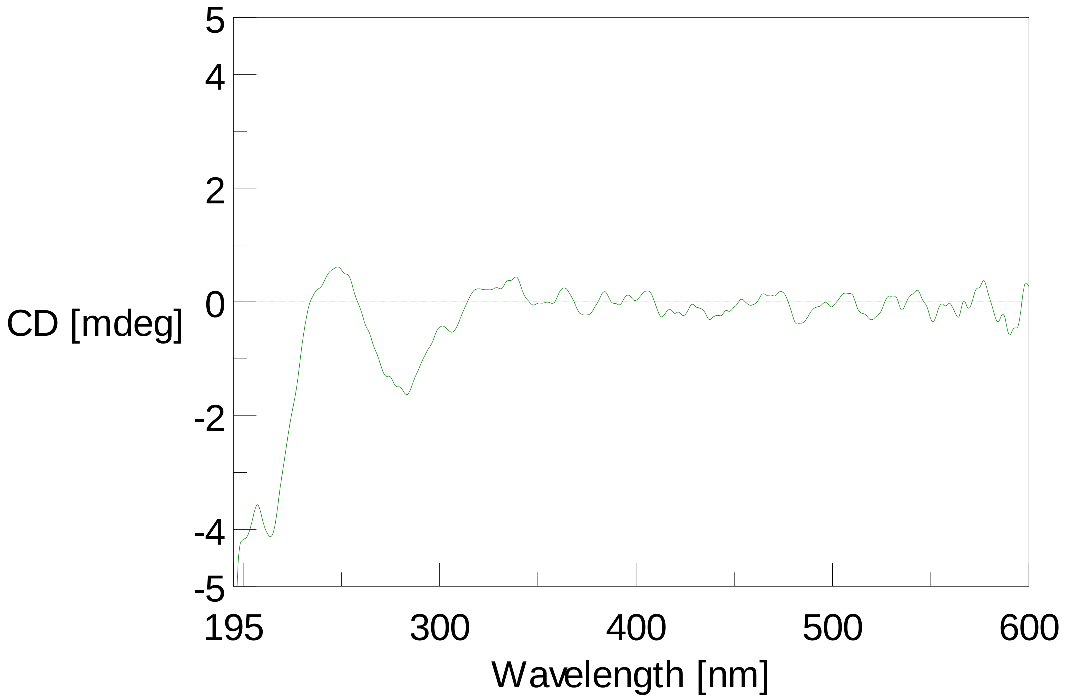

Fig. S30. CD spectrum of compound **3** in MeOH

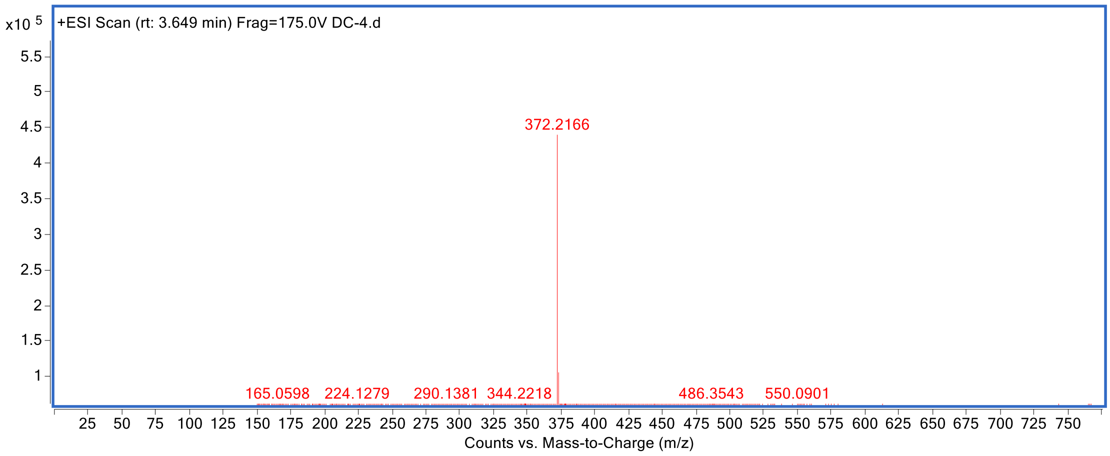

Formula (M)	Score (MFG)	Mass	Mass (MFG)	*m/z* (Calc)	Diff (ppm)	*m/z*
$C_{22}H_{29}NO_4$	99.68	371.2093	371.2097	372.2169	0.9	372.2166

Fig. S31. HR-ESI-MS spectrum of compound **4**

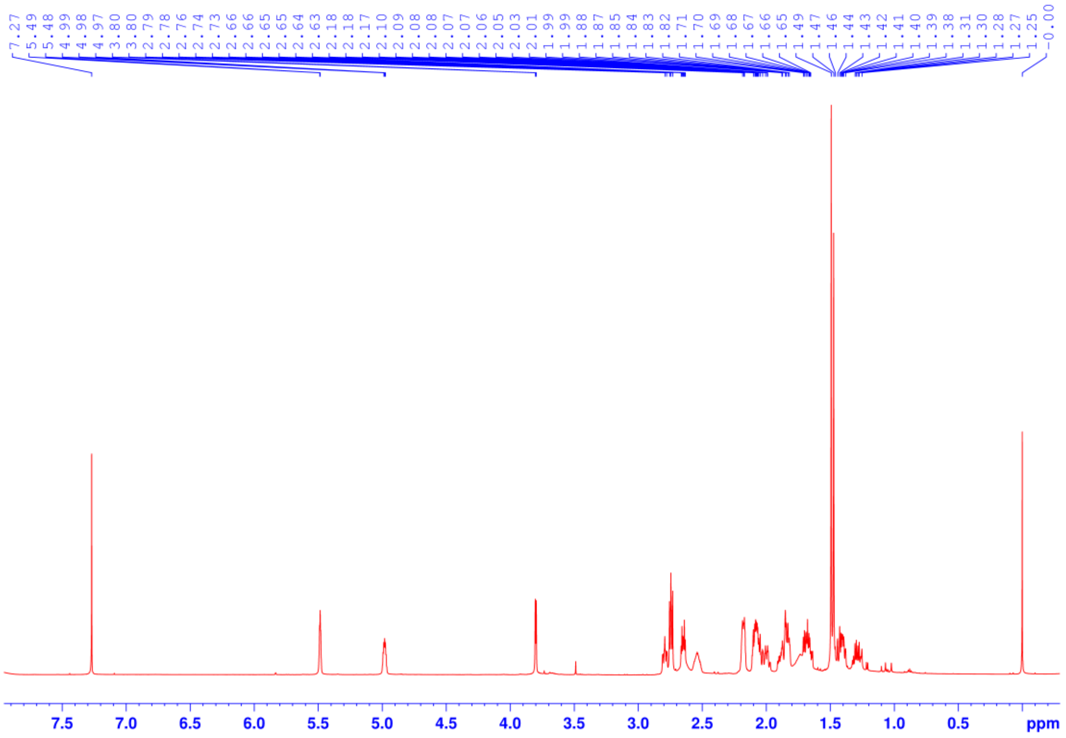

Fig. S32. ^{1}H NMR spectrum for compound **4**

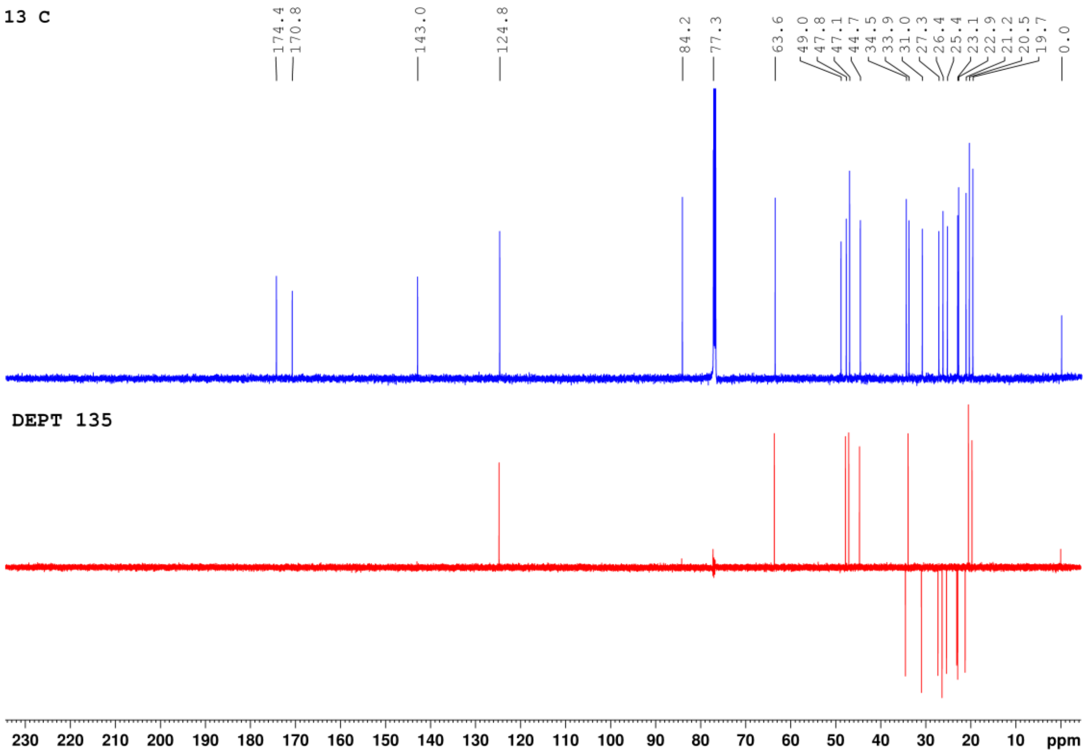

Fig. S33. ^{13}C and DEPT135 NMR spectra for compound **4**

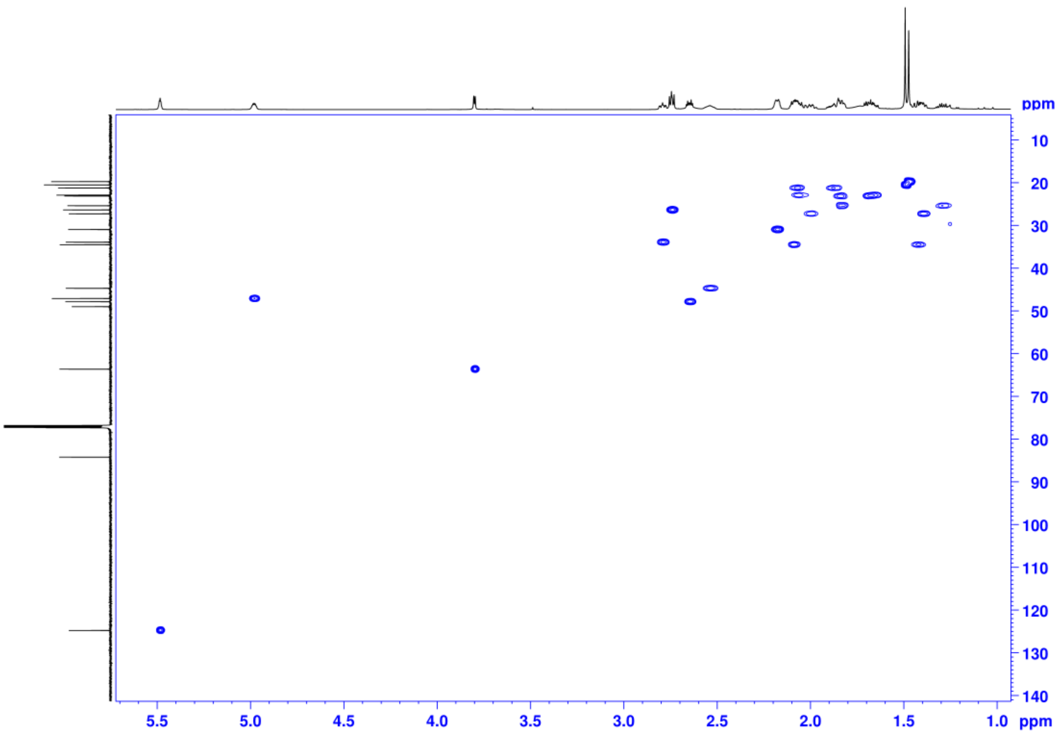

Fig. S34. HSQC NMR spectrum for compound **4**

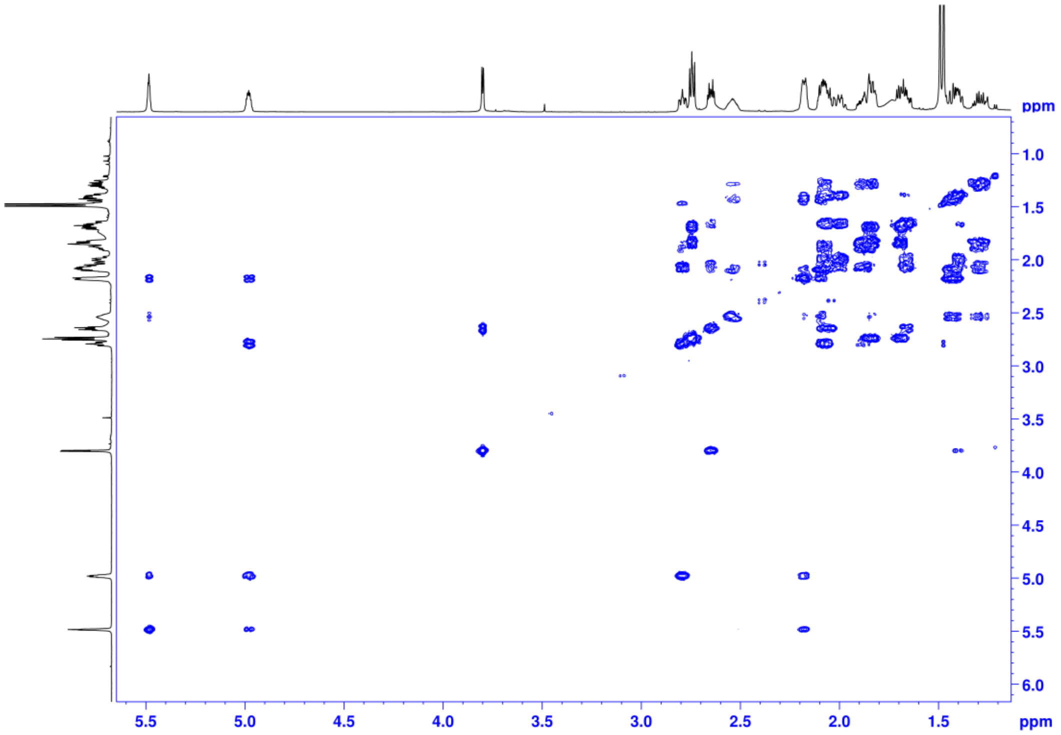

Fig. S35. ^{1}H–^{1}H COSY NMR spectrum for compound **4**

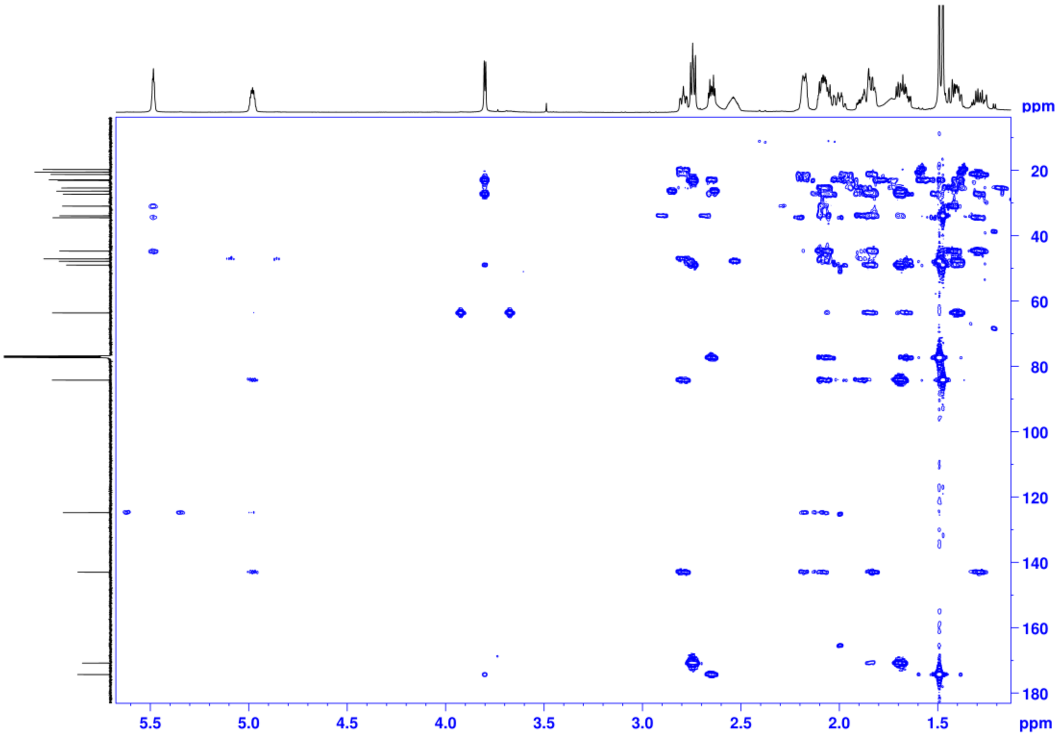

Fig. S36. HMBC NMR spectrum for compound **4**

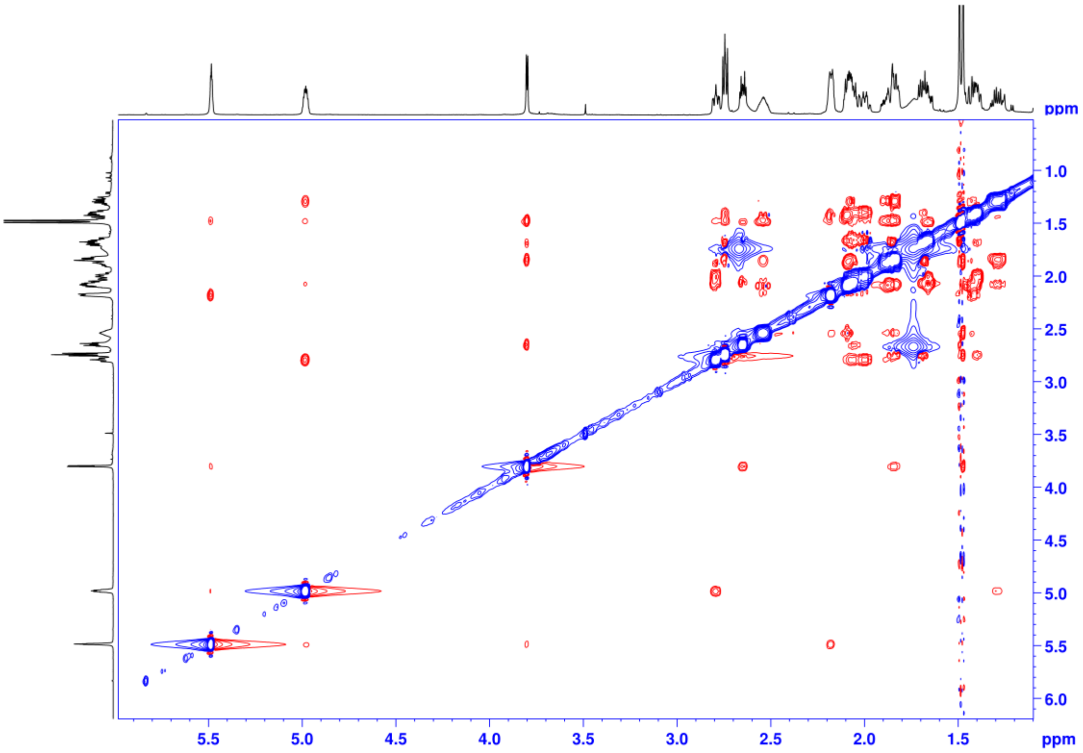

Fig. S37. NOESY NMR spectrum for compound **4**

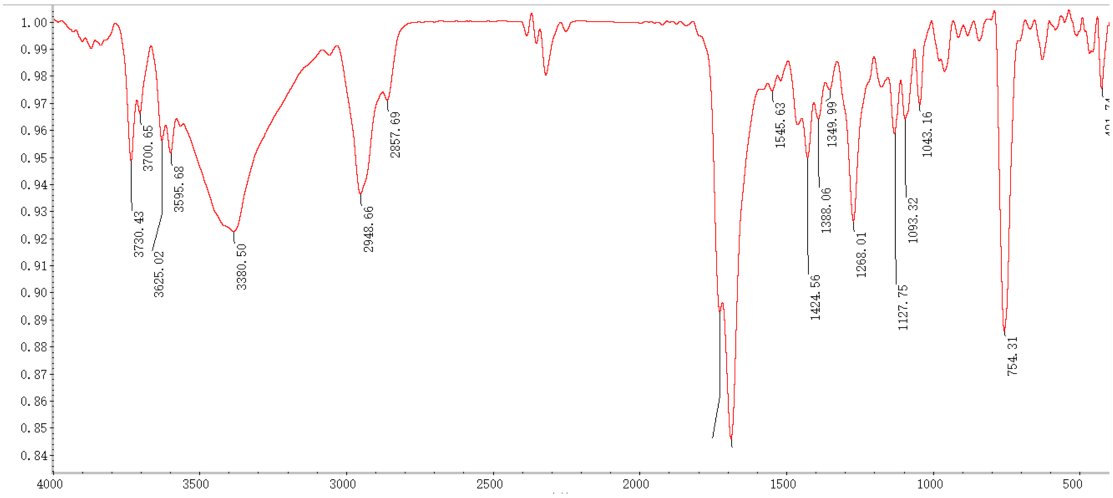

Fig. S38. IR spectrum of compound **4** (KBr)

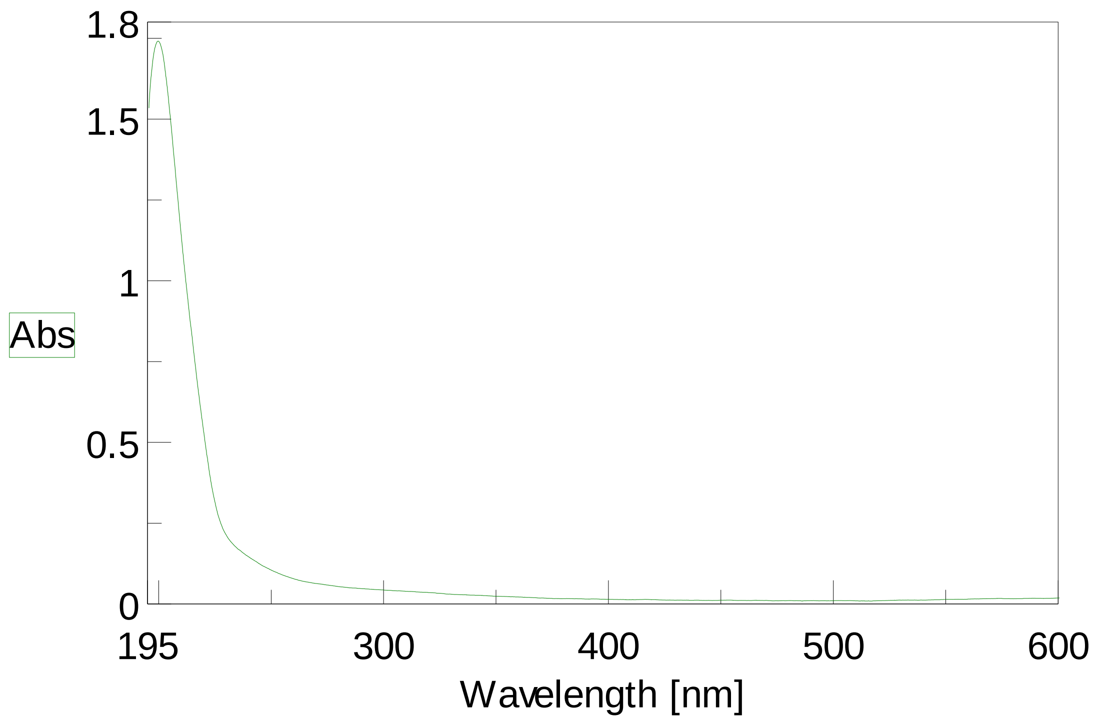

Fig. S39. UV spectrum of compound **4** in MeOH

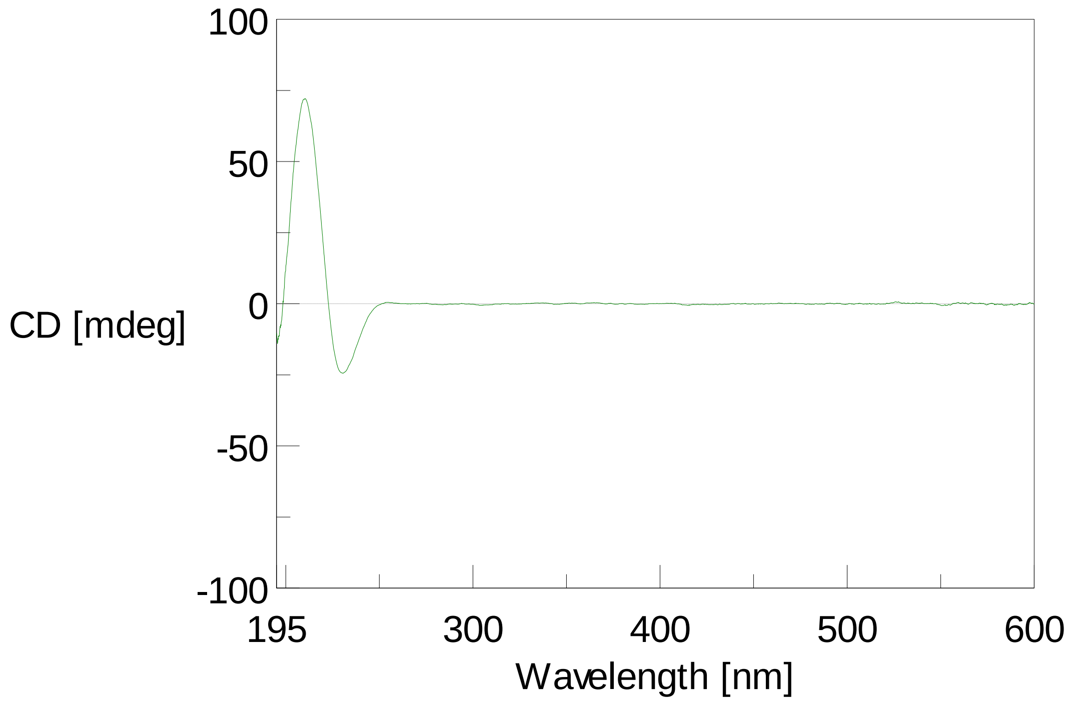

Fig. S40. CD spectrum of compound **4** in MeOH

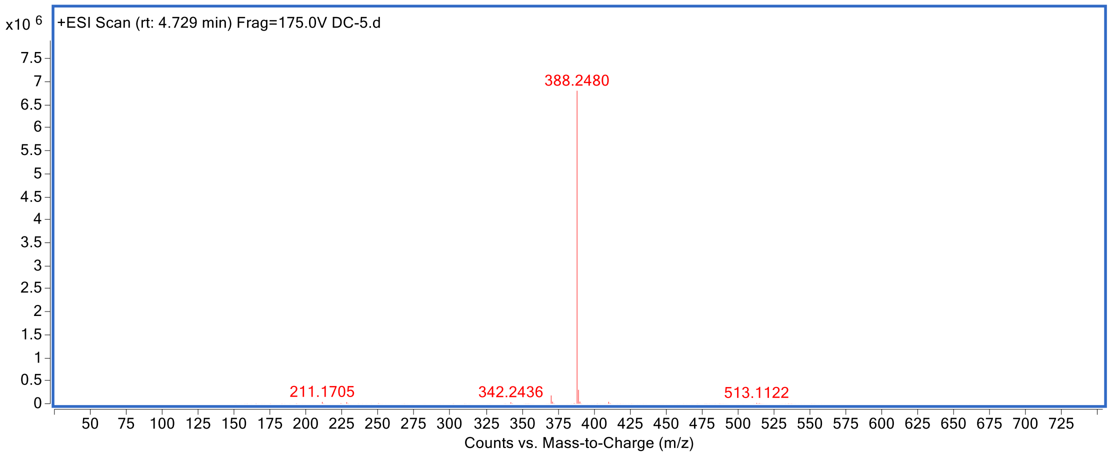

Formula (M)	Score (MFG)	Mass	Mass (MFG)	*m/z* (Calc)	Diff (ppm)	*m/z*
$C_{23}H_{33}NO_4$	99.85	387.2407	387.241	388.2482	0.61	388.248

Fig. S41. HR-ESI-MS spectrum of compound **5**

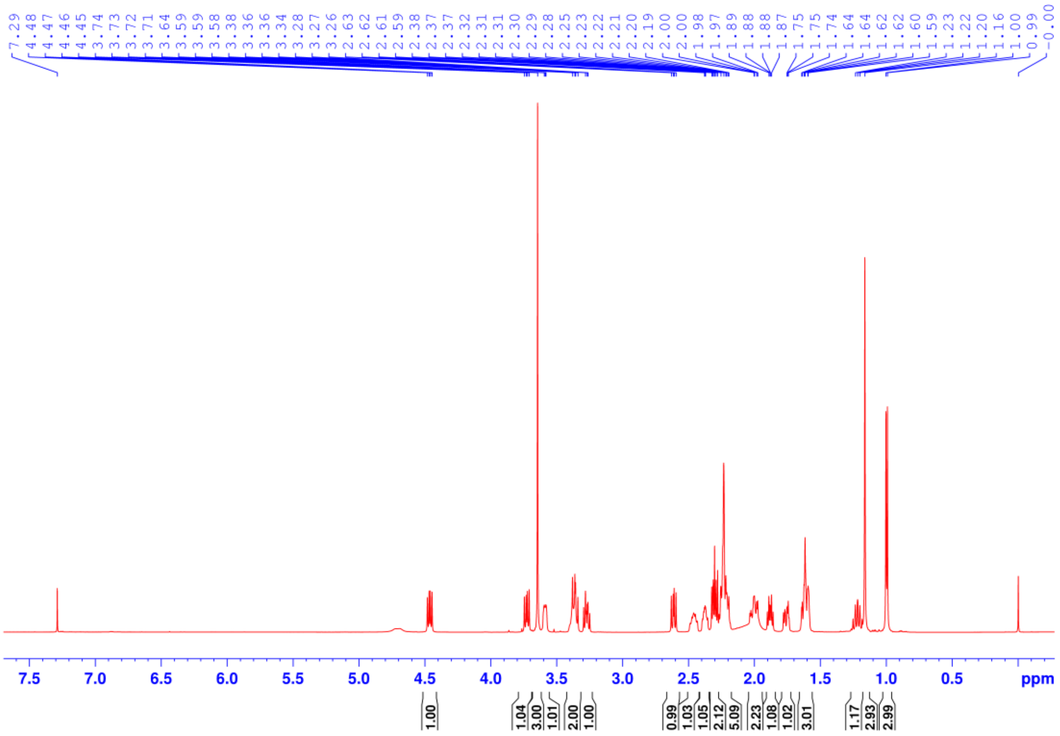

Fig. S42. ^{1}H NMR spectrum for compound **5**

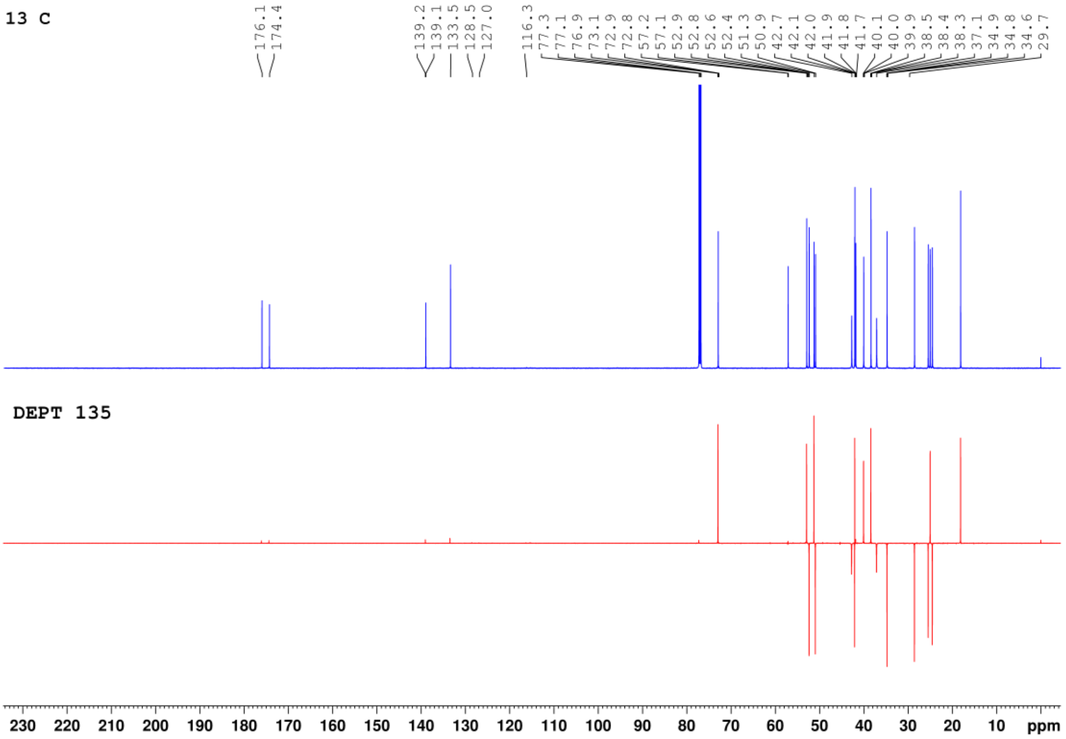

Fig. S43. ^{13}C and DEPT135 NMR spectra for compound **5**

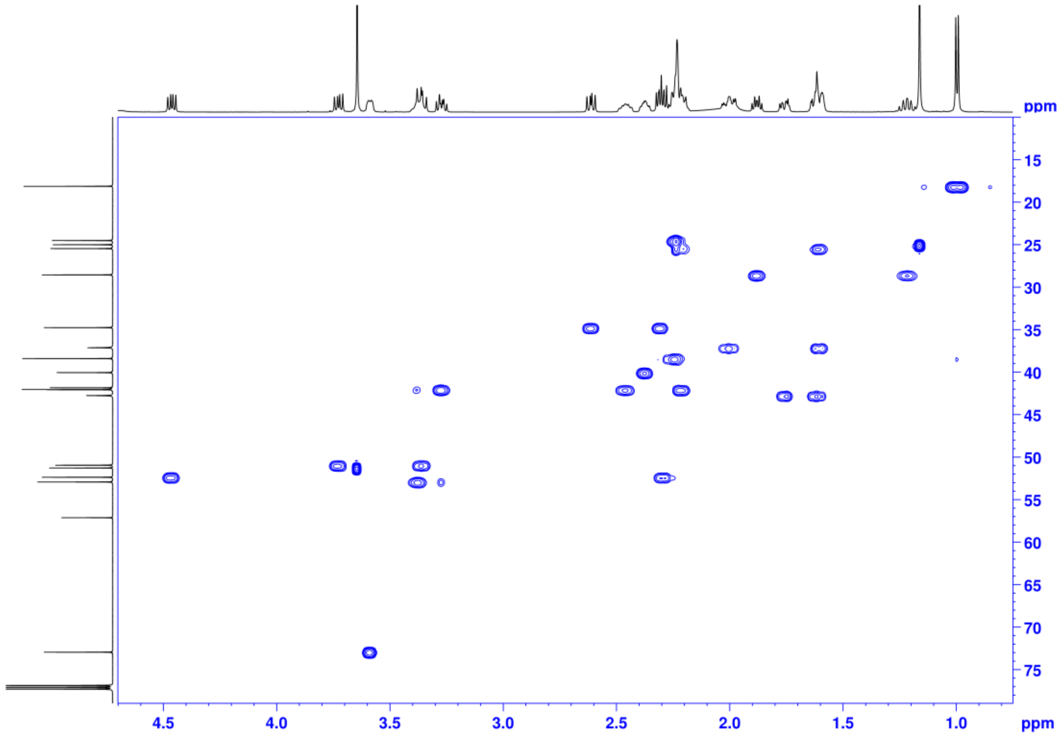

Fig. S44. HSQC NMR spectrum for compound **5**

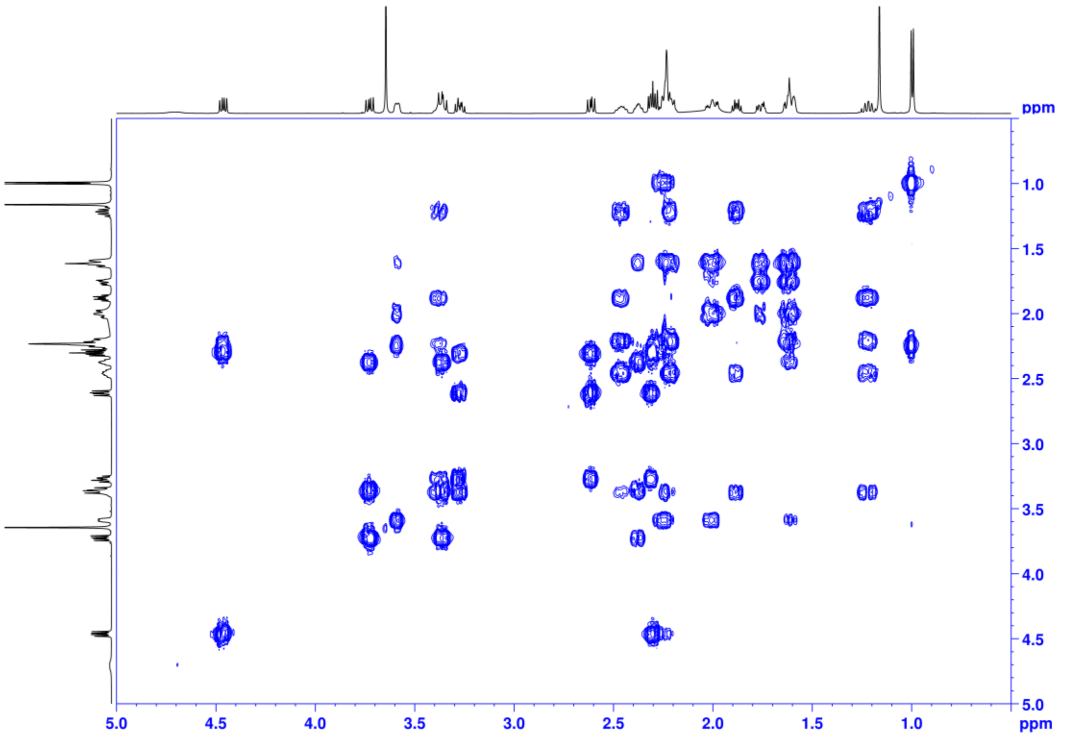

Fig. S45. ^{1}H–^{1}H COSY NMR spectrum for compound 5

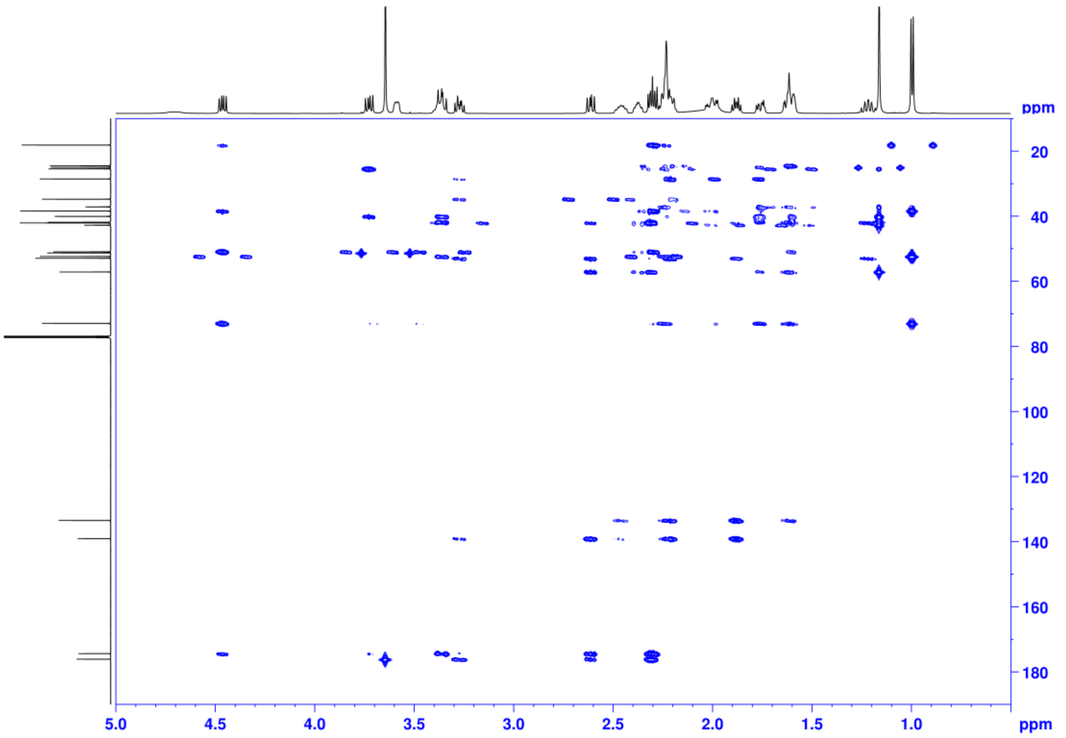

Fig. S46. HMBC NMR spectrum for compound **5**

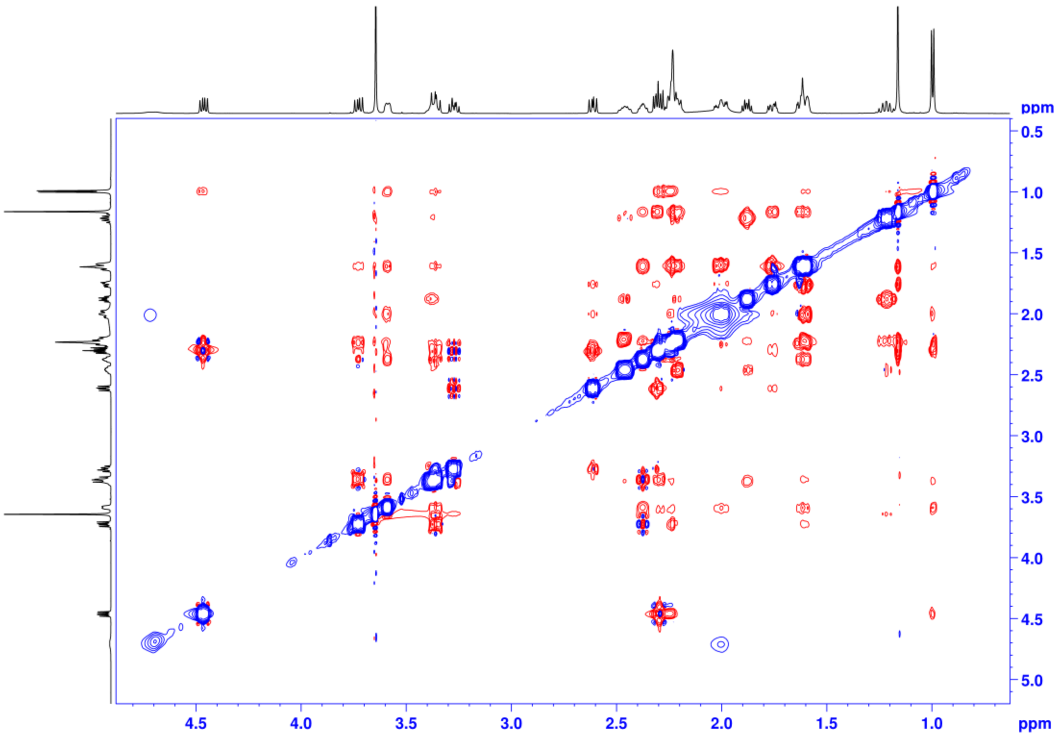

Fig. S47. NOESY NMR spectrum for compound **5**

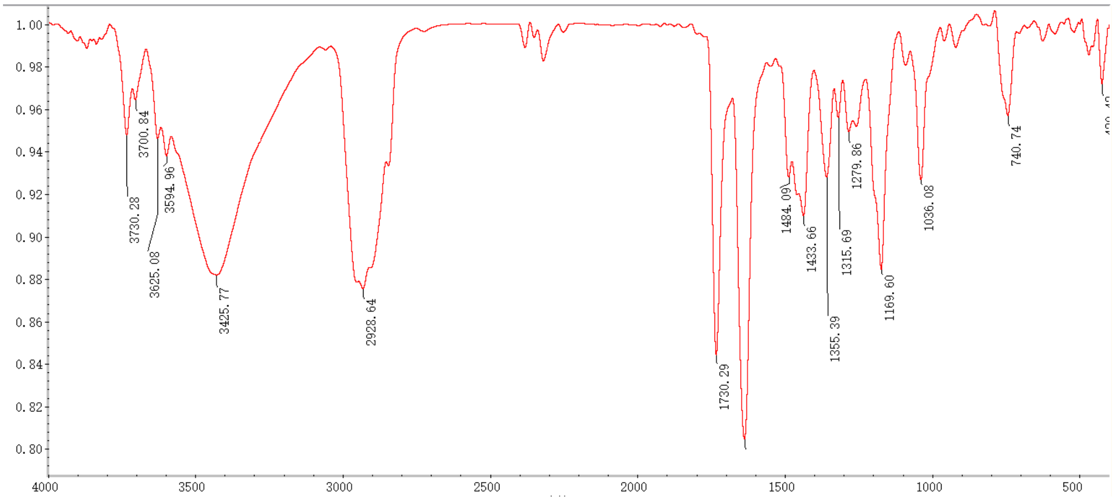

Fig. S48. IR spectrum of compound **5** (KBr)

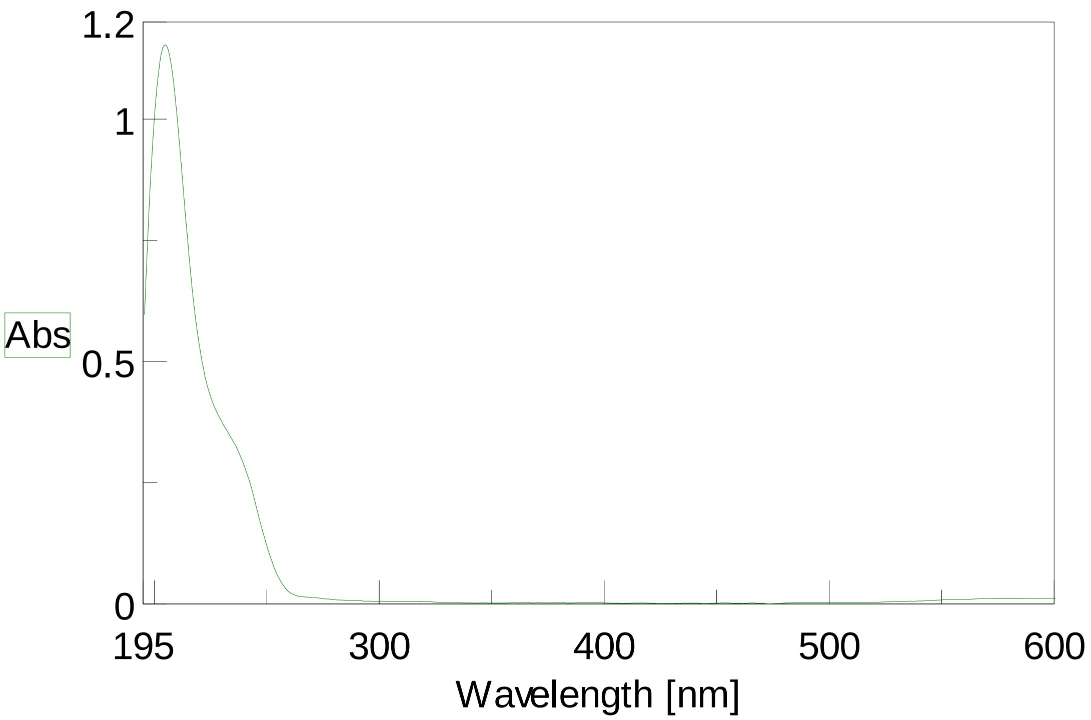

Fig. S49. UV spectrum of compound **5** in MeOH

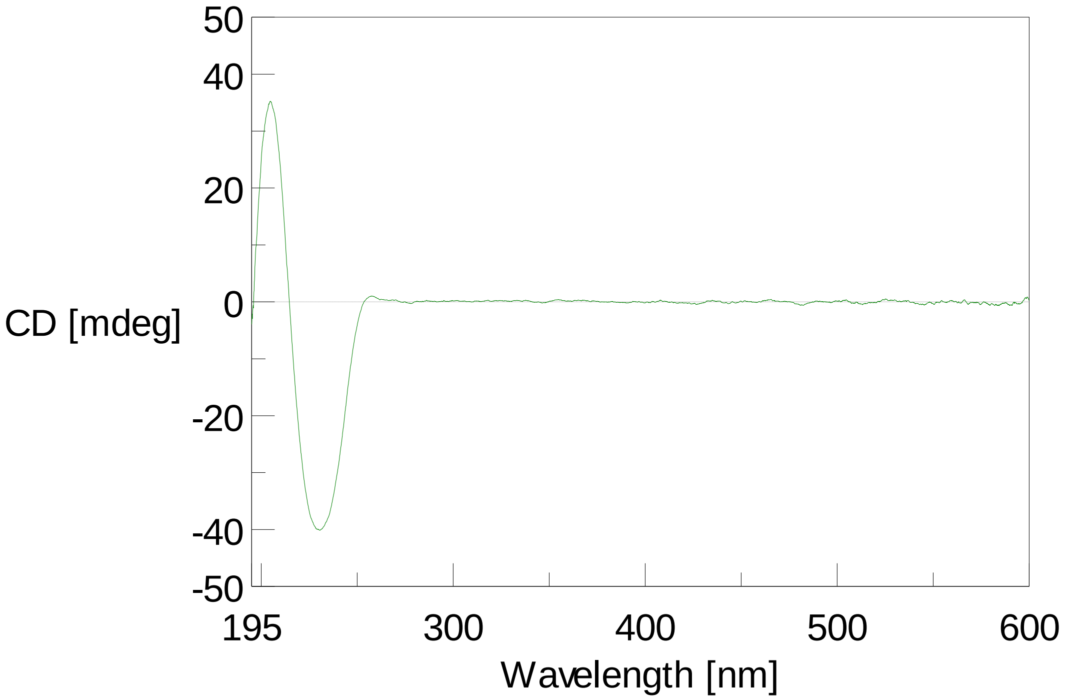

Fig. S50. CD spectrum of compound **5** in MeOH

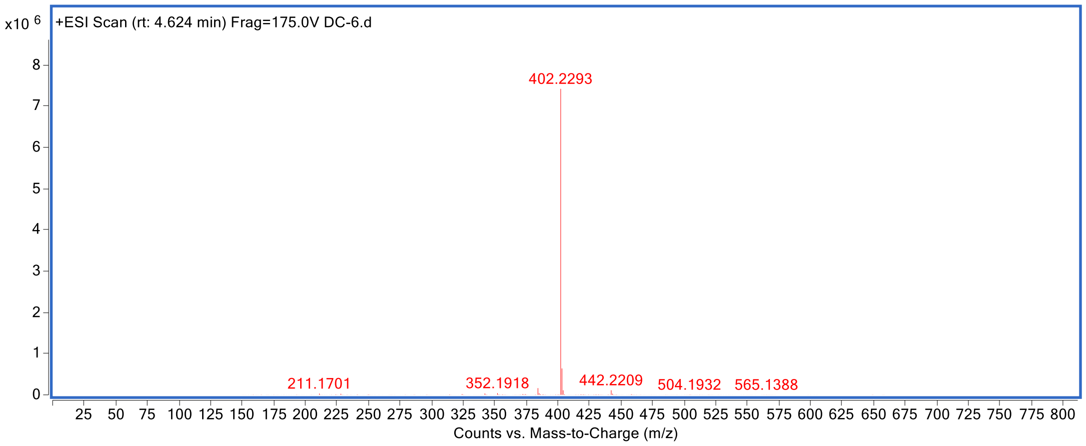

Formula (M)	Score (MFG)	Mass	Mass (MFG)	*m/z* (Calc)	Diff (ppm)	*m/z*
$C_{23}H_{31}NO_5$	92.17	401.222	401.2202	402.2275	-4.49	402.2293

Fig. S51. HR-ESI-MS spectrum of compound 6

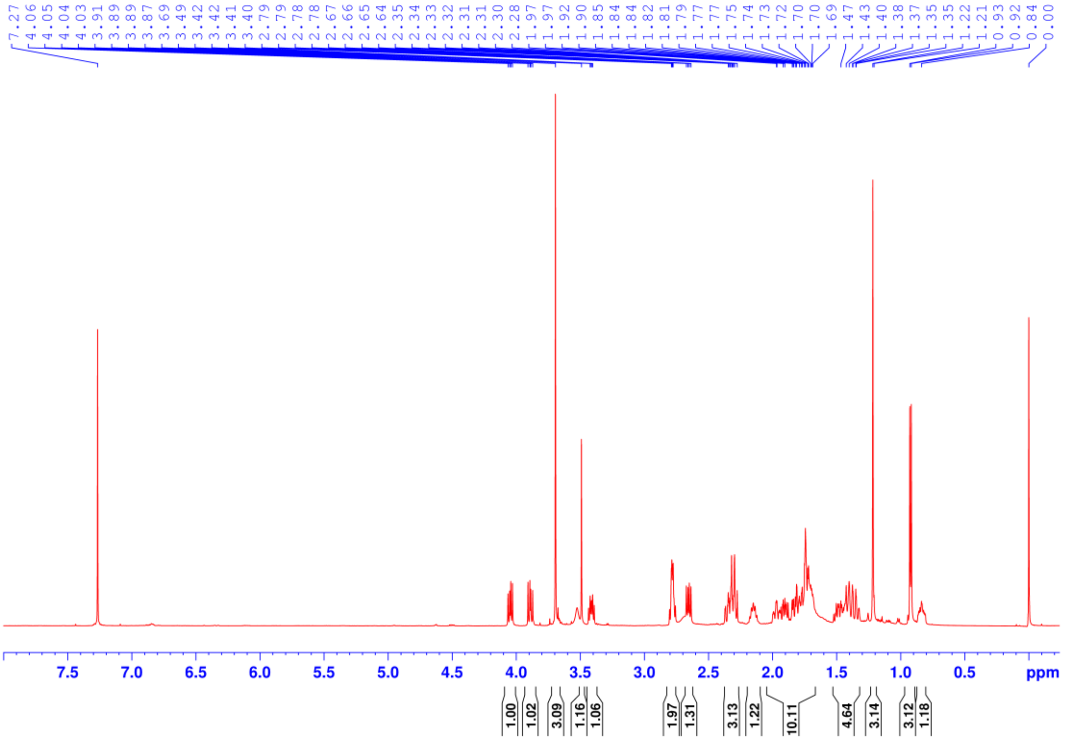

Fig. S52. ^{1}H NMR spectrum for compound **6**

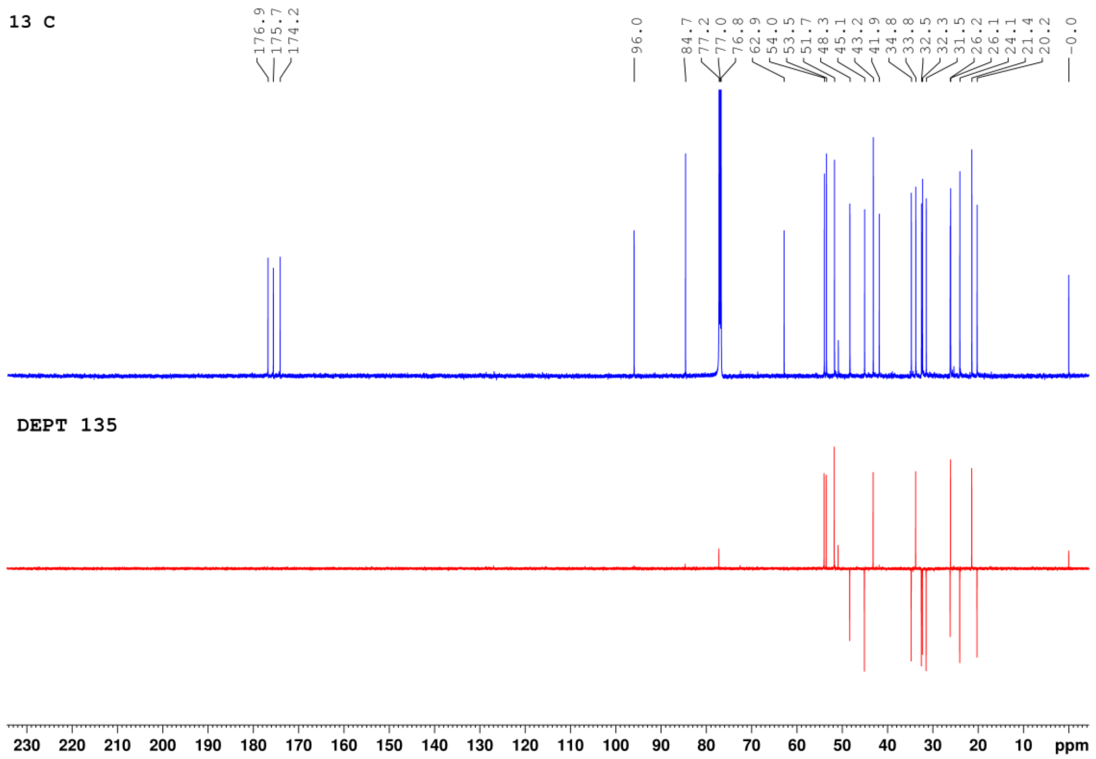

Fig. S53. ^{13}C and DEPT135 NMR spectra for compound **6**

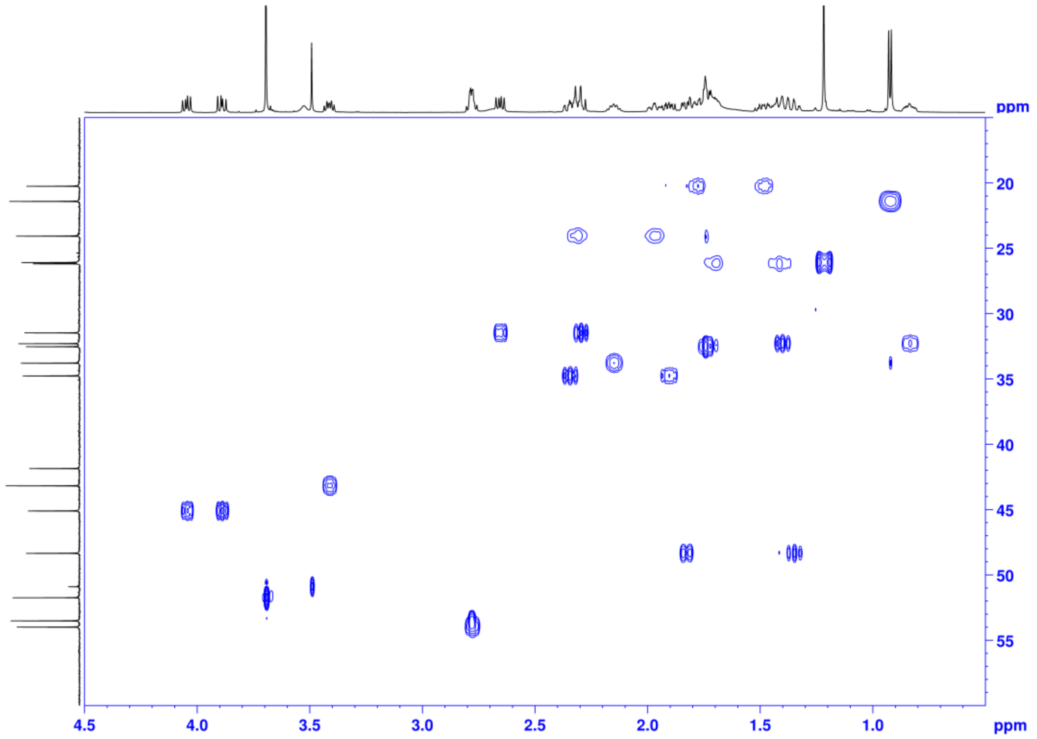

Fig. S54. HSQC NMR spectrum for compound **6**

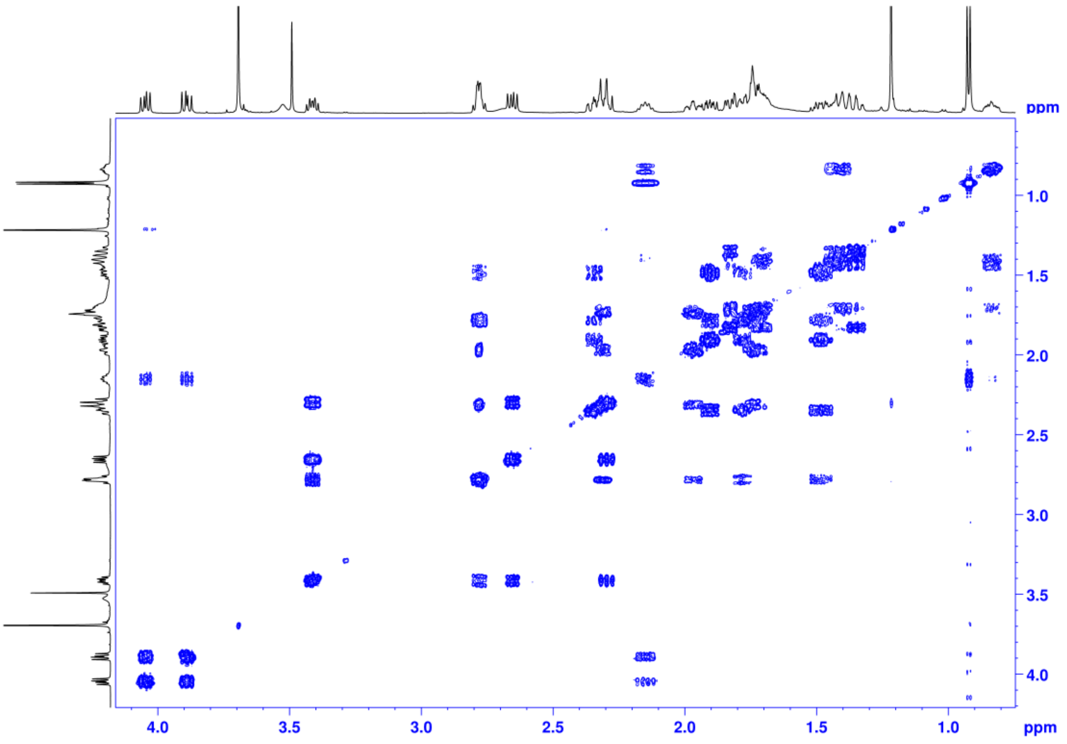

Fig. S55. ^{1}H–^{1}H COSY NMR spectrum for compound **6**

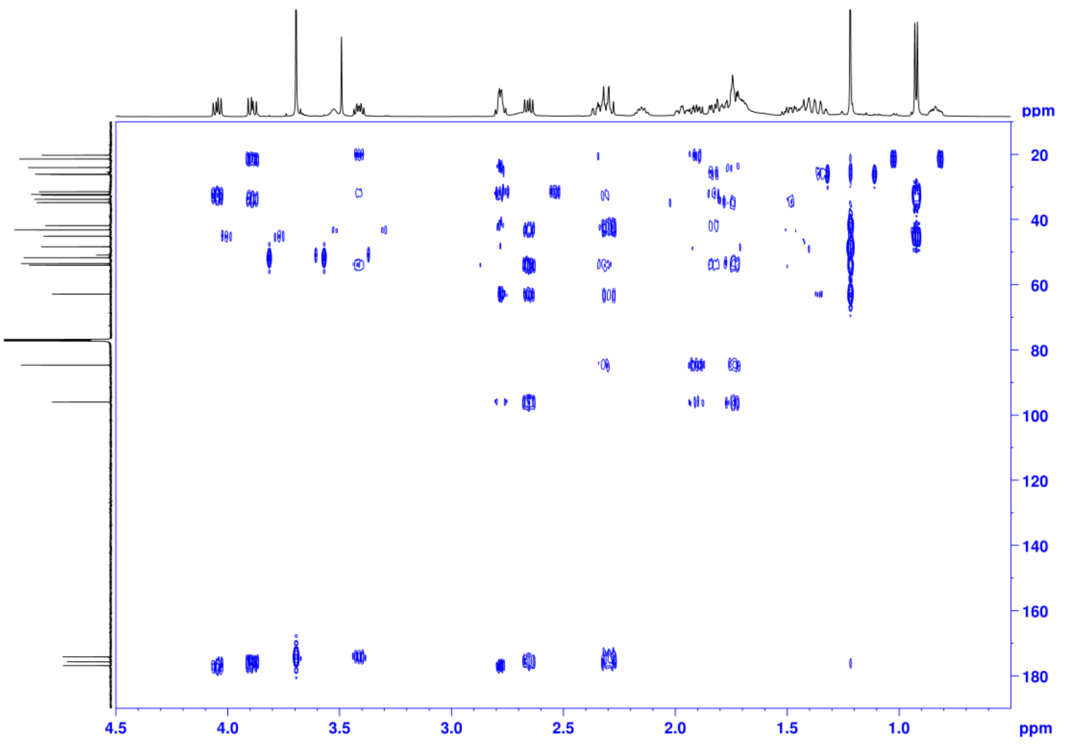

Fig. S56. HMBC NMR spectrum for compound **6**

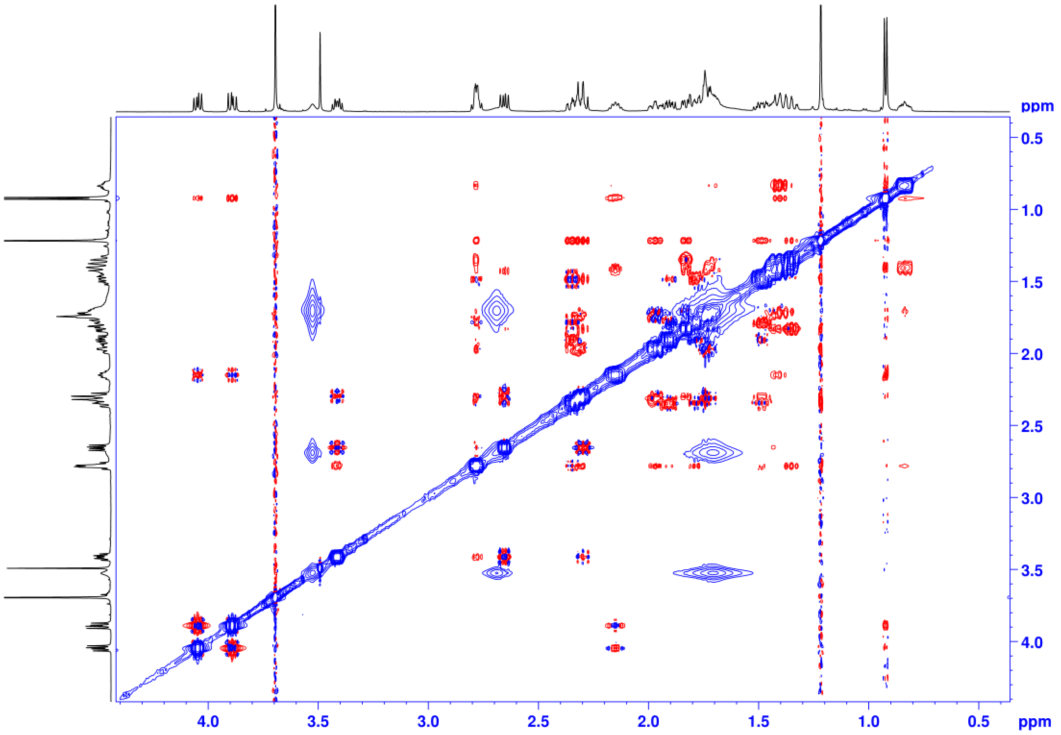

Fig. S57. NOESY NMR spectrum for compound 6

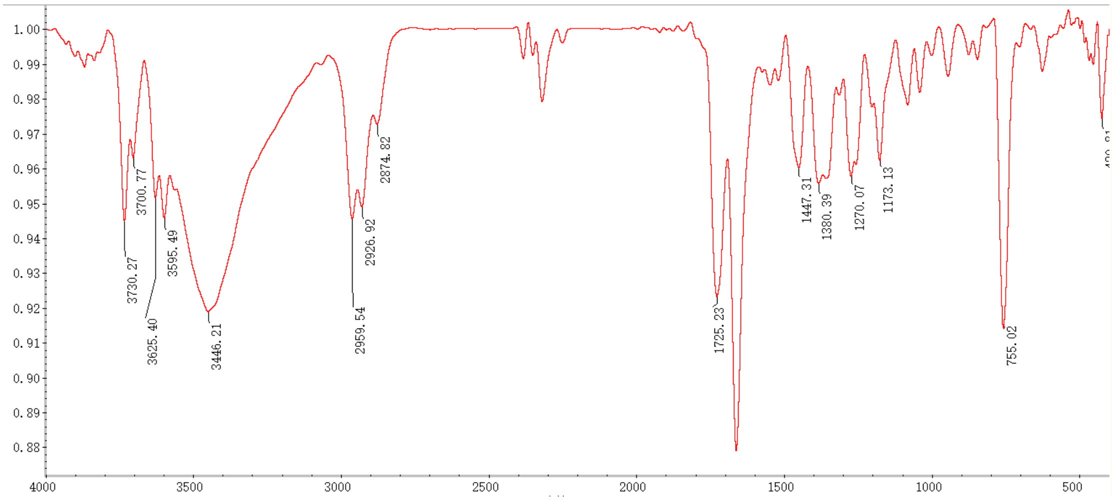

Fig. S58. IR spectrum of compound **6** (KBr)

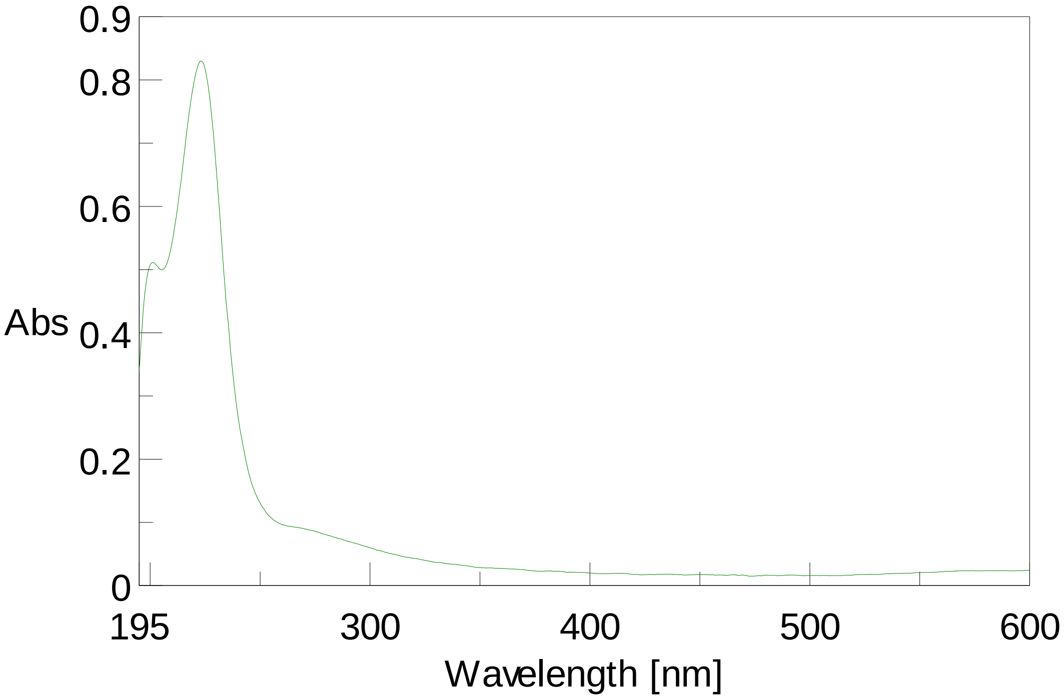

Fig. S59. UV spectrum of compound **6** in MeOH

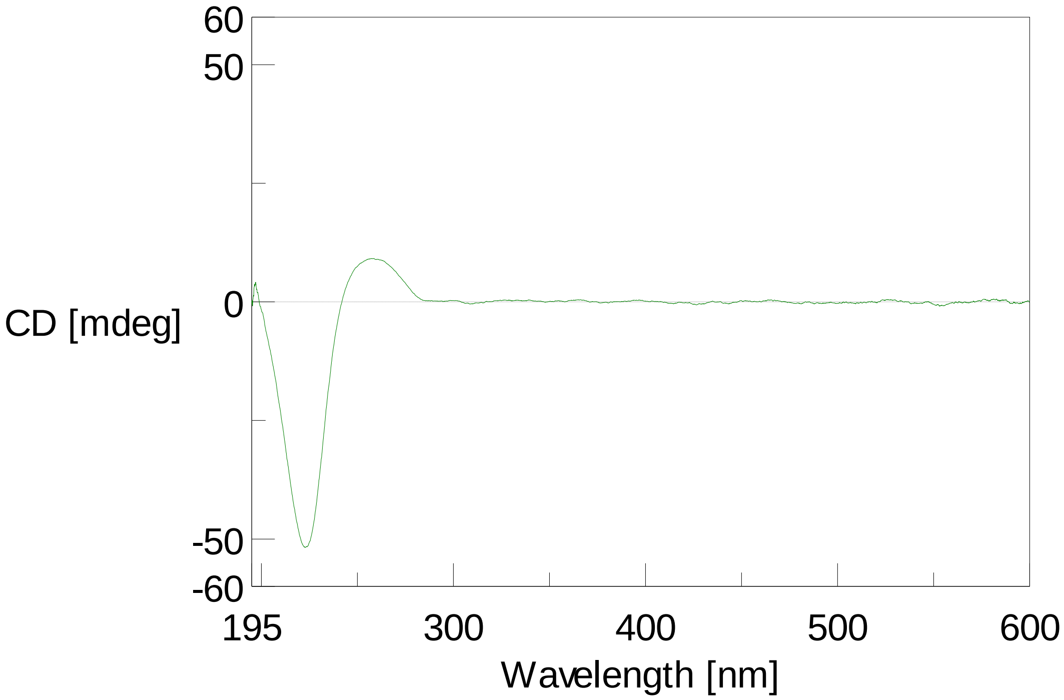

Fig. S60. CD spectrum of compound **6** in MeOH

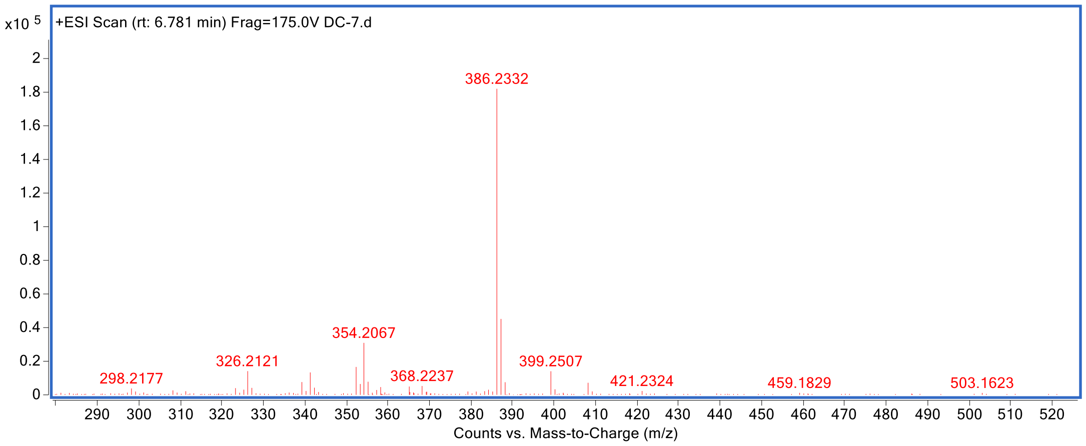

Formula (M)	Score (MFG)	Mass	Mass (MFG)	*m/z* (Calc)	Diff (ppm)	*m/z*
$C_{23}H_{31}NO_4$	98.97	385.2259	385.2253	386.2326	-1.6	386.2332

Fig. S61. HR-ESI-MS spectrum of compound 7

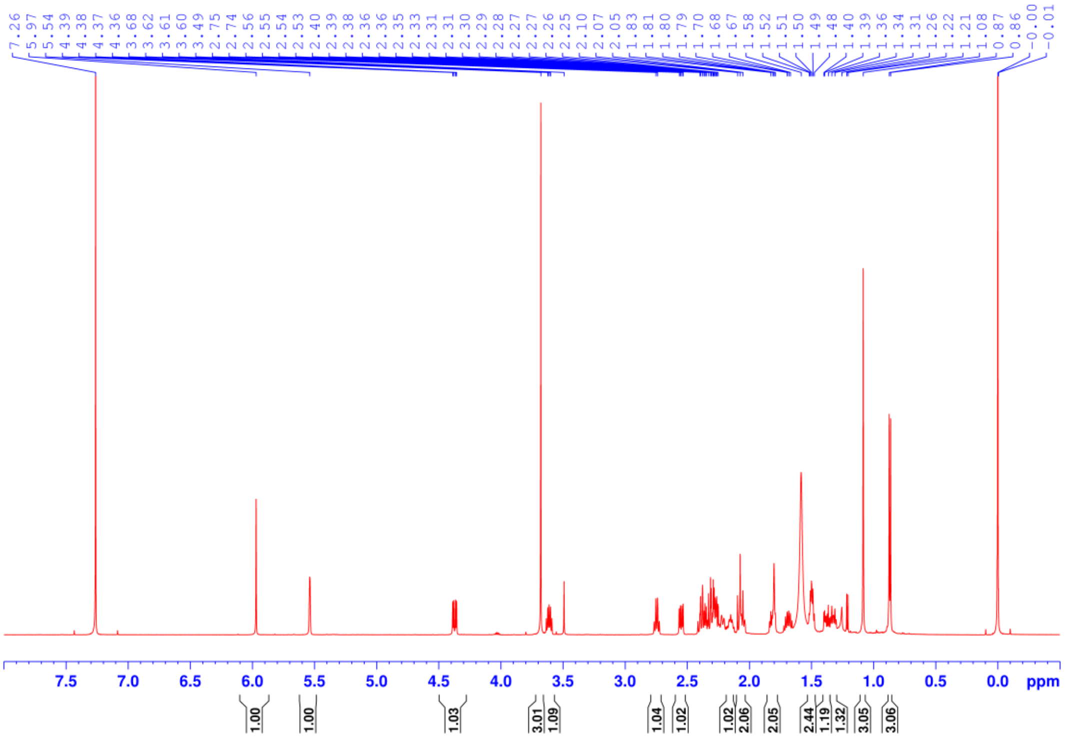

Fig. S62. ^{1}H NMR spectrum for compound **7**

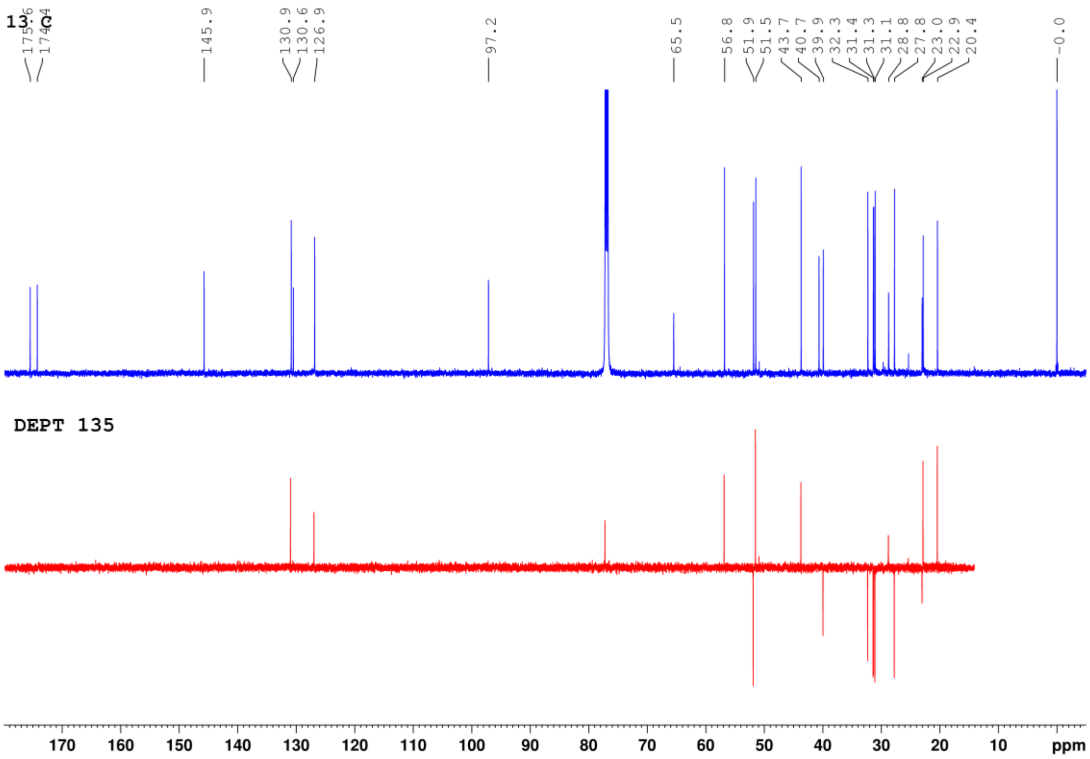

Fig. S63. ^{13}C and DEPT135 NMR spectra for compound **7**

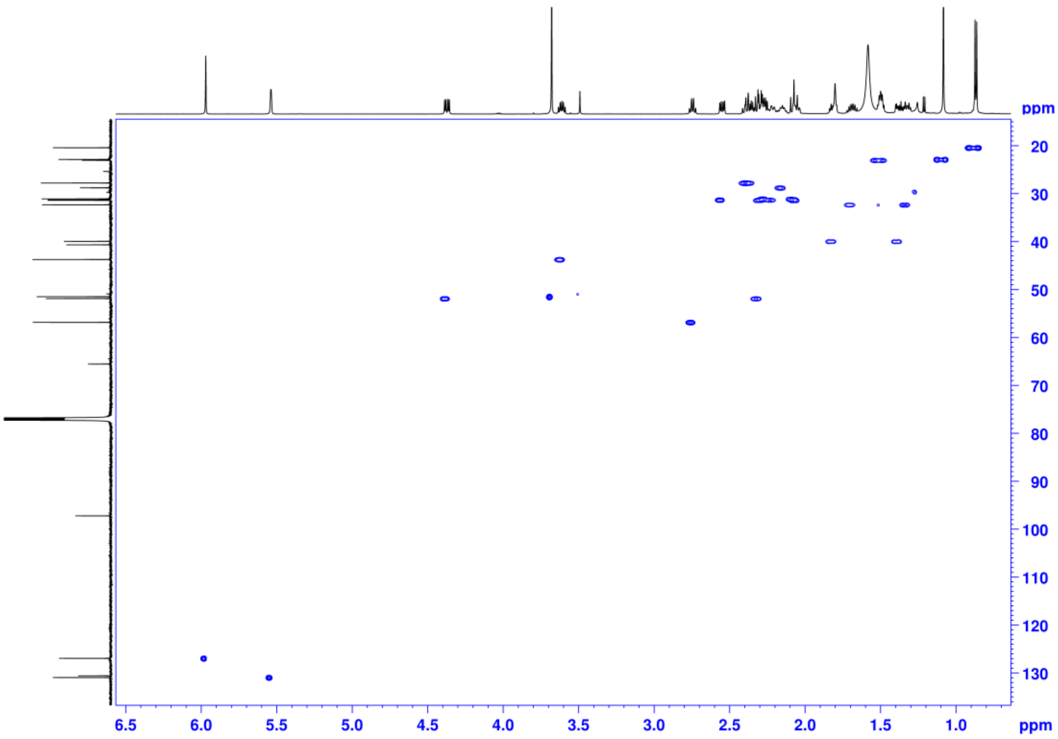

Fig. S64. HSQC NMR spectrum for compound **7**

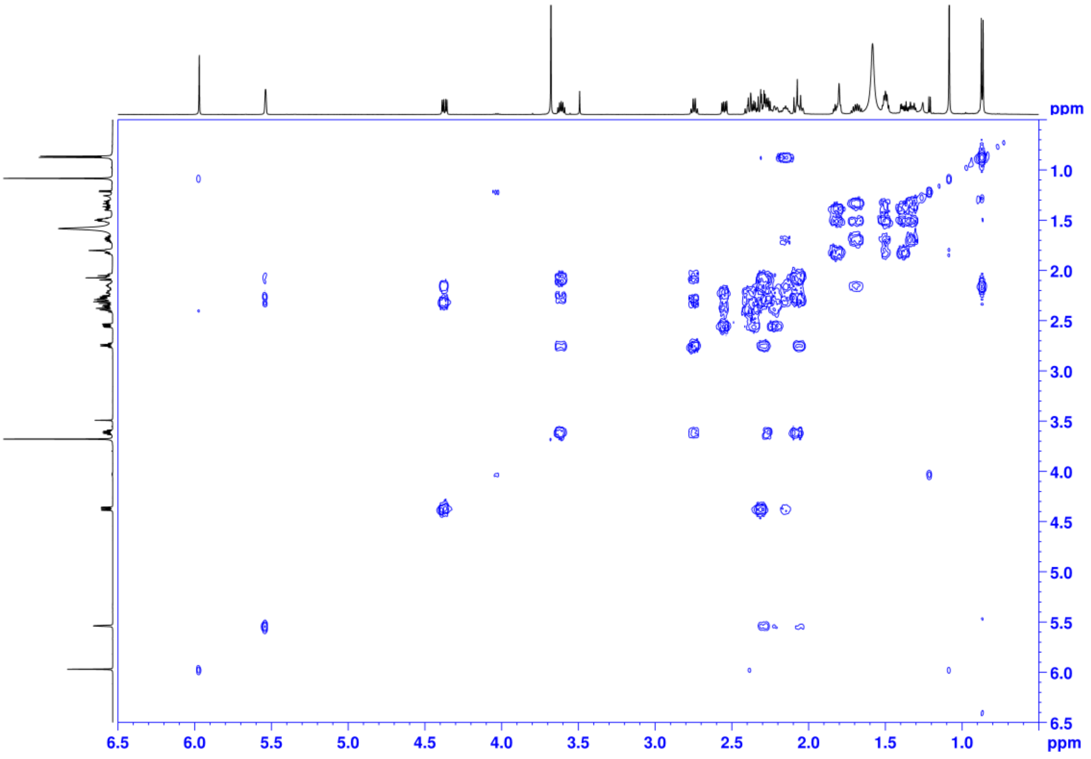

Fig. S65. ^{1}H–^{1}H COSY NMR spectrum for compound **7**

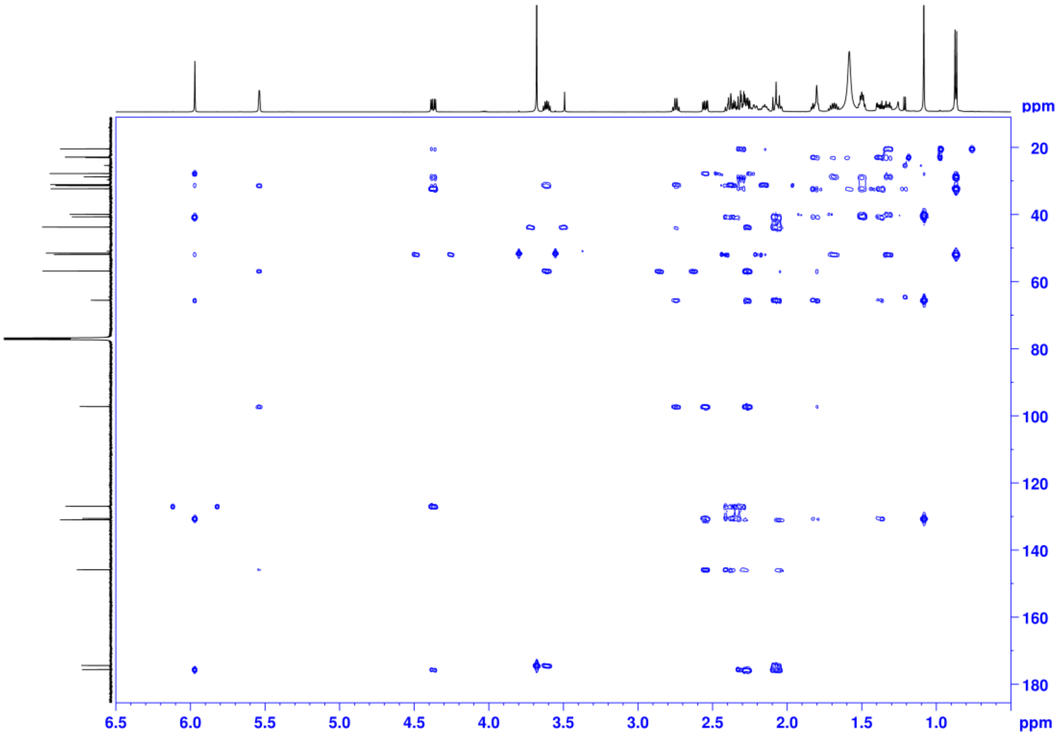

Fig. S66. HMBC NMR spectrum for compound **7**

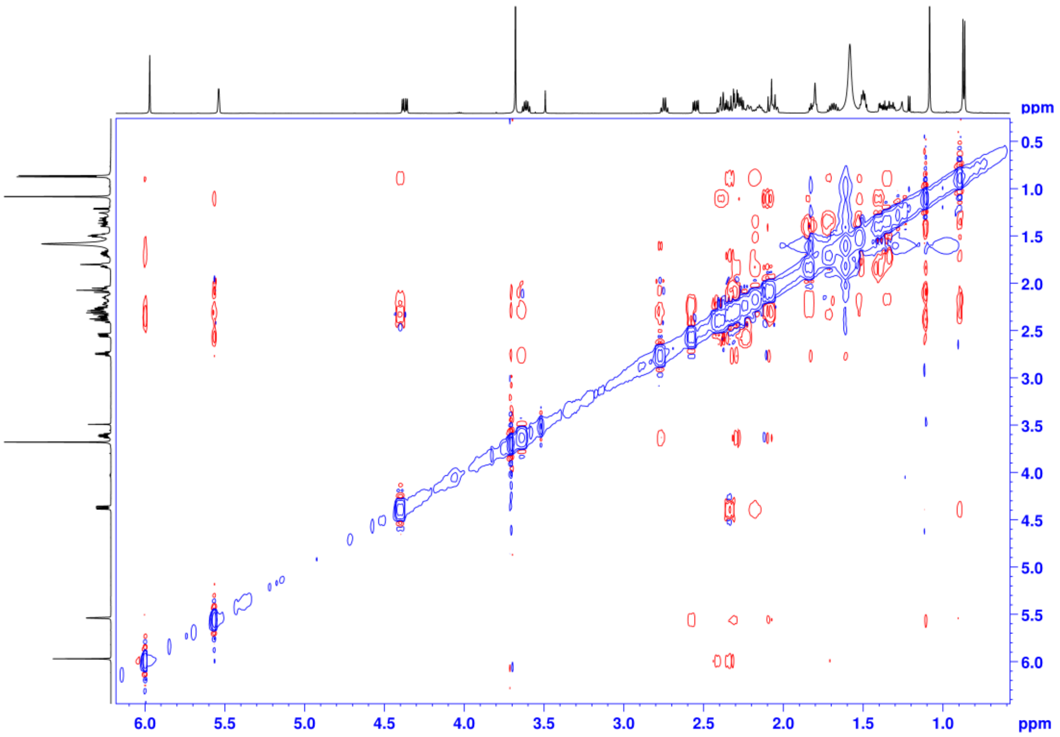

Fig. S67. NOESY NMR spectrum for compound **7**

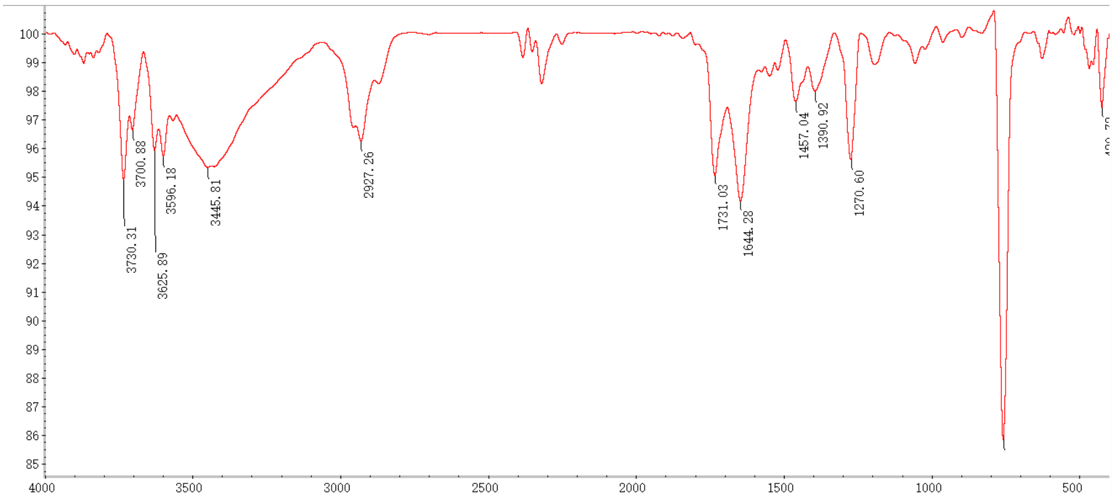

Fig. S68. IR spectrum of compound **7** (KBr)

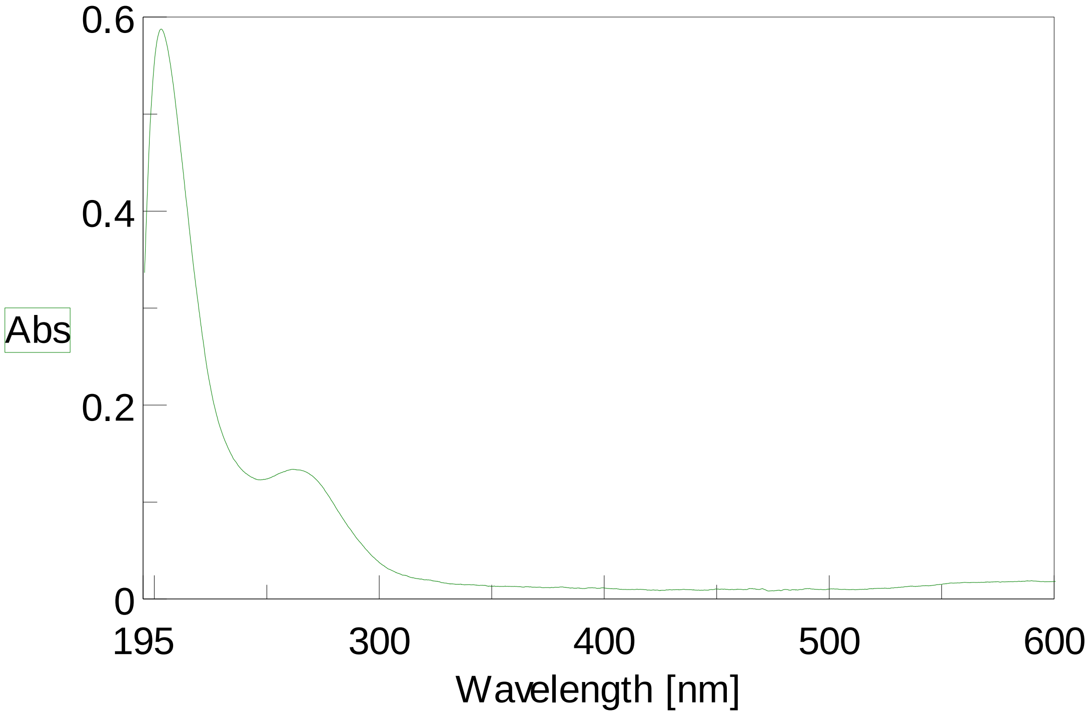

Fig. S69. UV spectrum of compound **7** in MeOH

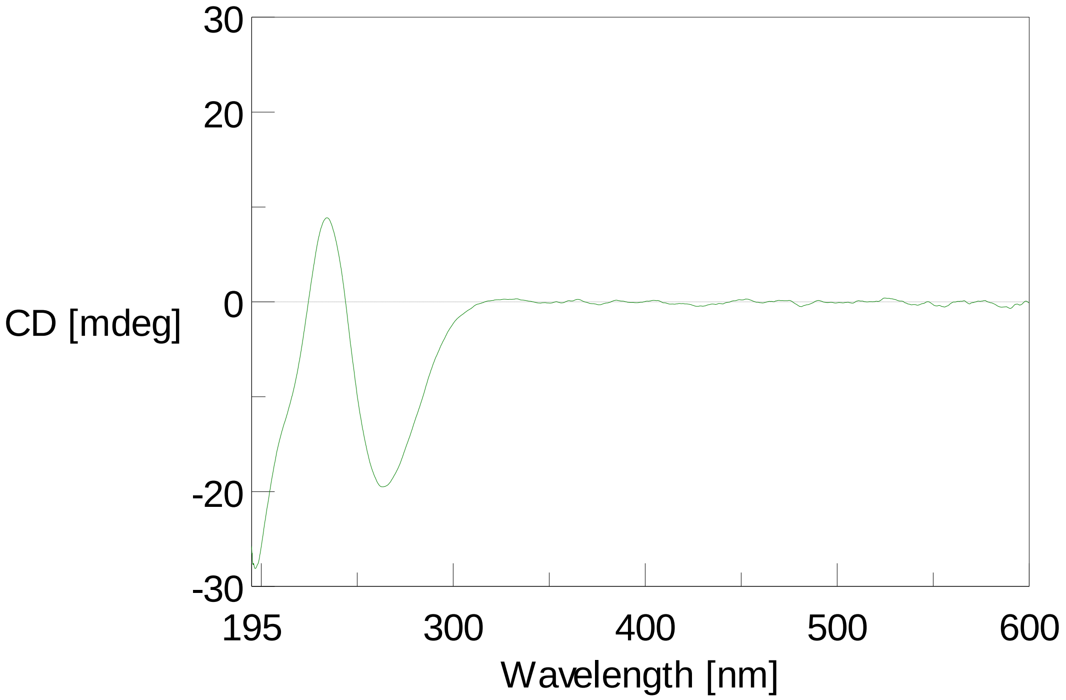

Fig. S70. CD spectrum of compound **7** in MeOH

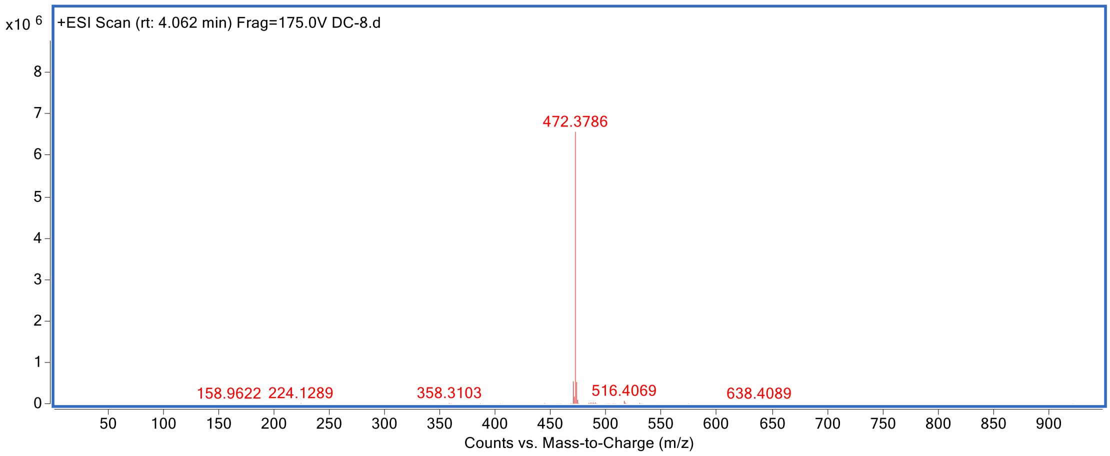

Formula (M)	Score (MFG)	Mass	Mass (MFG)	*m/z* (Calc)	Diff (ppm)	*m/z*
$C_{30}H_{49}NO_3$	100	471.3712	471.3712	472.3785	0.04	472.3785

Fig. S71. HR-ESI-MS spectrum of compound **8**

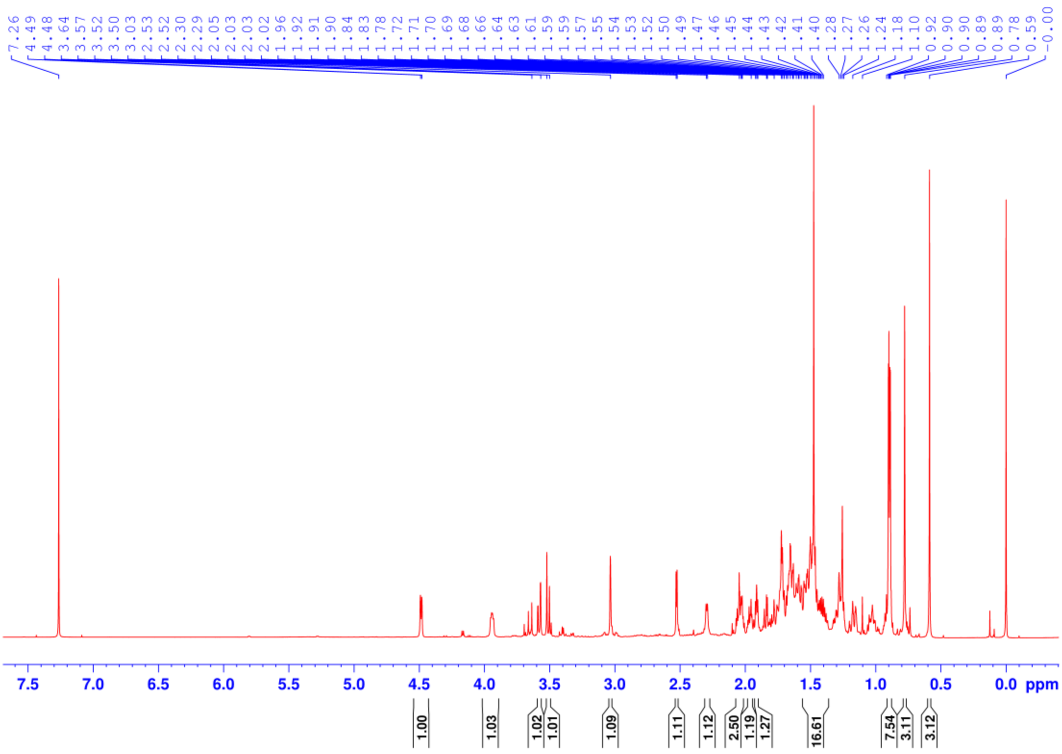

Fig. S72. ^{1}H NMR spectrum for compound **8**

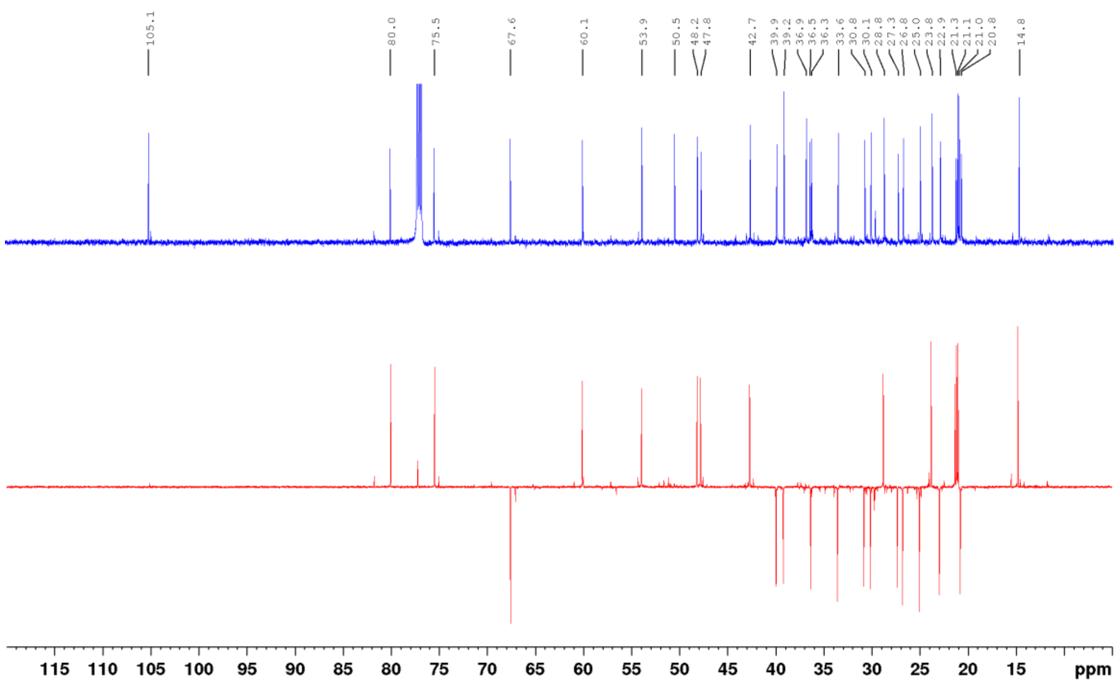

Fig. S73. ^{13}C and DEPT135 NMR spectra for compound **8**

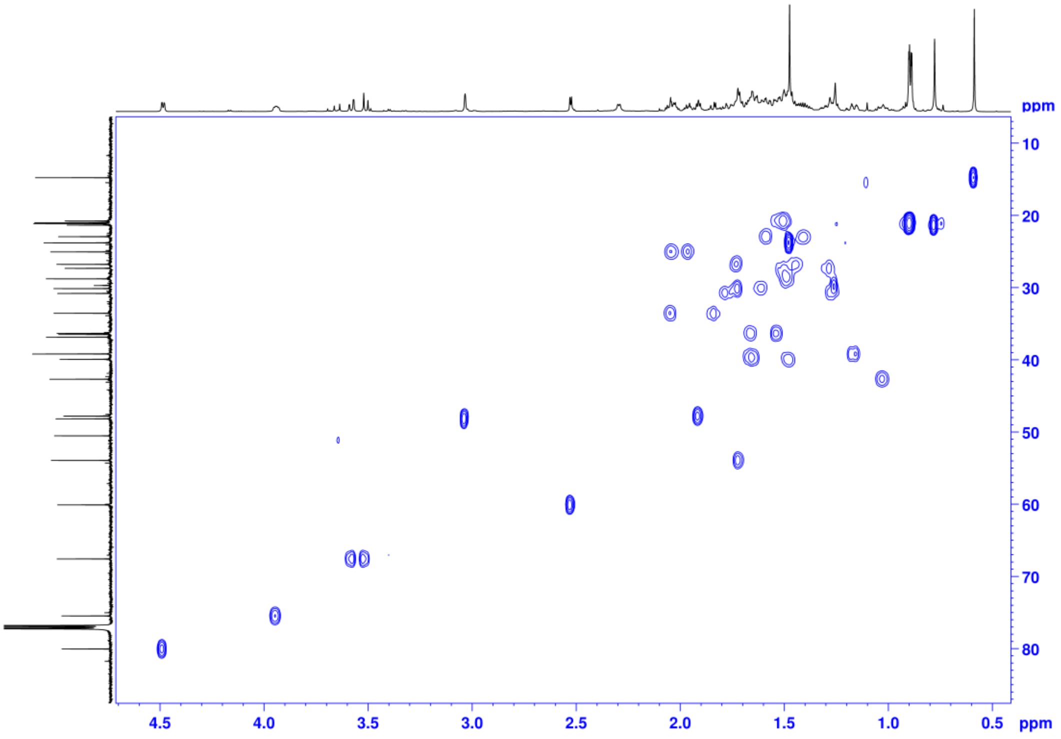

Fig. S74. HSQC NMR spectrum for compound **8**

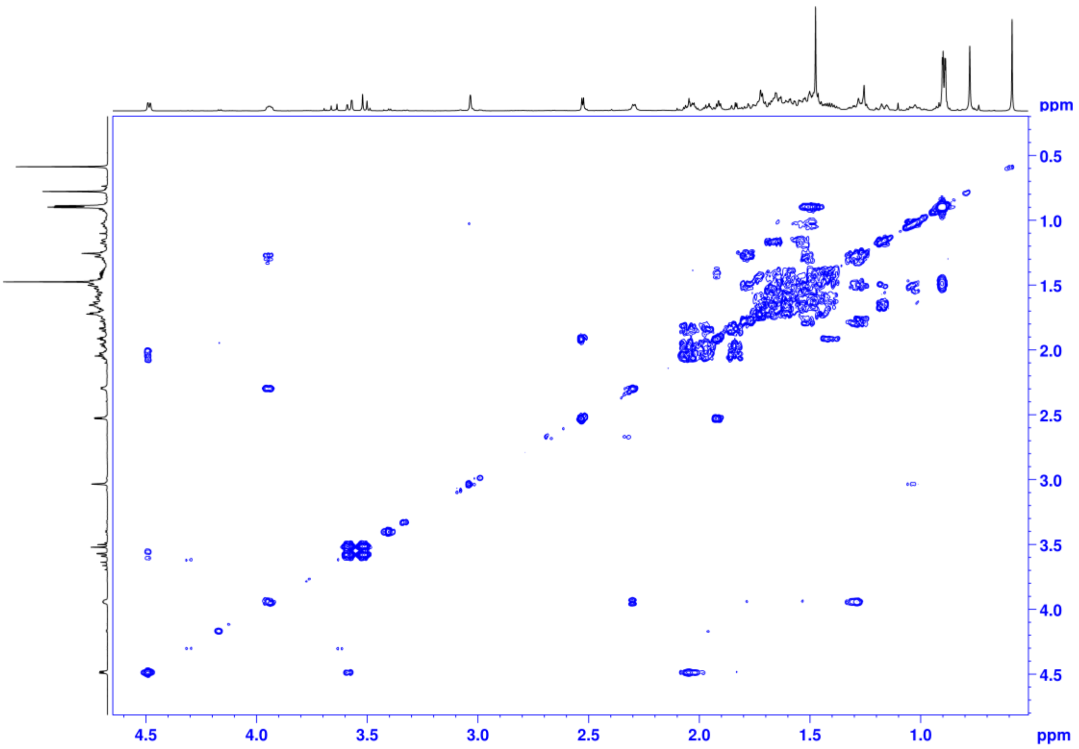

Fig. S75. ^{1}H–^{1}H COSY NMR spectrum for compound **8**

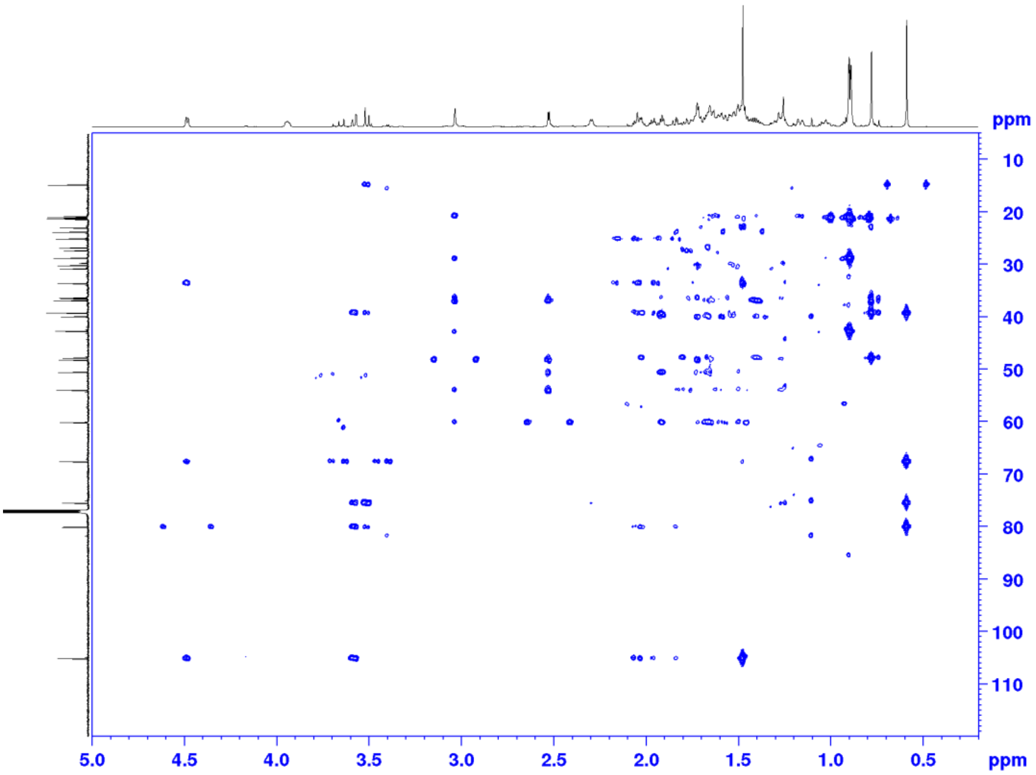

Fig. S76. HMBC NMR spectrum for compound **8**

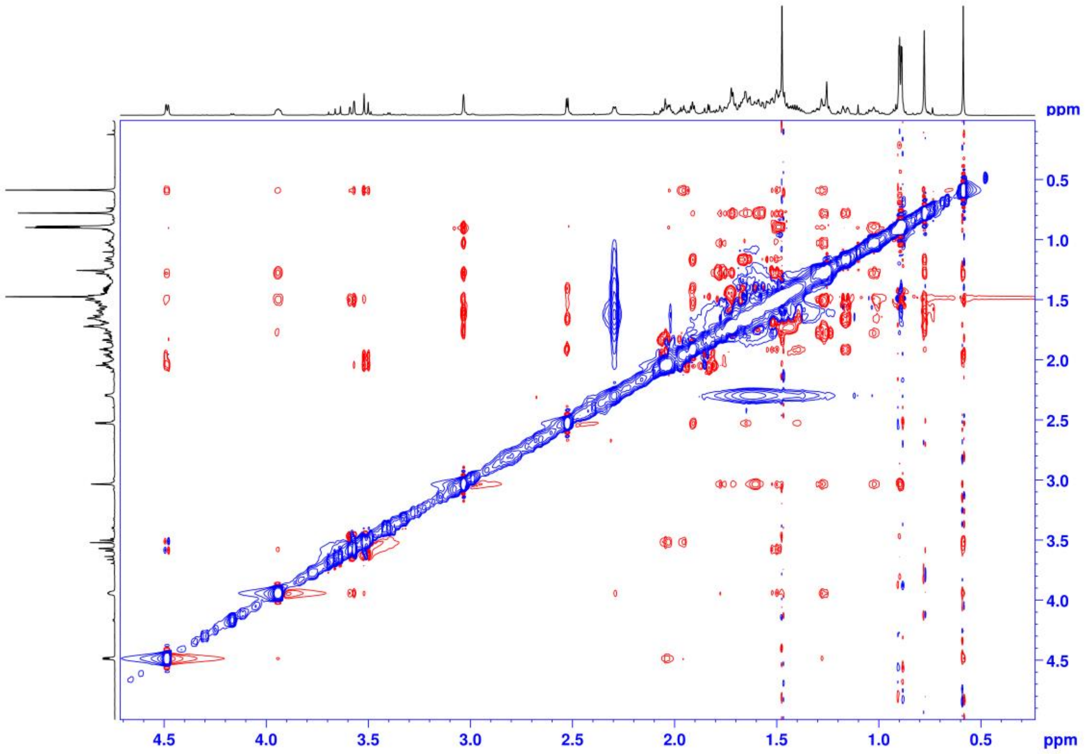

Fig. S77. NOESY NMR spectrum for compound **8**

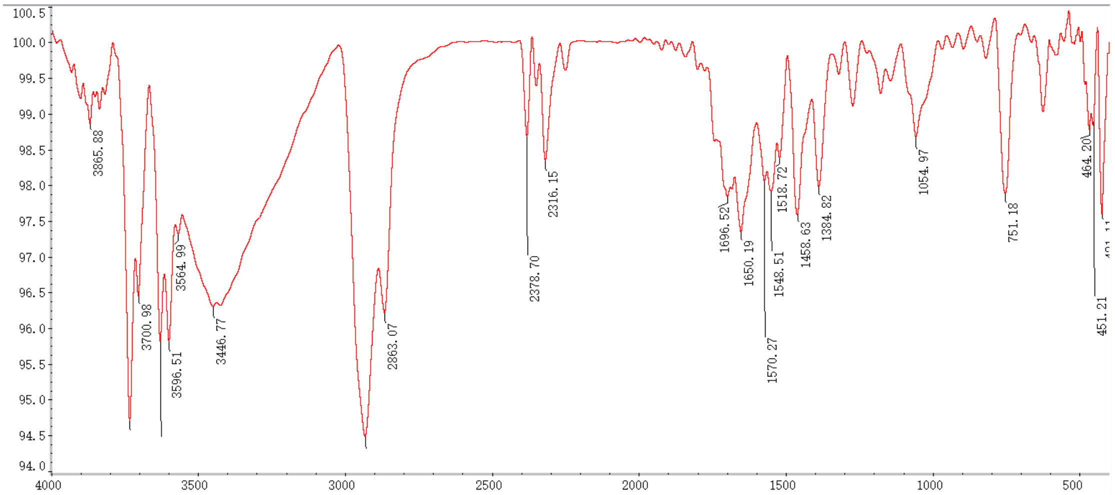

Fig. S78. IR spectrum of compound **8** (KBr)

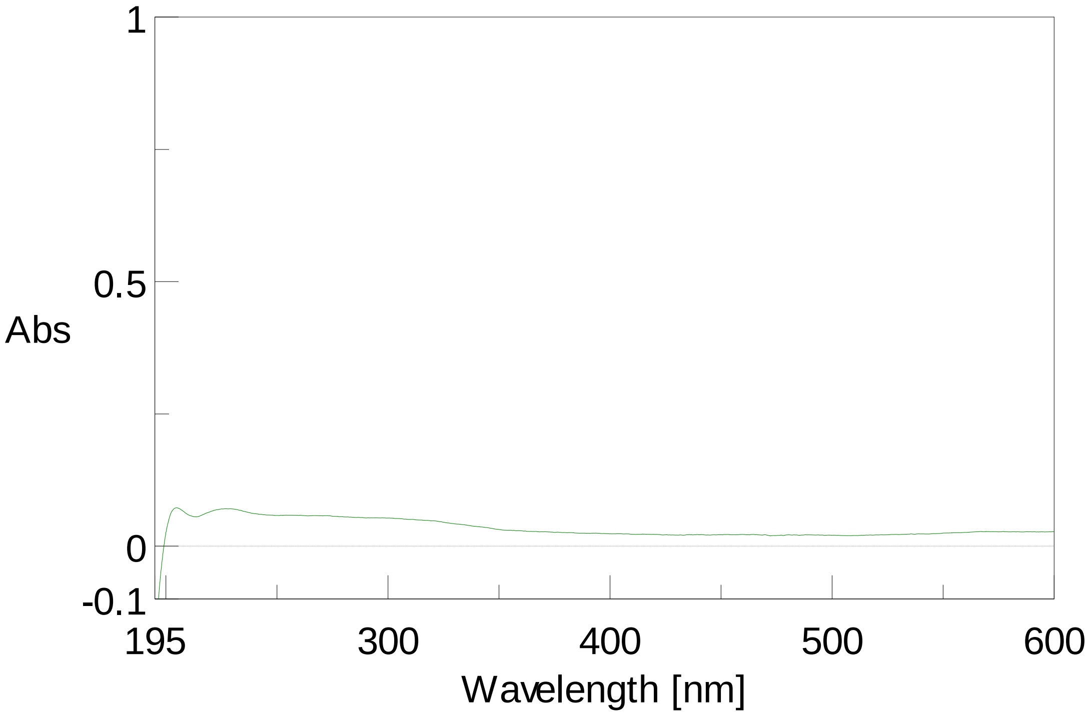

Fig. S79. UV spectrum of compound **8** in MeOH

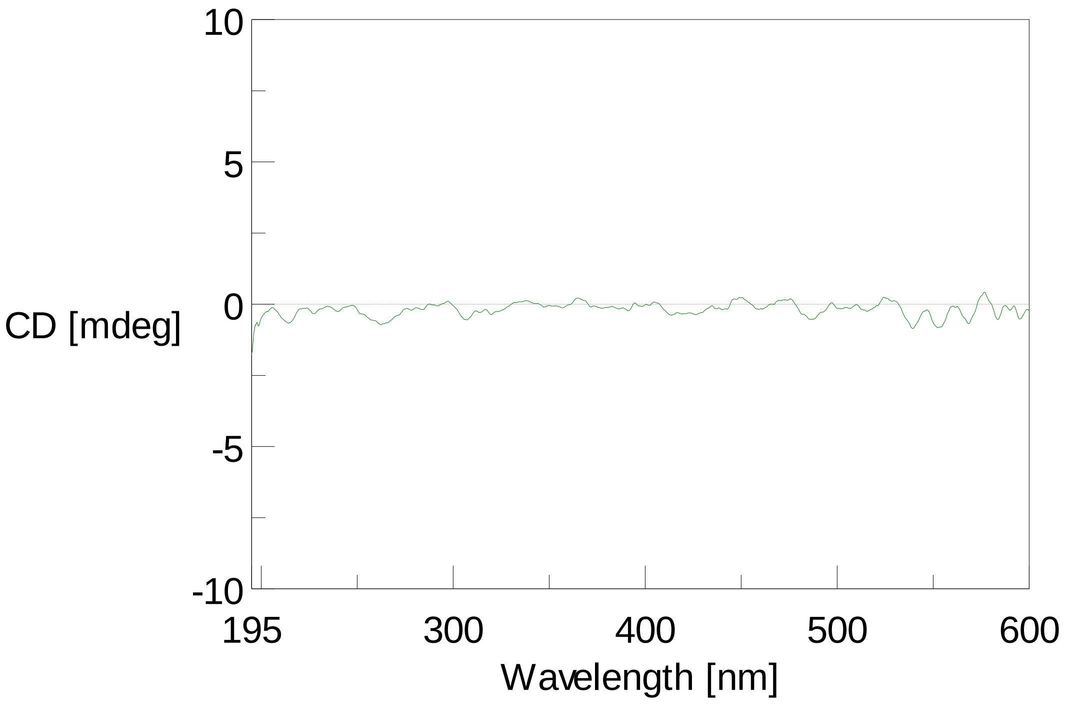

Fig. S80. CD spectrum of compound **8** in MeOH

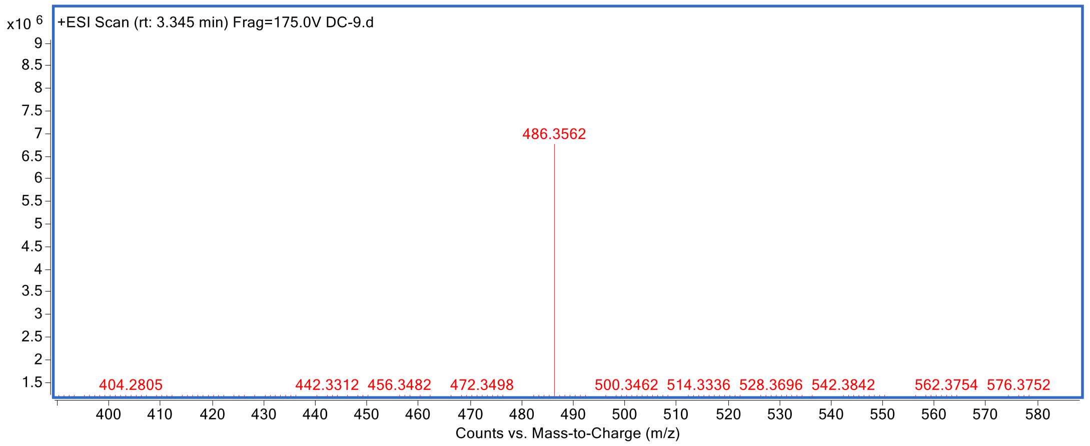

Formula (M)	Score (MFG)	Mass	Mass (MFG)	*m/z* (Calc)	Diff (ppm)	*m/z*
$C_{30}H_{47}NO_4$	94.92	485.3489	485.3505	486.3578	3.27	486.3562

Fig. S81. HR-ESI-MS spectrum of compound **9**

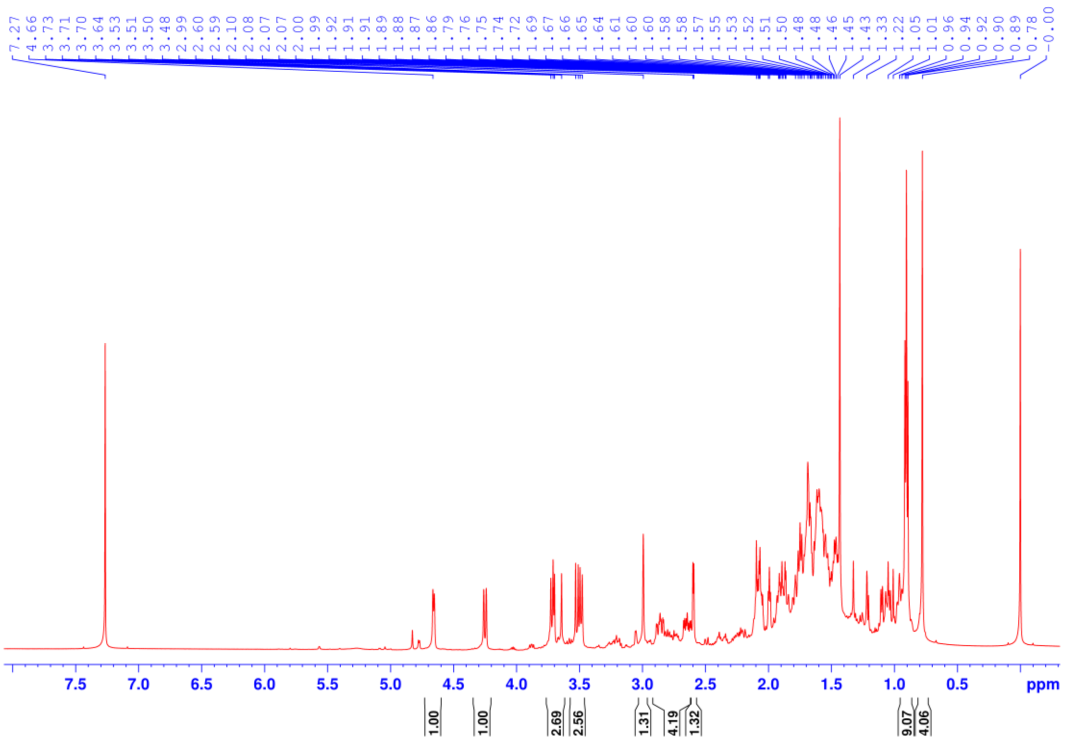

Fig. S82. ^{1}H NMR spectrum for compound **9**

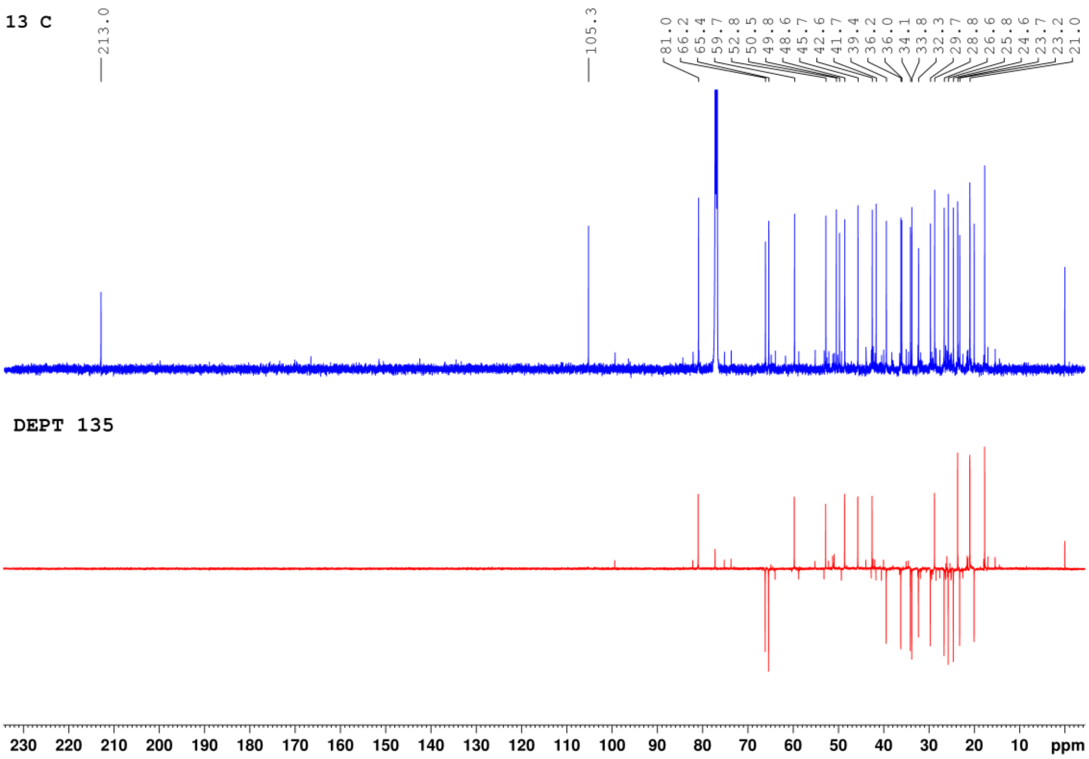

Fig. S83. ^{13}C and DEPT135 NMR spectra for compound **9**

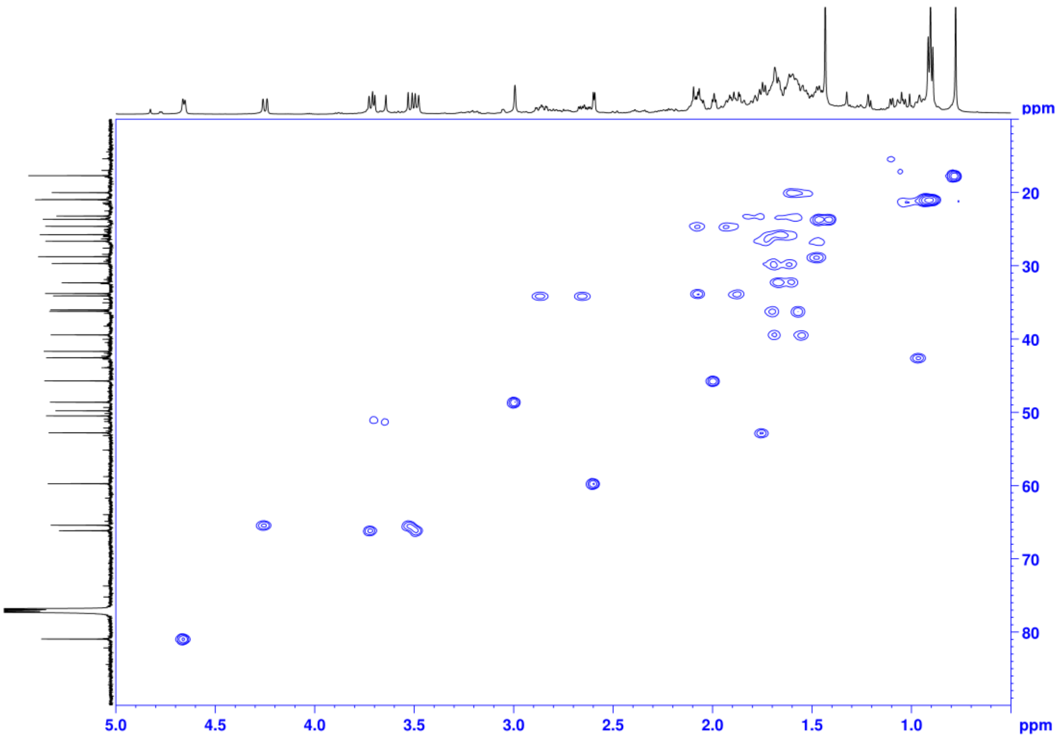

Fig. S84. HSQC NMR spectrum for compound **9**

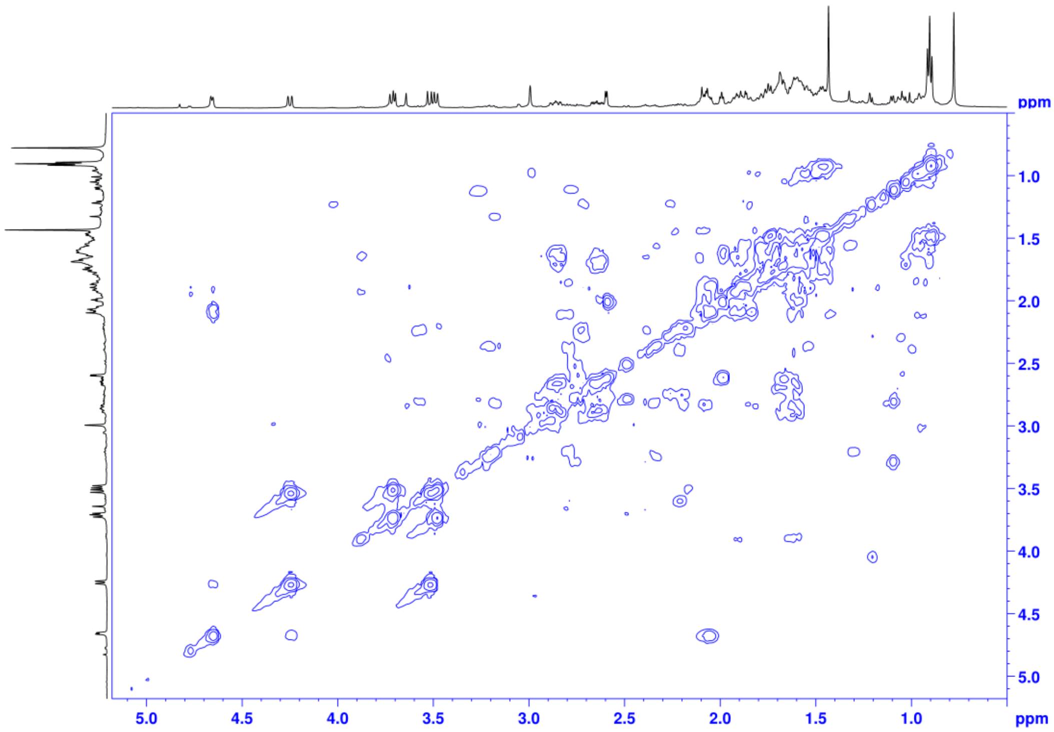

Fig. S85. $^{1}H-^{1}H$ COSY NMR spectrum for compound **9**

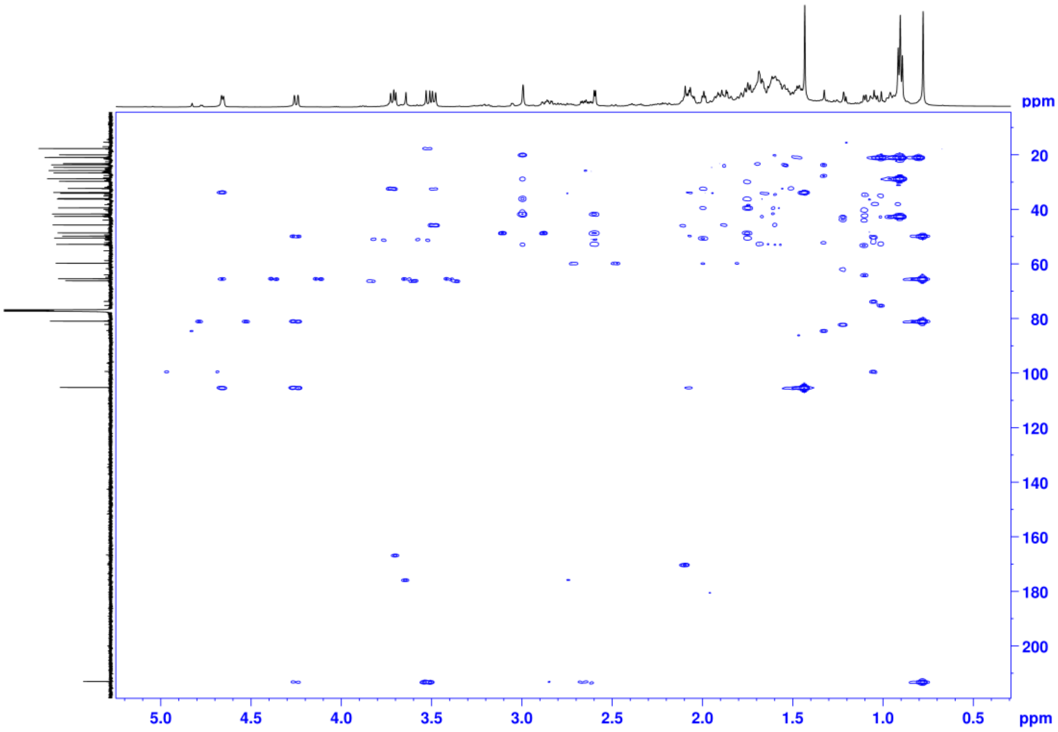

Fig. S86. HMBC NMR spectrum for compound **9**

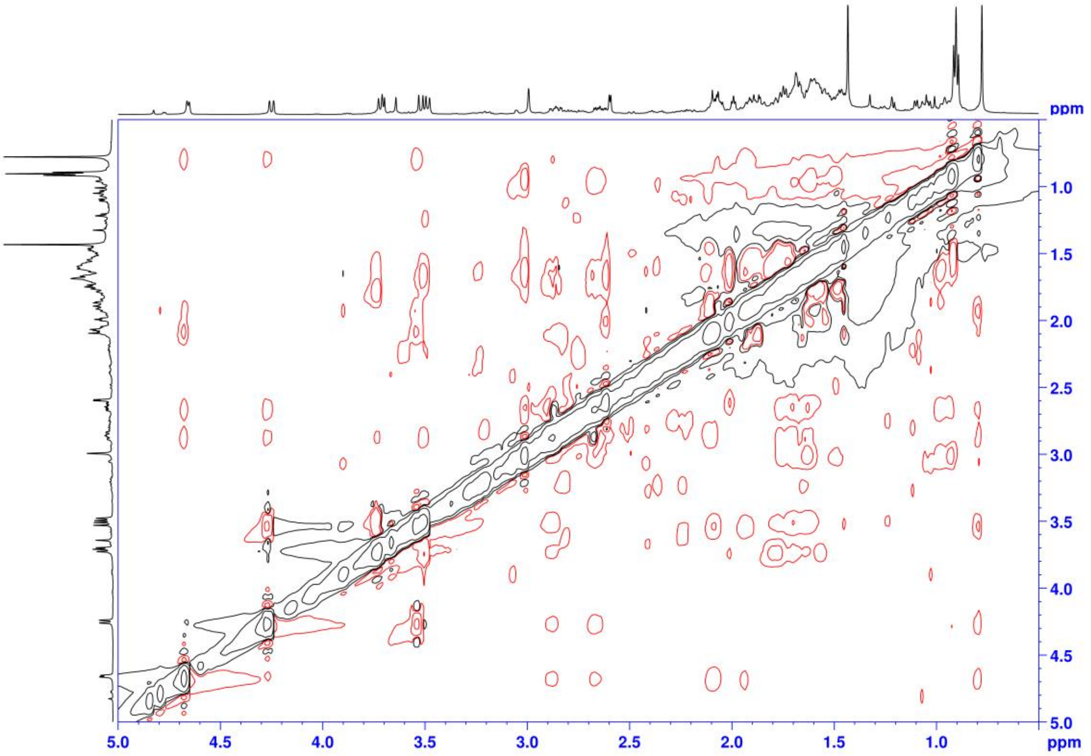

Fig. S87. NOESY NMR spectrum for compound 9

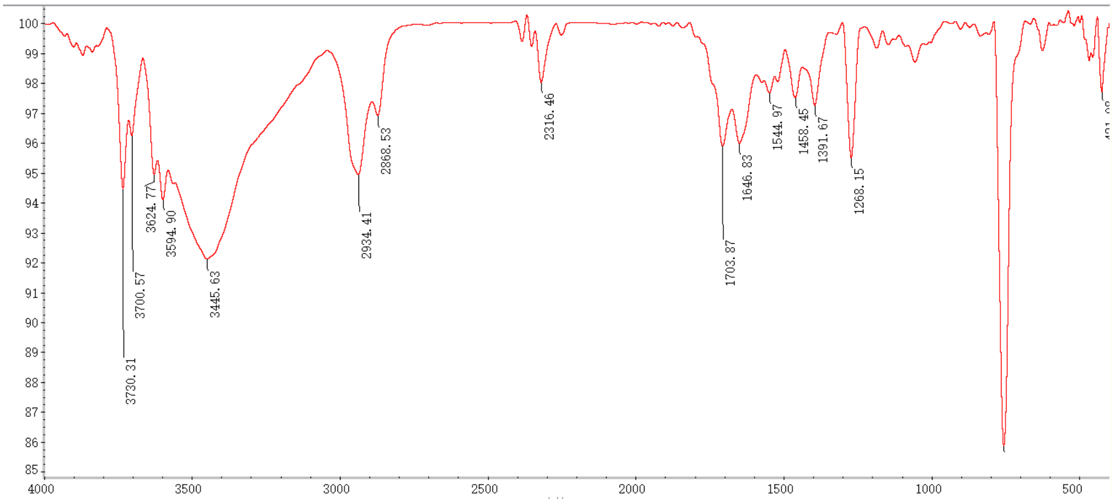

Fig. S88. IR spectrum of compound **9** (KBr)

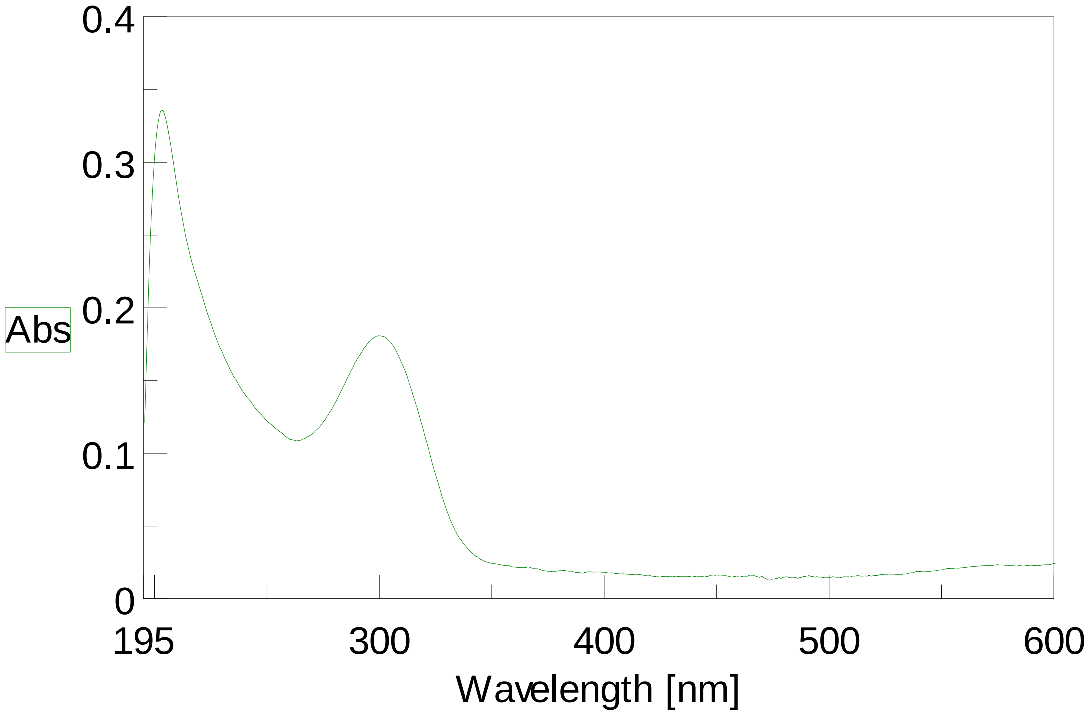

Fig. S89. UV spectrum of compound **9** in MeOH

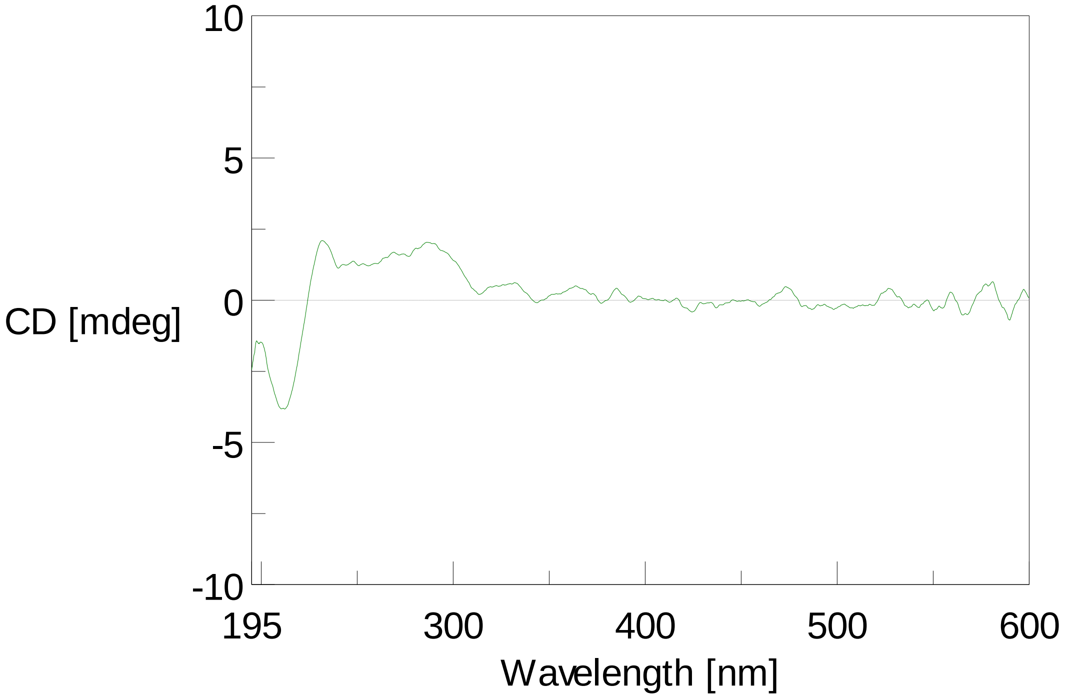

Fig. S90. CD spectrum of compound **9** in MeOH

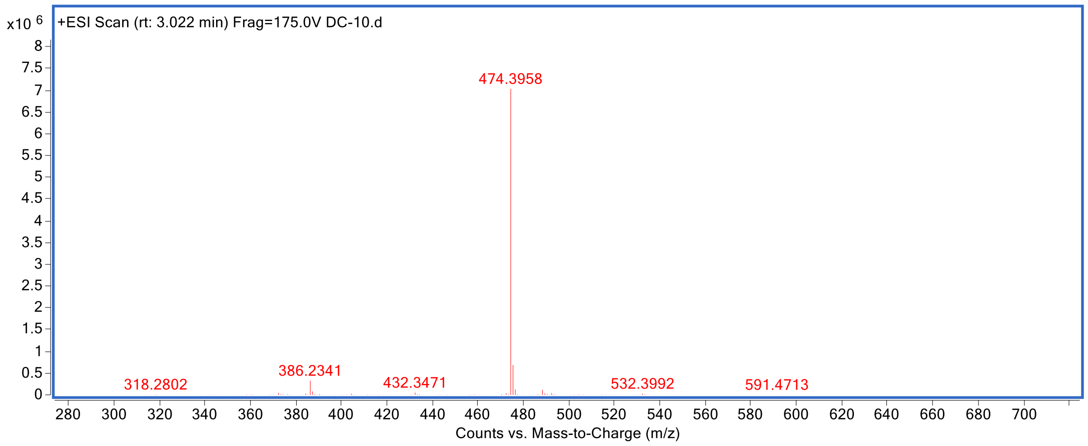

Formula (M)	Score (MFG)	Mass	Mass (MFG)	*m/z* (Calc)	Diff (ppm)	*m/z*
$C_{30}H_{51}NO_3$	94.5	473.3885	473.3869	474.3942	-3.44	474.3958

Fig. S91. HR-ESI-MS spectrum of compound **10**

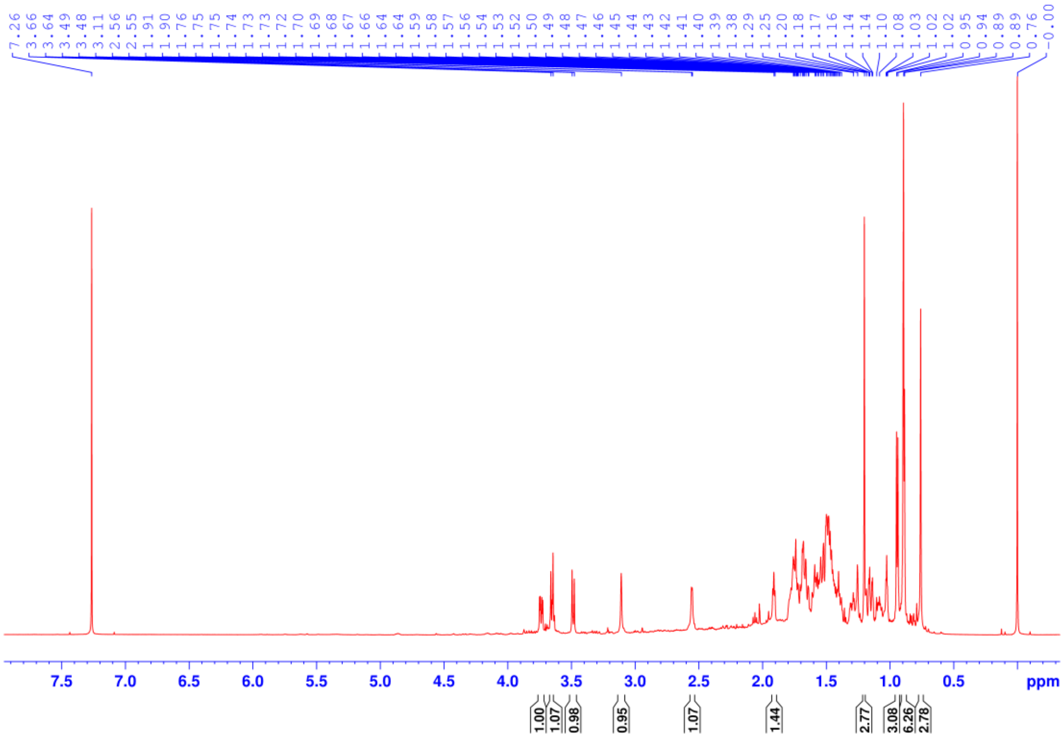

Fig. S92. ¹H NMR spectrum for compound **10**

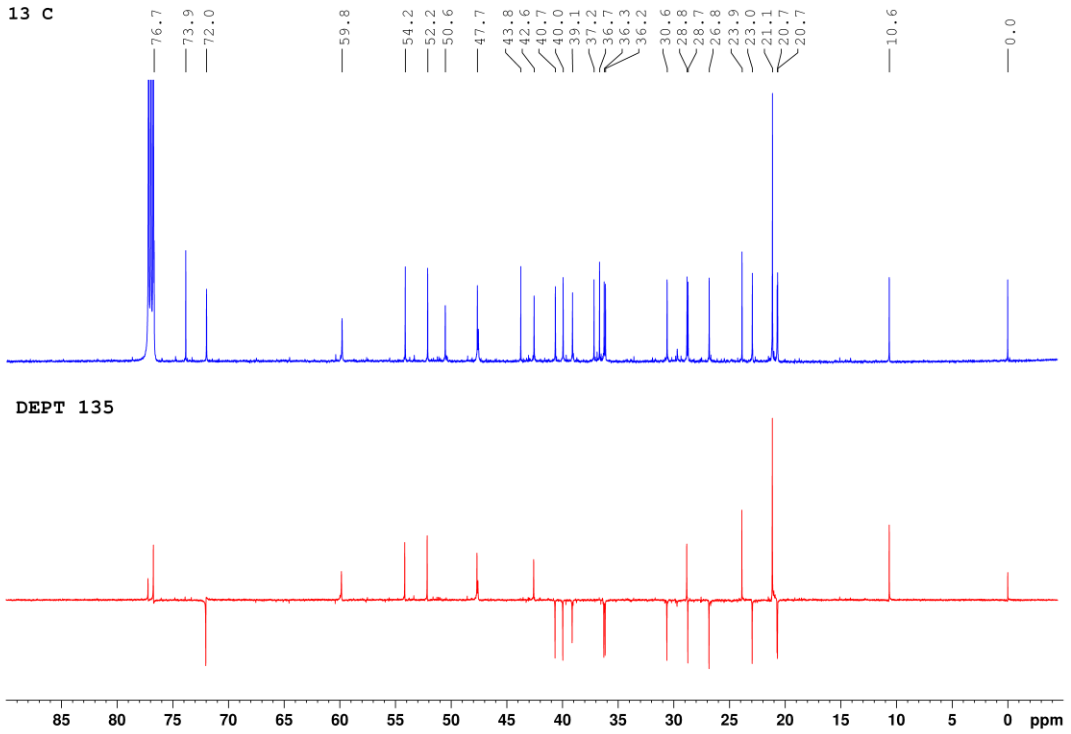

Fig. S93. ^{13}C and DEPT135 NMR spectra for compound **10**

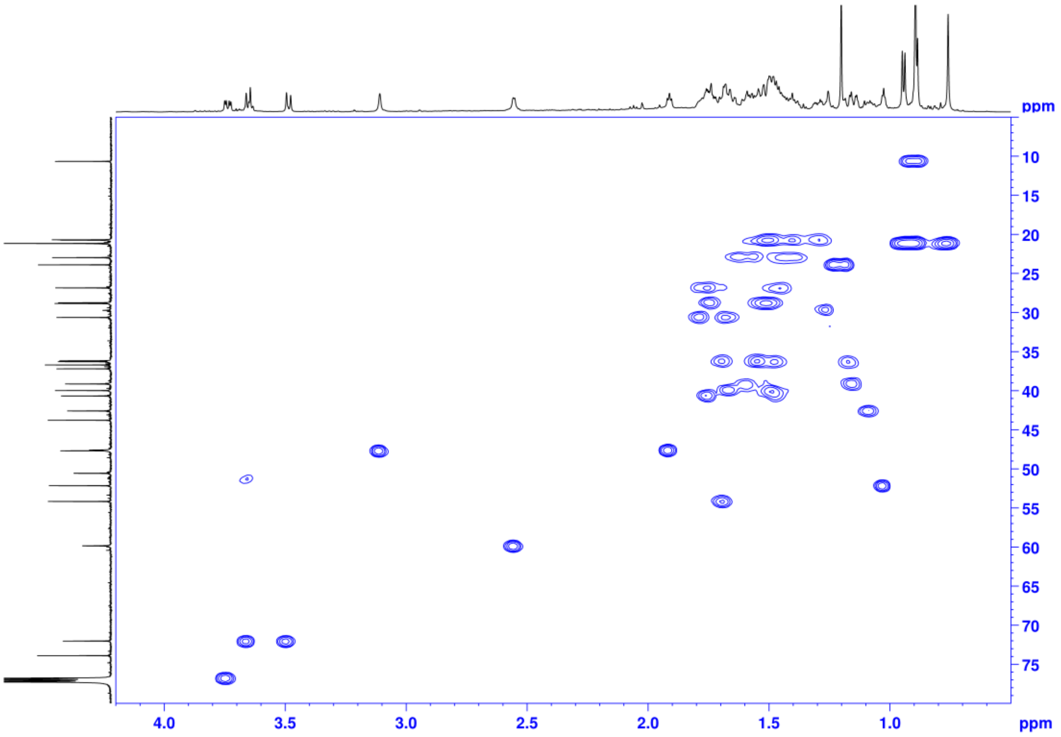

Fig. S94. HSQC NMR spectrum for compound **10**

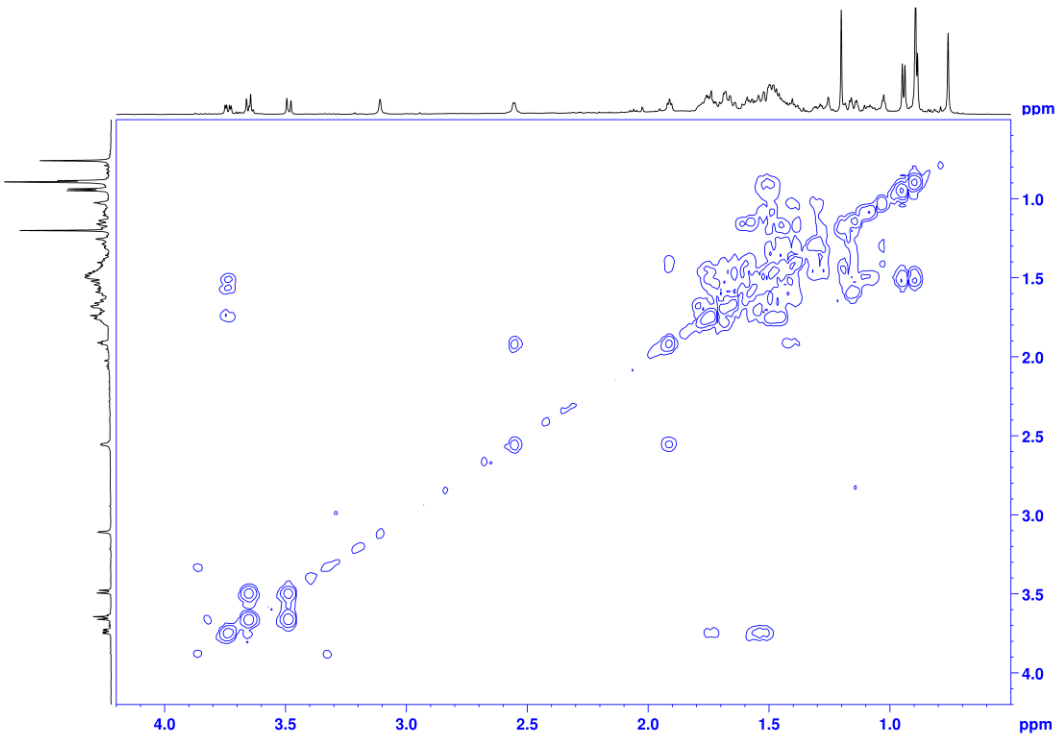

Fig. S95. ^{1}H–^{1}H COSY NMR spectrum for compound **10**

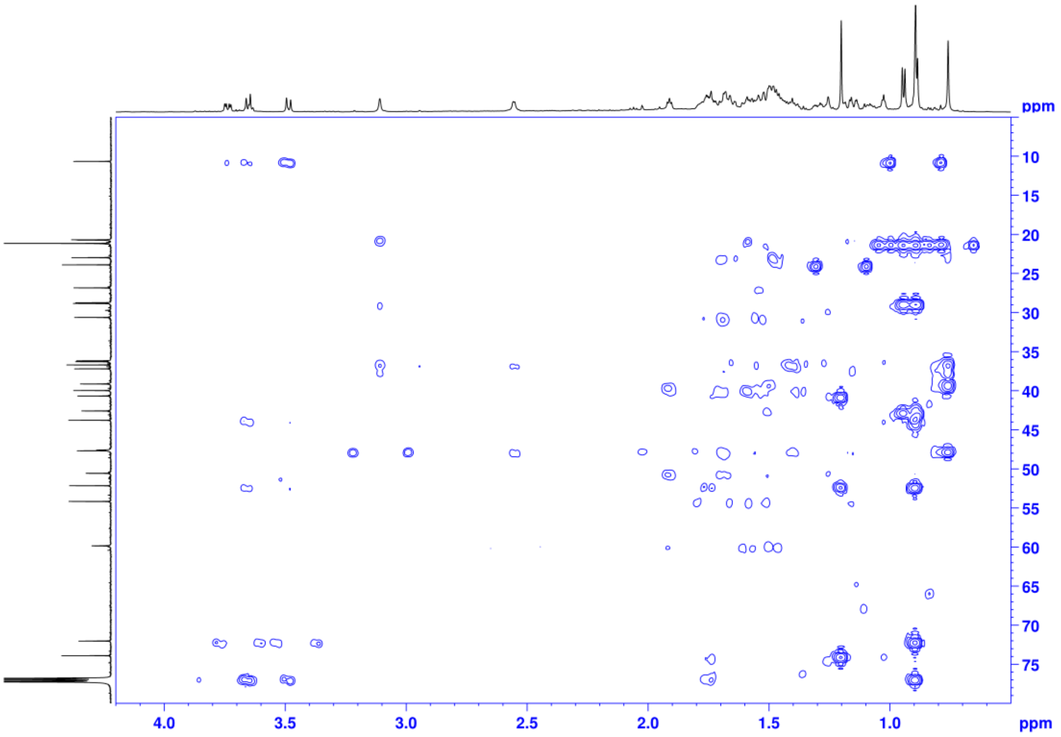

Fig. S96. HMBC NMR spectrum for compound **10**

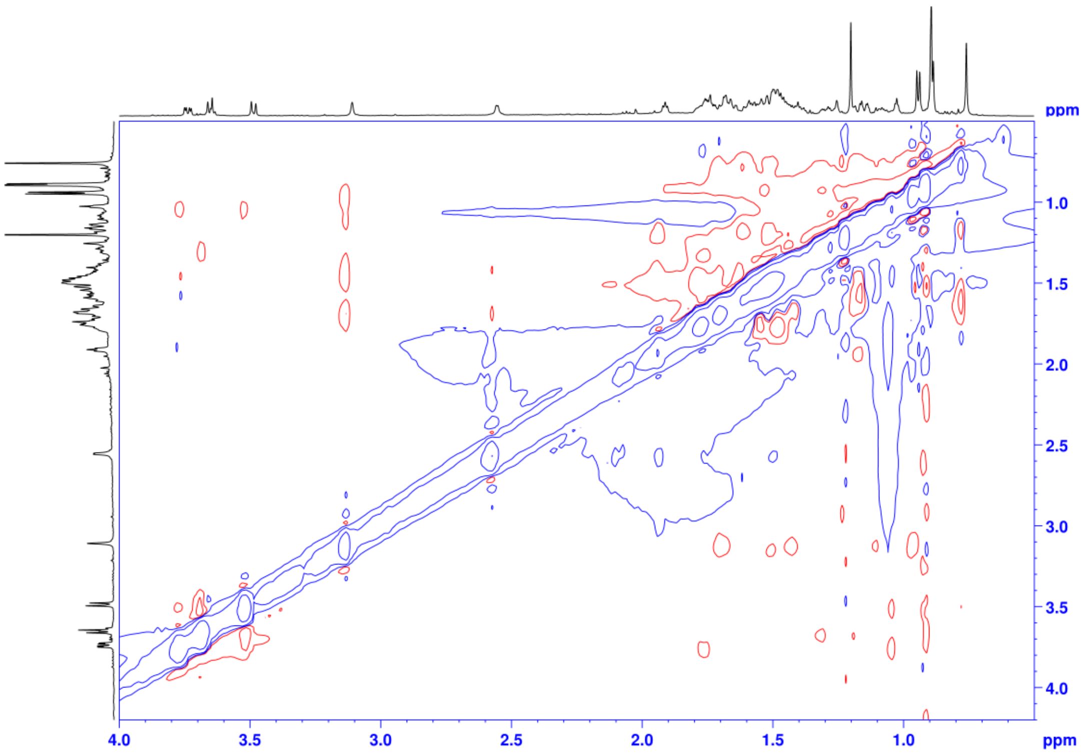

Fig. S97. NOESY NMR spectrum for compound 10

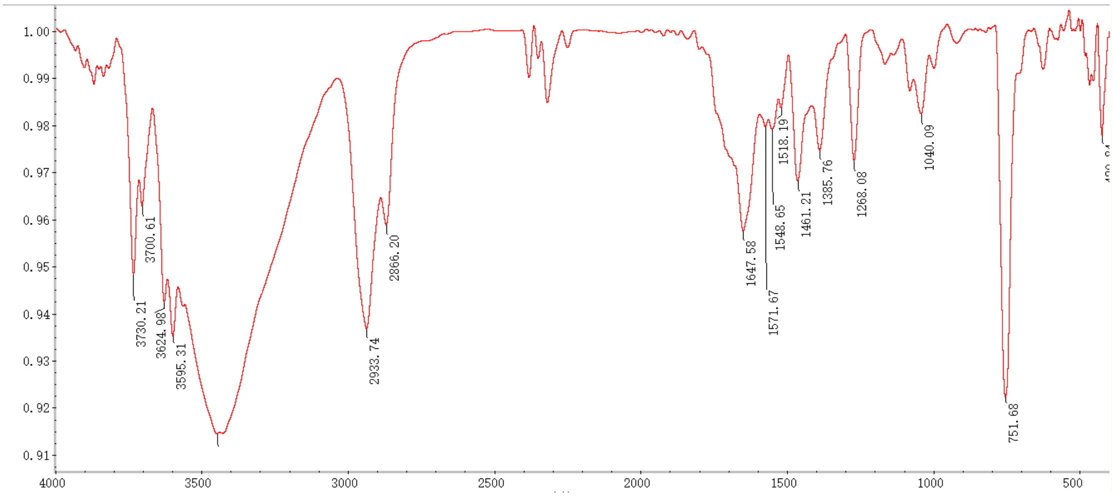

Fig. S98. IR spectrum of compound **10** (KBr)

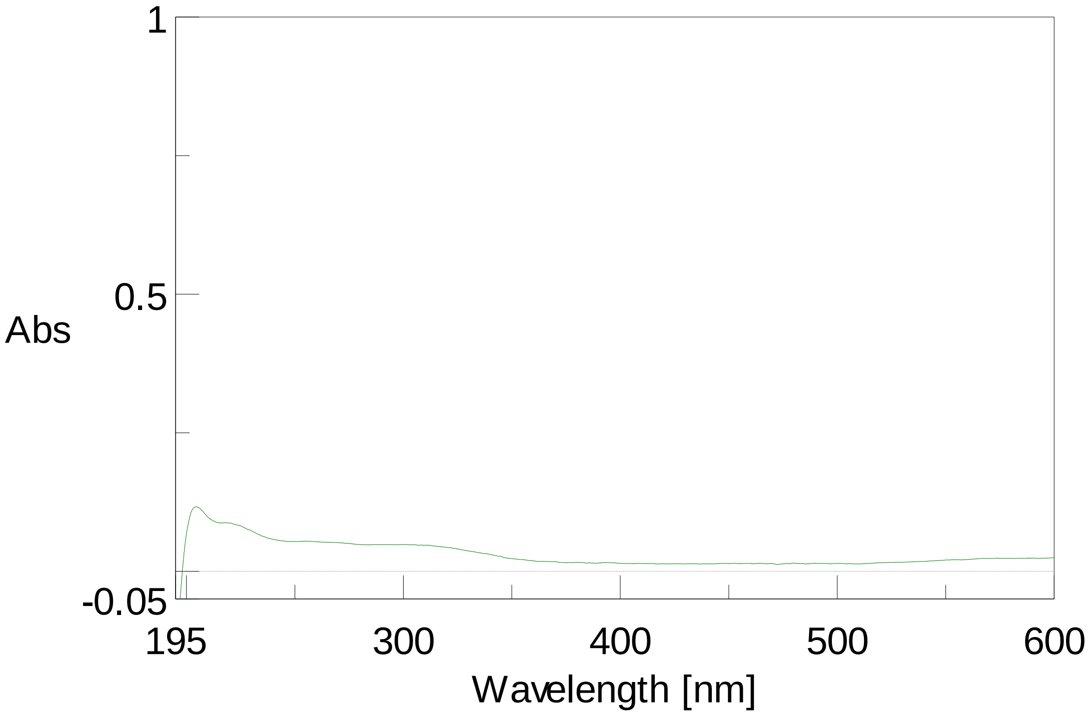

Fig. S99. UV spectrum of compound 10 in MeOH

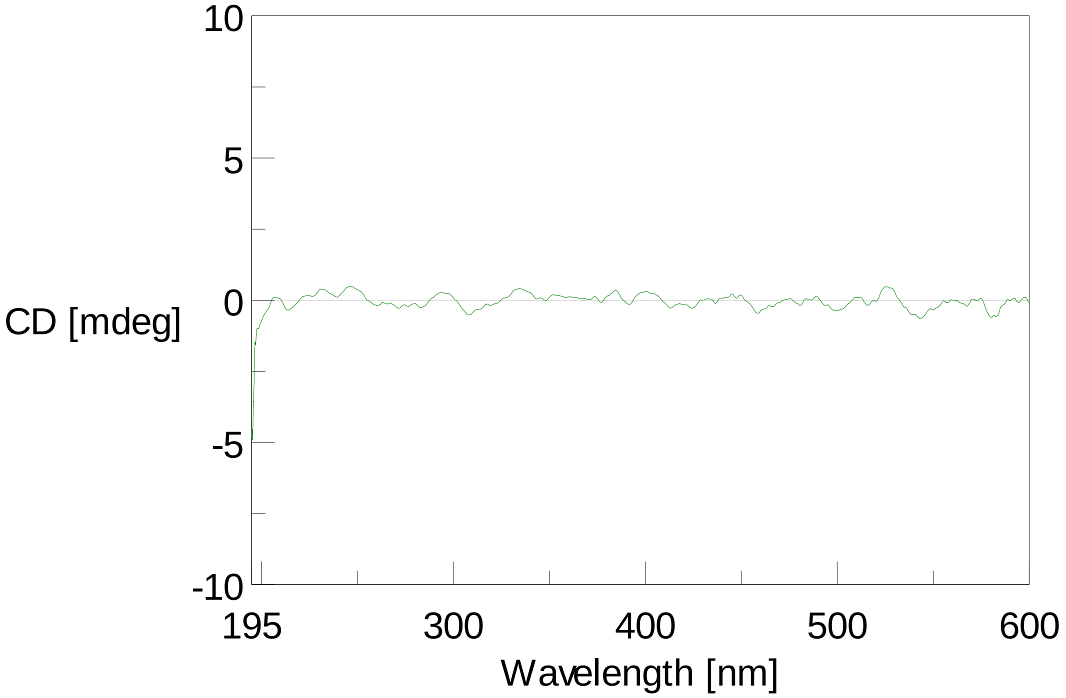

Fig. S100. CD spectrum of compound **10** in MeOH

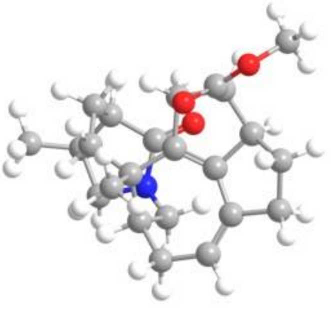

1-1 (18 %)

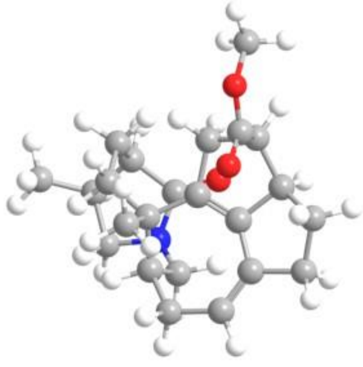

1-1 (0.37 %)

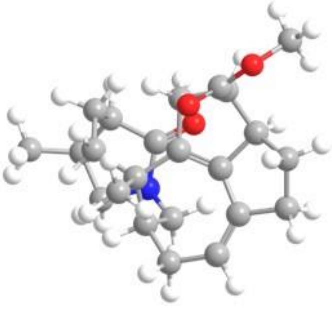

1-1 (20.25 %)

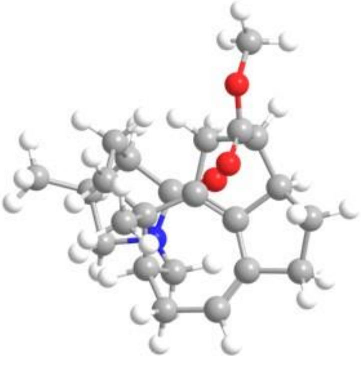

1-1 (0.39 %)

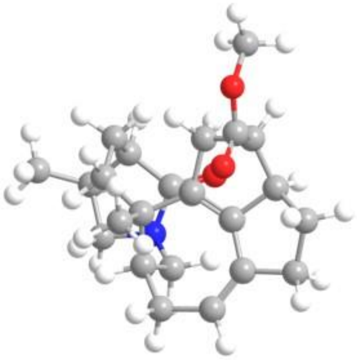

1-1 (0.41 %)

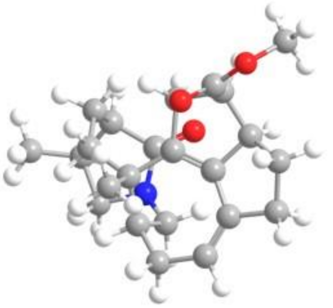

1-1 (19.6 %)

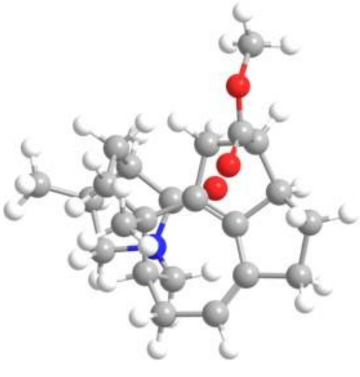

1-1 (0.38 %)

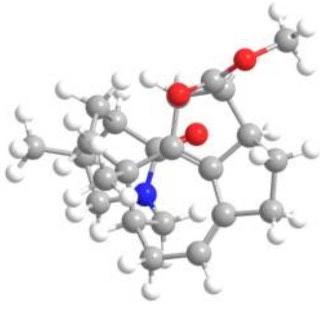

1-1 (20.5 %)

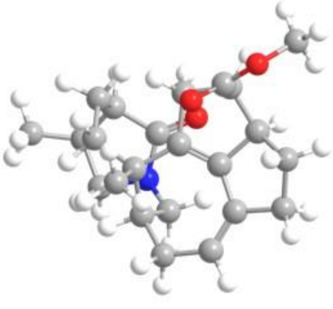

1-1 (19.3 %)

Fig. S101. Optimized conformers of compound **1**

Table S1. Gibbs free energiesa and equilibrium populationsb of low-energy conformers of **1**.

Conformers	ΔG	P(%)/100	Single point energy(a.u.)
**1-1**	8e-05	18.75	-1175.5297518288
**1-2**	0.00378	0.37	-1175.5260548182
**1-3**	1e-05	20.25	-1175.5298245595
**1-4**	0.00375	0.39	-1175.5260863631
**1-5**	0.0037	0.41	-1175.5261361246
**1-6**	4e-05	19.64	-1175.5297959616
**1-7**	0.00376	0.38	-1175.5260712545
**1-8**	0.00000	20.48	-1175.5298352839
**1-9**	5e-05	19.34	-1175.5297812377

[a]PBE0-D3(BJ)/def2-TZVP, in kcal/mol.
[b]From ΔG values at 298.15K.

Table S2. Cartesian coordinates for the low-energy reoptimized random reseach conformers of **1** at PBE0-D3(BJ)/def2-SVP level of theory in methanol.

**1-1_en**		Standard Orientation (Ångstroms)			
Center number	Atomic number	Atomic Type	X	Y	Z
0	6	0	0.480726	3.272757	1.477788
1	6	0	0.284551	0.458246	1.070574
2	6	0	2.518961	-0.616679	1.57233
3	6	0	4.531321	1.253707	2.294674
4	6	0	2.980948	3.671469	2.928386
5	6	0	3.403089	-3.161807	2.028121
6	6	0	6.028366	-2.916039	3.161694
7	6	0	6.108447	-0.213897	4.227905
8	6	0	-2.12111	-0.962914	0.563899
9	6	0	-1.629036	-3.517127	-0.873278
10	6	0	-0.453598	-5.66733	0.674356
11	6	0	2.140162	-5.333711	1.718142
12	6	0	-3.156348	-1.658417	3.209462
13	6	0	-4.318568	0.650957	-0.63223
14	6	0	-0.124469	-3.314655	-3.34487
15	7	0	-1.306558	-1.616392	-5.128966
16	6	0	-3.220419	2.283029	-5.341776
17	6	0	-0.767468	0.894145	-5.068249
18	6	0	-4.831209	0.455111	-6.901077
19	6	0	-3.849557	-2.132308	-6.068751

20	6	0	-4.087401	2.784218	-2.608284
21	8	0	1.32525	1.82022	-4.606758
22	6	0	-7.670855	0.724961	-6.710203
23	1	0	-2.989572	4.099586	-6.27727
24	1	0	-3.503718	-4.221937	-1.361479
25	1	0	5.732982	1.685772	0.667329
26	6	0	2.584012	3.868828	5.727381
27	8	0	0.78909	2.960065	6.850493
28	8	0	4.355723	5.033578	7.147402
29	6	0	6.486151	6.305061	6.015814
30	1	0	-1.137192	4.036463	2.495895
31	1	0	0.593942	4.275015	-0.318272
32	1	0	3.945974	5.348044	2.251999
33	1	0	6.430594	-4.357625	4.573612
34	1	0	7.451459	-3.106131	1.674297
35	1	0	8.026806	0.490431	4.453165
36	1	0	5.196569	-0.189641	6.077274
37	1	0	-1.731859	-6.213387	2.202779
38	1	0	-0.433884	-7.334464	-0.550108
39	1	0	3.057853	-7.06897	2.322653
40	1	0	-4.88336	-2.776597	3.046594
41	1	0	-1.786676	-2.72267	4.303889
42	1	0	-3.597628	0.042399	4.275441
43	1	0	-5.754055	-0.692058	-1.241444
44	1	0	-5.187776	1.624774	0.961596
45	1	0	1.774928	-2.630113	-2.991059
46	1	0	0.034655	-5.1859	-4.187484
47	1	0	-4.28705	0.7349	-8.87197
48	1	0	-3.772352	-3.490397	-7.615157
49	1	0	-5.035387	-2.945665	-4.600453
50	1	0	-2.882113	4.269533	-1.865278
51	1	0	-5.964615	3.632072	-2.716855
52	1	0	-8.356897	0.385189	-4.800801
53	1	0	-8.606587	-0.630833	-7.948481
54	1	0	-8.282871	2.606617	-7.281707
55	1	0	7.561545	5.054741	4.795668
56	1	0	5.866438	7.964671	4.975408
57	1	0	7.662744	6.889113	7.58861
**1-1_tddft**		Standard Orientation (Ångstroms)			
Center number	Atomic number	Atomic Type	X	Y	Z
0	6	0	0.480726	3.272757	1.477788
1	6	0	0.284551	0.458246	1.070574

2	6	0	2.518961	-0.616679	1.57233
3	6	0	4.531321	1.253707	2.294674
4	6	0	2.980948	3.671469	2.928386
5	6	0	3.403089	-3.161807	2.028121
6	6	0	6.028366	-2.916039	3.161694
7	6	0	6.108447	-0.213897	4.227905
8	6	0	-2.12111	-0.962914	0.563899
9	6	0	-1.629036	-3.517127	-0.873278
10	6	0	-0.453598	-5.66733	0.674356
11	6	0	2.140162	-5.333711	1.718142
12	6	0	-3.156348	-1.658417	3.209462
13	6	0	-4.318568	0.650957	-0.63223
14	6	0	-0.124469	-3.314655	-3.34487
15	7	0	-1.306558	-1.616392	-5.128966
16	6	0	-3.220419	2.283029	-5.341776
17	6	0	-0.767468	0.894145	-5.068249
18	6	0	-4.831209	0.455111	-6.901077
19	6	0	-3.849557	-2.132308	-6.068751
20	6	0	-4.087401	2.784218	-2.608284
21	8	0	1.32525	1.82022	-4.606758
22	6	0	-7.670855	0.724961	-6.710203
23	1	0	-2.989572	4.099586	-6.27727
24	1	0	-3.503718	-4.221937	-1.361479
25	1	0	5.732982	1.685772	0.667329
26	6	0	2.584012	3.868828	5.727381
27	8	0	0.78909	2.960065	6.850493
28	8	0	4.355723	5.033578	7.147402
29	6	0	6.486151	6.305061	6.015814
30	1	0	-1.137192	4.036463	2.495895
31	1	0	0.593942	4.275015	-0.318272
32	1	0	3.945974	5.348044	2.251999
33	1	0	6.430594	-4.357625	4.573612
34	1	0	7.451459	-3.106131	1.674297
35	1	0	8.026806	0.490431	4.453165
36	1	0	5.196569	-0.189641	6.077274
37	1	0	-1.731859	-6.213387	2.202779
38	1	0	-0.433884	-7.334464	-0.550108
39	1	0	3.057853	-7.06897	2.322653
40	1	0	-4.88336	-2.776597	3.046594
41	1	0	-1.786676	-2.72267	4.303889
42	1	0	-3.597628	0.042399	4.275441
43	1	0	-5.754055	-0.692058	-1.241444
44	1	0	-5.187776	1.624774	0.961596

45	1	0	1.774928	-2.630113	-2.991059
46	1	0	0.034655	-5.1859	-4.187484
47	1	0	-4.28705	0.7349	-8.87197
48	1	0	-3.772352	-3.490397	-7.615157
49	1	0	-5.035387	-2.945665	-4.600453
50	1	0	-2.882113	4.269533	-1.865278
51	1	0	-5.964615	3.632072	-2.716855
52	1	0	-8.356897	0.385189	-4.800801
53	1	0	-8.606587	-0.630833	-7.948481
54	1	0	-8.282871	2.606617	-7.281707
55	1	0	7.561545	5.054741	4.795668
56	1	0	5.866438	7.964671	4.975408
57	1	0	7.662744	6.889113	7.58861
**1-2_en**		Standard Orientation (Ångstroms)			
Center number	Atomic number	Atomic Type	X	Y	Z
0	6	0	1.256438	3.123307	1.24726
1	6	0	0.702509	0.330283	1.010692
2	6	0	2.791997	-0.970638	1.591975
3	6	0	4.984899	0.711299	2.240271
4	6	0	3.709002	3.234133	2.896015
5	6	0	3.419969	-3.573288	2.114095
6	6	0	6.124808	-3.583534	3.075489
7	6	0	6.580896	-0.880336	4.049489
8	6	0	-1.86868	-0.799983	0.616943
9	6	0	-1.72852	-3.497226	-0.619787
10	6	0	-0.762422	-5.656898	1.058883
11	6	0	1.907659	-5.59662	1.953681
12	6	0	-2.941024	-1.167616	3.310303
13	6	0	-3.860691	0.98955	-0.681522
14	6	0	-0.308744	-3.682297	-3.142564
15	7	0	-1.317894	-1.973307	-5.015085
16	6	0	-2.683503	2.126662	-5.490308
17	6	0	-0.440722	0.432381	-5.162041
18	6	0	-4.590152	0.430557	-6.85096
19	6	0	-3.936181	-2.204312	-5.857379
20	6	0	-3.366821	2.944631	-2.783124
21	8	0	1.770691	1.090734	-4.804042
22	6	0	-7.354488	1.114515	-6.60422
23	1	0	-2.235854	3.814678	-6.576887
24	1	0	-3.694666	-3.997245	-0.996071
25	1	0	6.109044	1.057468	0.532855
26	6	0	2.88599	3.53393	5.591208

27	8	0	2.281036	1.81037	6.992032
28	8	0	2.597571	5.899667	6.526715
29	6	0	3.204702	8.117553	5.06363
30	1	0	-0.306791	4.196745	2.051656
31	1	0	1.675792	3.969355	-0.58615
32	1	0	4.872363	4.828157	2.341292
33	1	0	6.462833	-5.029821	4.500409
34	1	0	7.40936	-3.974603	1.502649
35	1	0	8.57257	-0.363621	4.040583
36	1	0	5.882982	-0.711311	5.973349
37	1	0	-2.019804	-5.907053	2.678222
38	1	0	-1.026915	-7.402247	-0.01845
39	1	0	2.659901	-7.405717	2.568195
40	1	0	-4.799943	-2.061605	3.240746
41	1	0	-1.700456	-2.3208	4.464791
42	1	0	-3.147828	0.644182	4.261021
43	1	0	-5.448818	-0.201146	-1.225444
44	1	0	-4.61183	2.150328	0.845442
45	1	0	1.681662	-3.246678	-2.90932
46	1	0	-0.440062	-5.614812	-3.841403
47	1	0	-4.093061	0.487114	-8.853633
48	1	0	-4.08678	-3.657181	-7.310547
49	1	0	-5.168808	-2.7652	-4.308668
50	1	0	-1.916915	4.264542	-2.175478
51	1	0	-5.089725	4.073526	-2.908923
52	1	0	-7.719147	3.013242	-7.314108
53	1	0	-8.014722	1.032487	-4.657649
54	1	0	-8.518078	-0.185081	-7.701298
55	1	0	5.221059	8.214509	4.681516
56	1	0	2.131556	8.18374	3.314309
57	1	0	2.664031	9.701862	6.24699
**1-2_tddft**		Standard Orientation (Ångstroms)			
Center number	Atomic number	Atomic Type	X	Y	Z
0	6	0	1.256438	3.123307	1.24726
1	6	0	0.702509	0.330283	1.010692
2	6	0	2.791997	-0.970638	1.591975
3	6	0	4.984899	0.711299	2.240271
4	6	0	3.709002	3.234133	2.896015
5	6	0	3.419969	-3.573288	2.114095
6	6	0	6.124808	-3.583534	3.075489
7	6	0	6.580896	-0.880336	4.049489
8	6	0	-1.86868	-0.799983	0.616943

9	6	0	-1.72852	-3.497226	-0.619787
10	6	0	-0.762422	-5.656898	1.058883
11	6	0	1.907659	-5.59662	1.953681
12	6	0	-2.941024	-1.167616	3.310303
13	6	0	-3.860691	0.98955	-0.681522
14	6	0	-0.308744	-3.682297	-3.142564
15	7	0	-1.317894	-1.973307	-5.015085
16	6	0	-2.683503	2.126662	-5.490308
17	6	0	-0.440722	0.432381	-5.162041
18	6	0	-4.590152	0.430557	-6.85096
19	6	0	-3.936181	-2.204312	-5.857379
20	6	0	-3.366821	2.944631	-2.783124
21	8	0	1.770691	1.090734	-4.804042
22	6	0	-7.354488	1.114515	-6.60422
23	1	0	-2.235854	3.814678	-6.576887
24	1	0	-3.694666	-3.997245	-0.996071
25	1	0	6.109044	1.057468	0.532855
26	6	0	2.88599	3.53393	5.591208
27	8	0	2.281036	1.81037	6.992032
28	8	0	2.597571	5.899667	6.526715
29	6	0	3.204702	8.117553	5.06363
30	1	0	-0.306791	4.196745	2.051656
31	1	0	1.675792	3.969355	-0.58615
32	1	0	4.872363	4.828157	2.341292
33	1	0	6.462833	-5.029821	4.500409
34	1	0	7.40936	-3.974603	1.502649
35	1	0	8.57257	-0.363621	4.040583
36	1	0	5.882982	-0.711311	5.973349
37	1	0	-2.019804	-5.907053	2.678222
38	1	0	-1.026915	-7.402247	-0.01845
39	1	0	2.659901	-7.405717	2.568195
40	1	0	-4.799943	-2.061605	3.240746
41	1	0	-1.700456	-2.3208	4.464791
42	1	0	-3.147828	0.644182	4.261021
43	1	0	-5.448818	-0.201146	-1.225444
44	1	0	-4.61183	2.150328	0.845442
45	1	0	1.681662	-3.246678	-2.90932
46	1	0	-0.440062	-5.614812	-3.841403
47	1	0	-4.093061	0.487114	-8.853633
48	1	0	-4.08678	-3.657181	-7.310547
49	1	0	-5.168808	-2.7652	-4.308668
50	1	0	-1.916915	4.264542	-2.175478
51	1	0	-5.089725	4.073526	-2.908923

52	1	0	-7.719147	3.013242	-7.314108
53	1	0	-8.014722	1.032487	-4.657649
54	1	0	-8.518078	-0.185081	-7.701298
55	1	0	5.221059	8.214509	4.681516
56	1	0	2.131556	8.18374	3.314309
57	1	0	2.664031	9.701862	6.24699
**1-3_en**		Standard Orientation (Ångstroms)			
Center number	Atomic number	Atomic Type	X	Y	Z
0	6	0	0.439613	3.320293	1.490025
1	6	0	0.337962	0.503411	1.064381
2	6	0	2.624267	-0.489327	1.497683
3	6	0	4.580895	1.450529	2.190751
4	6	0	2.954889	3.799585	2.889188
5	6	0	3.617672	-3.002244	1.902824
6	6	0	6.260297	-2.665561	2.970569
7	6	0	6.262449	0.025357	4.066769
8	6	0	-2.023525	-1.006057	0.605495
9	6	0	-1.472446	-3.525805	-0.868668
10	6	0	-0.17449	-5.642405	0.625936
11	6	0	2.432254	-5.218558	1.602423
12	6	0	-2.961805	-1.765275	3.27006
13	6	0	-4.313799	0.530219	-0.516832
14	6	0	-0.046815	-3.245841	-3.379318
15	7	0	-1.339929	-1.576186	-5.112166
16	6	0	-3.403672	2.249545	-5.235603
17	6	0	-0.893969	0.951912	-5.038497
18	6	0	-4.983769	0.377245	-6.77367
19	6	0	-3.882726	-2.179452	-5.999037
20	6	0	-4.219466	2.689612	-2.475517
21	8	0	1.173719	1.951649	-4.619888
22	6	0	-7.825621	0.537121	-6.505039
23	1	0	-3.267162	4.083092	-6.156562
24	1	0	-3.331675	-4.29643	-1.313417
25	1	0	5.723334	1.946804	0.538879
26	6	0	2.613903	3.952917	5.698509
27	8	0	0.879316	2.967715	6.851503
28	8	0	4.372386	5.170461	7.090181
29	6	0	6.435615	6.519273	5.923538
30	1	0	-1.182985	4.020877	2.545391
31	1	0	0.480413	4.336442	-0.30153
32	1	0	3.840115	5.519744	2.212384
33	1	0	6.756048	-4.106653	4.35287

34	1	0	7.65014	-2.782278	1.444736
35	1	0	8.156771	0.802512	4.250946
36	1	0	5.397974	-0.004703	5.938689
37	1	0	-1.388033	-6.24936	2.184137
38	1	0	-0.125048	-7.297428	-0.61399
39	1	0	3.433123	-6.922378	2.162555
40	1	0	-4.64076	-2.958416	3.140568
41	1	0	-1.520936	-2.77634	4.323065
42	1	0	-3.452576	-0.09343	4.360169
43	1	0	-5.710005	-0.863927	-1.101961
44	1	0	-5.177349	1.454068	1.109397
45	1	0	1.836009	-2.495035	-3.073974
46	1	0	0.156253	-5.102286	-4.244946
47	1	0	-4.504454	0.699782	-8.754676
48	1	0	-3.788412	-3.512853	-7.565981
49	1	0	-5.002678	-3.058853	-4.517614
50	1	0	-3.05481	4.214058	-1.747177
51	1	0	-6.131271	3.462154	-2.526824
52	1	0	-8.523494	2.396507	-7.05083
53	1	0	-8.447957	0.165574	-4.579681
54	1	0	-8.743318	-0.847935	-7.72427
55	1	0	7.515218	5.317792	4.659482
56	1	0	5.741199	8.173587	4.922402
57	1	0	7.634541	7.116973	7.474229
**1-3_tddft**		Standard Orientation (Ångstroms)			
Center number	Atomic number	Atomic Type	X	Y	Z
0	6	0	0.439613	3.320293	1.490025
1	6	0	0.337962	0.503411	1.064381
2	6	0	2.624267	-0.489327	1.497683
3	6	0	4.580895	1.450529	2.190751
4	6	0	2.954889	3.799585	2.889188
5	6	0	3.617672	-3.002244	1.902824
6	6	0	6.260297	-2.665561	2.970569
7	6	0	6.262449	0.025357	4.066769
8	6	0	-2.023525	-1.006057	0.605495
9	6	0	-1.472446	-3.525805	-0.868668
10	6	0	-0.17449	-5.642405	0.625936
11	6	0	2.432254	-5.218558	1.602423
12	6	0	-2.961805	-1.765275	3.27006
13	6	0	-4.313799	0.530219	-0.516832
14	6	0	-0.046815	-3.245841	-3.379318
15	7	0	-1.339929	-1.576186	-5.112166

16	6	0	-3.403672	2.249545	-5.235603
17	6	0	-0.893969	0.951912	-5.038497
18	6	0	-4.983769	0.377245	-6.77367
19	6	0	-3.882726	-2.179452	-5.999037
20	6	0	-4.219466	2.689612	-2.475517
21	8	0	1.173719	1.951649	-4.619888
22	6	0	-7.825621	0.537121	-6.505039
23	1	0	-3.267162	4.083092	-6.156562
24	1	0	-3.331675	-4.29643	-1.313417
25	1	0	5.723334	1.946804	0.538879
26	6	0	2.613903	3.952917	5.698509
27	8	0	0.879316	2.967715	6.851503
28	8	0	4.372386	5.170461	7.090181
29	6	0	6.435615	6.519273	5.923538
30	1	0	-1.182985	4.020877	2.545391
31	1	0	0.480413	4.336442	-0.30153
32	1	0	3.840115	5.519744	2.212384
33	1	0	6.756048	-4.106653	4.35287
34	1	0	7.65014	-2.782278	1.444736
35	1	0	8.156771	0.802512	4.250946
36	1	0	5.397974	-0.004703	5.938689
37	1	0	-1.388033	-6.24936	2.184137
38	1	0	-0.125048	-7.297428	-0.61399
39	1	0	3.433123	-6.922378	2.162555
40	1	0	-4.64076	-2.958416	3.140568
41	1	0	-1.520936	-2.77634	4.323065
42	1	0	-3.452576	-0.09343	4.360169
43	1	0	-5.710005	-0.863927	-1.101961
44	1	0	-5.177349	1.454068	1.109397
45	1	0	1.836009	-2.495035	-3.073974
46	1	0	0.156253	-5.102286	-4.244946
47	1	0	-4.504454	0.699782	-8.754676
48	1	0	-3.788412	-3.512853	-7.565981
49	1	0	-5.002678	-3.058853	-4.517614
50	1	0	-3.05481	4.214058	-1.747177
51	1	0	-6.131271	3.462154	-2.526824
52	1	0	-8.523494	2.396507	-7.05083
53	1	0	-8.447957	0.165574	-4.579681
54	1	0	-8.743318	-0.847935	-7.72427
55	1	0	7.515218	5.317792	4.659482
56	1	0	5.741199	8.173587	4.922402
57	1	0	7.634541	7.116973	7.474229
**1-4_en**		Standard Orientation (Ångstroms)			

Center number	Atomic number	Atomic Type	X	Y	Z
0	6	0	1.286825	3.163802	1.301364
1	6	0	0.694255	0.375592	1.101933
2	6	0	2.774994	-0.948549	1.662774
3	6	0	5.003256	0.70776	2.260893
4	6	0	3.775684	3.261287	2.898547
5	6	0	3.370559	-3.555073	2.206328
6	6	0	6.084544	-3.593181	3.14508
7	6	0	6.58627	-0.88492	4.082653
8	6	0	-1.896355	-0.728484	0.751839
9	6	0	-1.803909	-3.432948	-0.474291
10	6	0	-0.850506	-5.594663	1.208536
11	6	0	1.827444	-5.558916	2.082501
12	6	0	-2.933306	-1.07024	3.463961
13	6	0	-3.884533	1.077215	-0.529487
14	6	0	-0.404089	-3.643573	-3.005839
15	7	0	-1.413141	-1.935317	-4.884117
16	6	0	-2.741661	2.178857	-5.360046
17	6	0	-0.51041	0.463403	-5.050044
18	6	0	-4.677767	0.496123	-6.698131
19	6	0	-4.043601	-2.139679	-5.695223
20	6	0	-3.395749	3.015492	-2.649058
21	8	0	1.712496	1.099494	-4.726602
22	6	0	-7.433891	1.207328	-6.425837
23	1	0	-2.291196	3.859924	-6.455194
24	1	0	-3.777342	-3.910768	-0.833586
25	1	0	6.117484	1.006348	0.539353
26	6	0	3.010151	3.612019	5.606502
27	8	0	2.397453	1.915901	7.038116
28	8	0	2.83259	5.99231	6.530585
29	6	0	3.464242	8.179333	5.031368
30	1	0	-0.245318	4.261125	2.13253
31	1	0	1.674019	3.985411	-0.549219
32	1	0	4.952679	4.824461	2.293079
33	1	0	6.413011	-5.025781	4.585908
34	1	0	7.349997	-4.021391	1.566788
35	1	0	8.585108	-0.397664	4.060527
36	1	0	5.895294	-0.682502	6.005875
37	1	0	-2.096353	-5.819859	2.840013
38	1	0	-1.141908	-7.340696	0.140002
39	1	0	2.557256	-7.371765	2.714716
40	1	0	-4.798068	-1.954063	3.426766

41	1	0	-1.681138	-2.220603	4.608758
42	1	0	-3.114463	0.749944	4.40338
43	1	0	-5.492472	-0.097077	-1.048102
44	1	0	-4.597848	2.254461	1.001764
45	1	0	1.588549	-3.215903	-2.784242
46	1	0	-0.55025	-5.578754	-3.691965
47	1	0	-4.197543	0.539894	-8.704447
48	1	0	-4.229799	-3.60139	-7.134439
49	1	0	-5.267379	-2.670525	-4.130116
50	1	0	-1.930245	4.326593	-2.059972
51	1	0	-5.112645	4.151974	-2.766102
52	1	0	-7.789259	3.103319	-7.148072
53	1	0	-8.066595	1.147942	-4.469637
54	1	0	-8.622584	-0.092571	-7.495438
55	1	0	2.328142	8.27493	3.324126
56	1	0	3.0278	9.789844	6.221635
57	1	0	5.465557	8.20275	4.570019
**1-4_tddft**		Standard Orientation (Ångstroms)			
Center number	Atomic number	Atomic Type	X	Y	Z
0	6	0	1.286825	3.163802	1.301364
1	6	0	0.694255	0.375592	1.101933
2	6	0	2.774994	-0.948549	1.662774
3	6	0	5.003256	0.70776	2.260893
4	6	0	3.775684	3.261287	2.898547
5	6	0	3.370559	-3.555073	2.206328
6	6	0	6.084544	-3.593181	3.14508
7	6	0	6.58627	-0.88492	4.082653
8	6	0	-1.896355	-0.728484	0.751839
9	6	0	-1.803909	-3.432948	-0.474291
10	6	0	-0.850506	-5.594663	1.208536
11	6	0	1.827444	-5.558916	2.082501
12	6	0	-2.933306	-1.07024	3.463961
13	6	0	-3.884533	1.077215	-0.529487
14	6	0	-0.404089	-3.643573	-3.005839
15	7	0	-1.413141	-1.935317	-4.884117
16	6	0	-2.741661	2.178857	-5.360046
17	6	0	-0.51041	0.463403	-5.050044
18	6	0	-4.677767	0.496123	-6.698131
19	6	0	-4.043601	-2.139679	-5.695223
20	6	0	-3.395749	3.015492	-2.649058
21	8	0	1.712496	1.099494	-4.726602
22	6	0	-7.433891	1.207328	-6.425837

23	1	0	-2.291196	3.859924	-6.455194
24	1	0	-3.777342	-3.910768	-0.833586
25	1	0	6.117484	1.006348	0.539353
26	6	0	3.010151	3.612019	5.606502
27	8	0	2.397453	1.915901	7.038116
28	8	0	2.83259	5.99231	6.530585
29	6	0	3.464242	8.179333	5.031368
30	1	0	-0.245318	4.261125	2.13253
31	1	0	1.674019	3.985411	-0.549219
32	1	0	4.952679	4.824461	2.293079
33	1	0	6.413011	-5.025781	4.585908
34	1	0	7.349997	-4.021391	1.566788
35	1	0	8.585108	-0.397664	4.060527
36	1	0	5.895294	-0.682502	6.005875
37	1	0	-2.096353	-5.819859	2.840013
38	1	0	-1.141908	-7.340696	0.140002
39	1	0	2.557256	-7.371765	2.714716
40	1	0	-4.798068	-1.954063	3.426766
41	1	0	-1.681138	-2.220603	4.608758
42	1	0	-3.114463	0.749944	4.40338
43	1	0	-5.492472	-0.097077	-1.048102
44	1	0	-4.597848	2.254461	1.001764
45	1	0	1.588549	-3.215903	-2.784242
46	1	0	-0.55025	-5.578754	-3.691965
47	1	0	-4.197543	0.539894	-8.704447
48	1	0	-4.229799	-3.60139	-7.134439
49	1	0	-5.267379	-2.670525	-4.130116
50	1	0	-1.930245	4.326593	-2.059972
51	1	0	-5.112645	4.151974	-2.766102
52	1	0	-7.789259	3.103319	-7.148072
53	1	0	-8.066595	1.147942	-4.469637
54	1	0	-8.622584	-0.092571	-7.495438
55	1	0	2.328142	8.27493	3.324126
56	1	0	3.0278	9.789844	6.221635
57	1	0	5.465557	8.20275	4.570019
**1-5_en**		Standard Orientation (Ångstroms)			
Center number	Atomic number	Atomic Type	X	Y	Z
0	6	0	1.363633	3.062116	1.414518
1	6	0	0.743642	0.288896	1.110145
2	6	0	2.795535	-1.077517	1.676182
3	6	0	5.027967	0.530987	2.379984
4	6	0	3.816409	3.07813	3.069582

5	6	0	3.348576	-3.707383	2.149031
6	6	0	6.039575	-3.808285	3.147139
7	6	0	6.547925	-1.138754	4.186008
8	6	0	-1.850152	-0.773319	0.666155
9	6	0	-1.755898	-3.438435	-0.643164
10	6	0	-0.870709	-5.663691	0.992651
11	6	0	1.786525	-5.688082	1.928504
12	6	0	-2.954806	-1.186483	3.340338
13	6	0	-3.788382	1.093549	-0.604746
14	6	0	-0.290376	-3.583883	-3.141901
15	7	0	-1.236529	-1.811824	-4.994246
16	6	0	-2.516298	2.32854	-5.372398
17	6	0	-0.308832	0.582476	-5.063163
18	6	0	-4.433663	0.707138	-6.809068
19	6	0	-3.850131	-1.963004	-5.869577
20	6	0	-3.231152	3.088253	-2.654171
21	8	0	1.911414	1.186659	-4.667321
22	6	0	-7.188985	1.437637	-6.583526
23	1	0	-2.022314	4.038274	-6.402647
24	1	0	-3.723433	-3.880506	-1.07194
25	1	0	6.185782	0.870777	0.695132
26	6	0	2.993661	3.358159	5.768543
27	8	0	2.331141	1.628886	7.137031
28	8	0	2.817566	5.713335	6.755559
29	6	0	3.503072	7.9349	5.332462
30	1	0	-0.174378	4.150459	2.246588
31	1	0	1.80534	3.940679	-0.397266
32	1	0	5.025236	4.644365	2.538731
33	1	0	6.319428	-5.291733	4.546038
34	1	0	7.335925	-4.198111	1.584085
35	1	0	8.551852	-0.674412	4.229636
36	1	0	5.810072	-0.99206	6.096856
37	1	0	-2.156221	-5.922827	2.587775
38	1	0	-1.158412	-7.372004	-0.136184
39	1	0	2.481258	-7.528295	2.519791
40	1	0	-4.82791	-2.047107	3.232376
41	1	0	-1.743697	-2.385661	4.479271
42	1	0	-3.139534	0.606361	4.330831
43	1	0	-5.398213	-0.045138	-1.192894
44	1	0	-4.522749	2.233923	0.944456
45	1	0	1.698523	-3.178425	-2.855041
46	1	0	-0.432237	-5.496316	-3.890286
47	1	0	-3.903385	0.805952	-8.800879

48	1	0	-4.0204	-3.381215	-7.353511
49	1	0	-5.114152	-2.521355	-4.346678
50	1	0	-1.771024	4.370162	-1.991342
51	1	0	-4.935971	4.242144	-2.780196
52	1	0	-7.871148	1.324751	-4.646274
53	1	0	-8.361961	0.183028	-7.722315
54	1	0	-7.50785	3.358489	-7.255274
55	1	0	2.411347	8.085122	3.600641
56	1	0	3.049125	9.516405	6.55467
57	1	0	5.515555	7.954453	4.9218
**1-5_tddft**		Standard Orientation (Ångstroms)			
Center number	Atomic number	Atomic Type	X	Y	Z
0	6	0	1.363633	3.062116	1.414518
1	6	0	0.743642	0.288896	1.110145
2	6	0	2.795535	-1.077517	1.676182
3	6	0	5.027967	0.530987	2.379984
4	6	0	3.816409	3.07813	3.069582
5	6	0	3.348576	-3.707383	2.149031
6	6	0	6.039575	-3.808285	3.147139
7	6	0	6.547925	-1.138754	4.186008
8	6	0	-1.850152	-0.773319	0.666155
9	6	0	-1.755898	-3.438435	-0.643164
10	6	0	-0.870709	-5.663691	0.992651
11	6	0	1.786525	-5.688082	1.928504
12	6	0	-2.954806	-1.186483	3.340338
13	6	0	-3.788382	1.093549	-0.604746
14	6	0	-0.290376	-3.583883	-3.141901
15	7	0	-1.236529	-1.811824	-4.994246
16	6	0	-2.516298	2.32854	-5.372398
17	6	0	-0.308832	0.582476	-5.063163
18	6	0	-4.433663	0.707138	-6.809068
19	6	0	-3.850131	-1.963004	-5.869577
20	6	0	-3.231152	3.088253	-2.654171
21	8	0	1.911414	1.186659	-4.667321
22	6	0	-7.188985	1.437637	-6.583526
23	1	0	-2.022314	4.038274	-6.402647
24	1	0	-3.723433	-3.880506	-1.07194
25	1	0	6.185782	0.870777	0.695132
26	6	0	2.993661	3.358159	5.768543
27	8	0	2.331141	1.628886	7.137031
28	8	0	2.817566	5.713335	6.755559
29	6	0	3.503072	7.9349	5.332462

30	1	0	-0.174378	4.150459	2.246588
31	1	0	1.80534	3.940679	-0.397266
32	1	0	5.025236	4.644365	2.538731
33	1	0	6.319428	-5.291733	4.546038
34	1	0	7.335925	-4.198111	1.584085
35	1	0	8.551852	-0.674412	4.229636
36	1	0	5.810072	-0.99206	6.096856
37	1	0	-2.156221	-5.922827	2.587775
38	1	0	-1.158412	-7.372004	-0.136184
39	1	0	2.481258	-7.528295	2.519791
40	1	0	-4.82791	-2.047107	3.232376
41	1	0	-1.743697	-2.385661	4.479271
42	1	0	-3.139534	0.606361	4.330831
43	1	0	-5.398213	-0.045138	-1.192894
44	1	0	-4.522749	2.233923	0.944456
45	1	0	1.698523	-3.178425	-2.855041
46	1	0	-0.432237	-5.496316	-3.890286
47	1	0	-3.903385	0.805952	-8.800879
48	1	0	-4.0204	-3.381215	-7.353511
49	1	0	-5.114152	-2.521355	-4.346678
50	1	0	-1.771024	4.370162	-1.991342
51	1	0	-4.935971	4.242144	-2.780196
52	1	0	-7.871148	1.324751	-4.646274
53	1	0	-8.361961	0.183028	-7.722315
54	1	0	-7.50785	3.358489	-7.255274
55	1	0	2.411347	8.085122	3.600641
56	1	0	3.049125	9.516405	6.55467
57	1	0	5.515555	7.954453	4.9218

**1-6_en**		Standard Orientation (Ångstroms)			
Center number	Atomic number	Atomic Type	X	Y	Z
0	6	0	0.591019	3.325337	1.378462
1	6	0	0.36511	0.496504	1.103177
2	6	0	2.579237	-0.577204	1.691196
3	6	0	4.602865	1.305685	2.348694
4	6	0	3.068329	3.761697	2.856994
5	6	0	3.431369	-3.106164	2.276811
6	6	0	6.047247	-2.835258	3.426199
7	6	0	6.151353	-0.085301	4.360002
8	6	0	-2.049266	-0.919658	0.625368
9	6	0	-1.561612	-3.547527	-0.67639
10	6	0	-0.43459	-5.632628	0.992754
11	6	0	2.149053	-5.277417	2.054105

12	6	0	-3.137428	-1.477042	3.281082
13	6	0	-4.20755	0.656289	-0.690363
14	6	0	-0.017139	-3.479408	-3.129912
15	7	0	-1.153546	-1.856106	-5.011636
16	6	0	-3.009901	2.053538	-5.444984
17	6	0	-0.58016	0.647715	-5.068205
18	6	0	-4.622849	0.170419	-6.935373
19	6	0	-3.695361	-2.382099	-5.950029
20	6	0	-3.911971	2.696804	-2.752985
21	8	0	1.519152	1.567876	-4.625951
22	6	0	-7.460678	0.491915	-6.809956
23	1	0	-2.740452	3.818041	-6.465917
24	1	0	-3.436447	-4.256123	-1.158618
25	1	0	5.819812	1.652322	0.712439
26	6	0	2.6196	4.0746	5.637815
27	8	0	0.796517	3.224466	6.761227
28	8	0	4.369761	5.287706	7.04425
29	6	0	6.535691	6.492225	5.90737
30	1	0	-1.0351	4.157727	2.326844
31	1	0	0.752721	4.238044	-0.461245
32	1	0	4.061481	5.40133	2.132341
33	1	0	6.415183	-4.209961	4.912235
34	1	0	7.483883	-3.117836	1.966515
35	1	0	8.076502	0.607572	4.561428
36	1	0	5.231794	0.038827	6.201377
37	1	0	-1.740807	-6.086942	2.526945
38	1	0	-0.418935	-7.357968	-0.148255
39	1	0	3.041342	-6.99181	2.749423
40	1	0	-4.866821	-2.594947	3.141462
41	1	0	-1.793068	-2.490306	4.452272
42	1	0	-3.591192	0.275331	4.254234
43	1	0	-5.639115	-0.700404	-1.277366
44	1	0	-5.106055	1.705319	0.838098
45	1	0	1.884004	-2.797341	-2.780332
46	1	0	0.134629	-5.390645	-3.879292
47	1	0	-4.040767	0.337814	-8.908375
48	1	0	-3.628277	-3.826305	-7.41663
49	1	0	-4.90939	-3.088123	-4.44884
50	1	0	-2.697119	4.196563	-2.056712
51	1	0	-5.773648	3.566153	-2.933244
52	1	0	-8.033718	2.343499	-7.506651
53	1	0	-8.184769	0.281138	-4.896109
54	1	0	-8.396443	-0.92373	-7.979334

55	1	0	7.647851	5.171804	4.798985
56	1	0	5.953284	8.087275	4.751231
57	1	0	7.662167	7.16889	7.479821
**1-6_tddft**		Standard Orientation (Ångstroms)			
Center number	Atomic number	Atomic Type	X	Y	Z
0	6	0	0.591019	3.325337	1.378462
1	6	0	0.36511	0.496504	1.103177
2	6	0	2.579237	-0.577204	1.691196
3	6	0	4.602865	1.305685	2.348694
4	6	0	3.068329	3.761697	2.856994
5	6	0	3.431369	-3.106164	2.276811
6	6	0	6.047247	-2.835258	3.426199
7	6	0	6.151353	-0.085301	4.360002
8	6	0	-2.049266	-0.919658	0.625368
9	6	0	-1.561612	-3.547527	-0.67639
10	6	0	-0.43459	-5.632628	0.992754
11	6	0	2.149053	-5.277417	2.054105
12	6	0	-3.137428	-1.477042	3.281082
13	6	0	-4.20755	0.656289	-0.690363
14	6	0	-0.017139	-3.479408	-3.129912
15	7	0	-1.153546	-1.856106	-5.011636
16	6	0	-3.009901	2.053538	-5.444984
17	6	0	-0.58016	0.647715	-5.068205
18	6	0	-4.622849	0.170419	-6.935373
19	6	0	-3.695361	-2.382099	-5.950029
20	6	0	-3.911971	2.696804	-2.752985
21	8	0	1.519152	1.567876	-4.625951
22	6	0	-7.460678	0.491915	-6.809956
23	1	0	-2.740452	3.818041	-6.465917
24	1	0	-3.436447	-4.256123	-1.158618
25	1	0	5.819812	1.652322	0.712439
26	6	0	2.6196	4.0746	5.637815
27	8	0	0.796517	3.224466	6.761227
28	8	0	4.369761	5.287706	7.04425
29	6	0	6.535691	6.492225	5.90737
30	1	0	-1.0351	4.157727	2.326844
31	1	0	0.752721	4.238044	-0.461245
32	1	0	4.061481	5.40133	2.132341
33	1	0	6.415183	-4.209961	4.912235
34	1	0	7.483883	-3.117836	1.966515
35	1	0	8.076502	0.607572	4.561428
36	1	0	5.231794	0.038827	6.201377

37	1	0	-1.740807	-6.086942	2.526945
38	1	0	-0.418935	-7.357968	-0.148255
39	1	0	3.041342	-6.99181	2.749423
40	1	0	-4.866821	-2.594947	3.141462
41	1	0	-1.793068	-2.490306	4.452272
42	1	0	-3.591192	0.275331	4.254234
43	1	0	-5.639115	-0.700404	-1.277366
44	1	0	-5.106055	1.705319	0.838098
45	1	0	1.884004	-2.797341	-2.780332
46	1	0	0.134629	-5.390645	-3.879292
47	1	0	-4.040767	0.337814	-8.908375
48	1	0	-3.628277	-3.826305	-7.41663
49	1	0	-4.90939	-3.088123	-4.44884
50	1	0	-2.697119	4.196563	-2.056712
51	1	0	-5.773648	3.566153	-2.933244
52	1	0	-8.033718	2.343499	-7.506651
53	1	0	-8.184769	0.281138	-4.896109
54	1	0	-8.396443	-0.92373	-7.979334
55	1	0	7.647851	5.171804	4.798985
56	1	0	5.953284	8.087275	4.751231
57	1	0	7.662167	7.16889	7.479821
**1-7_en**		Standard Orientation (Ångstroms)			
Center number	Atomic number	Atomic Type	X	Y	Z
0	6	0	1.395201	3.21451	1.28119
1	6	0	0.768245	0.429312	1.162884
2	6	0	2.830481	-0.903972	1.768163
3	6	0	5.079675	0.740663	2.318615
4	6	0	3.885214	3.328472	2.875907
5	6	0	3.388893	-3.499418	2.396709
6	6	0	6.100637	-3.545448	3.341528
7	6	0	6.640244	-0.815306	4.190864
8	6	0	-1.836632	-0.649854	0.842622
9	6	0	-1.782928	-3.395034	-0.29206
10	6	0	-0.859869	-5.51237	1.461845
11	6	0	1.818028	-5.484419	2.336385
12	6	0	-2.885736	-0.884448	3.562062
13	6	0	-3.795571	1.139372	-0.504504
14	6	0	-0.385935	-3.712283	-2.814499
15	7	0	-1.36213	-2.049046	-4.748543
16	6	0	-2.624228	2.066951	-5.364097
17	6	0	-0.421773	0.328247	-4.988461
18	6	0	-4.5829	0.371886	-6.653638

19	6	0	-3.989978	-2.241852	-5.569623
20	6	0	-3.27441	3.000967	-2.68497
21	8	0	1.809656	0.939913	-4.674824
22	6	0	-7.328818	1.131145	-6.409703
23	1	0	-2.143731	3.703928	-6.512032
24	1	0	-3.763687	-3.854813	-0.635238
25	1	0	6.199441	0.972466	0.590459
26	6	0	3.123021	3.772939	5.570538
27	8	0	2.484672	2.13035	7.052404
28	8	0	2.976309	6.182681	6.421109
29	6	0	3.640353	8.314419	4.858053
30	1	0	-0.123884	4.352431	2.081509
31	1	0	1.790074	3.979977	-0.591621
32	1	0	5.083088	4.856175	2.222837
33	1	0	6.404943	-4.933729	4.830347
34	1	0	7.363047	-4.045172	1.781905
35	1	0	8.645766	-0.357519	4.153789
36	1	0	5.952483	-0.541964	6.106437
37	1	0	-2.107953	-5.668055	3.100414
38	1	0	-1.174328	-7.288766	0.451625
39	1	0	2.522016	-7.285542	3.028468
40	1	0	-4.764436	-1.738533	3.548071
41	1	0	-1.656001	-2.016027	4.749198
42	1	0	-3.039715	0.969043	4.439358
43	1	0	-5.418217	-0.029656	-0.988378
44	1	0	-4.497388	2.377067	0.983672
45	1	0	1.613961	-3.314944	-2.603776
46	1	0	-0.568117	-5.665517	-3.437875
47	1	0	-4.098994	0.347522	-8.659318
48	1	0	-4.187032	-3.742316	-6.967036
49	1	0	-5.230984	-2.710874	-3.998856
50	1	0	-1.793984	4.310929	-2.132293
51	1	0	-4.976526	4.15444	-2.844307
52	1	0	-7.65552	3.009895	-7.188204
53	1	0	-7.967043	1.138026	-4.454309
54	1	0	-8.533524	-0.182686	-7.443776
55	1	0	2.506556	8.376124	3.147517
56	1	0	3.227226	9.966023	5.999363
57	1	0	5.64185	8.295592	4.397522
**1-7_tddft**		Standard Orientation (Ångstroms)			
Center number	Atomic number	Atomic Type	X	Y	Z
0	6	0	1.395201	3.21451	1.28119

1	6	0	0.768245	0.429312	1.162884
2	6	0	2.830481	-0.903972	1.768163
3	6	0	5.079675	0.740663	2.318615
4	6	0	3.885214	3.328472	2.875907
5	6	0	3.388893	-3.499418	2.396709
6	6	0	6.100637	-3.545448	3.341528
7	6	0	6.640244	-0.815306	4.190864
8	6	0	-1.836632	-0.649854	0.842622
9	6	0	-1.782928	-3.395034	-0.29206
10	6	0	-0.859869	-5.51237	1.461845
11	6	0	1.818028	-5.484419	2.336385
12	6	0	-2.885736	-0.884448	3.562062
13	6	0	-3.795571	1.139372	-0.504504
14	6	0	-0.385935	-3.712283	-2.814499
15	7	0	-1.36213	-2.049046	-4.748543
16	6	0	-2.624228	2.066951	-5.364097
17	6	0	-0.421773	0.328247	-4.988461
18	6	0	-4.5829	0.371886	-6.653638
19	6	0	-3.989978	-2.241852	-5.569623
20	6	0	-3.27441	3.000967	-2.68497
21	8	0	1.809656	0.939913	-4.674824
22	6	0	-7.328818	1.131145	-6.409703
23	1	0	-2.143731	3.703928	-6.512032
24	1	0	-3.763687	-3.854813	-0.635238
25	1	0	6.199441	0.972466	0.590459
26	6	0	3.123021	3.772939	5.570538
27	8	0	2.484672	2.13035	7.052404
28	8	0	2.976309	6.182681	6.421109
29	6	0	3.640353	8.314419	4.858053
30	1	0	-0.123884	4.352431	2.081509
31	1	0	1.790074	3.979977	-0.591621
32	1	0	5.083088	4.856175	2.222837
33	1	0	6.404943	-4.933729	4.830347
34	1	0	7.363047	-4.045172	1.781905
35	1	0	8.645766	-0.357519	4.153789
36	1	0	5.952483	-0.541964	6.106437
37	1	0	-2.107953	-5.668055	3.100414
38	1	0	-1.174328	-7.288766	0.451625
39	1	0	2.522016	-7.285542	3.028468
40	1	0	-4.764436	-1.738533	3.548071
41	1	0	-1.656001	-2.016027	4.749198
42	1	0	-3.039715	0.969043	4.439358
43	1	0	-5.418217	-0.029656	-0.988378

44	1	0	-4.497388	2.377067	0.983672
45	1	0	1.613961	-3.314944	-2.603776
46	1	0	-0.568117	-5.665517	-3.437875
47	1	0	-4.098994	0.347522	-8.659318
48	1	0	-4.187032	-3.742316	-6.967036
49	1	0	-5.230984	-2.710874	-3.998856
50	1	0	-1.793984	4.310929	-2.132293
51	1	0	-4.976526	4.15444	-2.844307
52	1	0	-7.65552	3.009895	-7.188204
53	1	0	-7.967043	1.138026	-4.454309
54	1	0	-8.533524	-0.182686	-7.443776
55	1	0	2.506556	8.376124	3.147517
56	1	0	3.227226	9.966023	5.999363
57	1	0	5.64185	8.295592	4.397522
**1-8_en**		Standard Orientation (Ångstroms)			
Center number	Atomic number	Atomic Type	X	Y	Z
0	6	0	0.7328	3.334989	1.149382
1	6	0	0.368644	0.510719	1.007963
2	6	0	2.511533	-0.637842	1.707825
3	6	0	4.60669	1.178871	2.329741
4	6	0	3.174249	3.719042	2.699637
5	6	0	3.230371	-3.173795	2.42381
6	6	0	5.833	-2.973965	3.617082
7	6	0	6.054068	-0.192113	4.427858
8	6	0	-2.098948	-0.80966	0.529729
9	6	0	-1.709173	-3.518169	-0.631966
10	6	0	-0.722563	-5.571535	1.160943
11	6	0	1.853397	-5.291854	2.262678
12	6	0	-3.281767	-1.18663	3.175363
13	6	0	-4.142265	0.798375	-0.922735
14	6	0	-0.110945	-3.64886	-3.049463
15	7	0	-1.114111	-2.05835	-5.032042
16	6	0	-2.752366	1.917323	-5.691651
17	6	0	-0.409691	0.407832	-5.185997
18	6	0	-4.426483	0.05114	-7.135301
19	6	0	-3.654267	-2.498037	-6.01738
20	6	0	-3.687769	2.730889	-3.057667
21	8	0	1.723544	1.238062	-4.728754
22	6	0	-7.245976	0.521578	-7.099397
23	1	0	-2.367773	3.615872	-6.785081
24	1	0	-3.606216	-4.158362	-1.124184
25	1	0	5.866224	1.400883	0.703679

26	6	0	2.640038	4.155617	5.448896
27	8	0	0.737688	3.43574	6.530791
28	8	0	4.391152	5.341804	6.877128
29	6	0	6.653762	6.399655	5.784428
30	1	0	-0.879188	4.306075	1.982033
31	1	0	1.019737	4.134277	-0.72756
32	1	0	4.263545	5.286897	1.953487
33	1	0	6.105867	-4.294764	5.171174
34	1	0	7.282492	-3.391587	2.203352
35	1	0	8.007483	0.416257	4.630961
36	1	0	5.115612	0.062941	6.246213
37	1	0	-2.079932	-5.885061	2.686086
38	1	0	-0.772249	-7.349127	0.104553
39	1	0	2.650384	-7.013914	3.048751
40	1	0	-5.057566	-2.230031	3.039015
41	1	0	-2.016908	-2.201735	4.430596
42	1	0	-3.677734	0.629885	4.051181
43	1	0	-5.609945	-0.519825	-1.507915
44	1	0	-5.047085	1.947304	0.527891
45	1	0	1.819009	-3.058757	-2.689322
46	1	0	-0.050421	-5.598907	-3.706515
47	1	0	-3.792588	0.099294	-9.098741
48	1	0	-3.622244	-4.005618	-7.420202
49	1	0	-4.939061	-3.080547	-4.521976
50	1	0	-2.414346	4.195569	-2.392754
51	1	0	-5.495364	3.686185	-3.328722
52	1	0	-8.033542	0.433123	-5.200494
53	1	0	-8.223873	-0.893975	-8.233956
54	1	0	-7.704393	2.367646	-7.889718
55	1	0	6.192738	7.971178	4.54428
56	1	0	7.74745	7.089112	7.374438
57	1	0	7.746066	4.985822	4.77637
**1-8_tddft**		Standard Orientation (Ångstroms)			
Center number	Atomic number	Atomic Type	X	Y	Z
0	6	0	0.7328	3.334989	1.149382
1	6	0	0.368644	0.510719	1.007963
2	6	0	2.511533	-0.637842	1.707825
3	6	0	4.60669	1.178871	2.329741
4	6	0	3.174249	3.719042	2.699637
5	6	0	3.230371	-3.173795	2.42381
6	6	0	5.833	-2.973965	3.617082
7	6	0	6.054068	-0.192113	4.427858

8	6	0	-2.098948	-0.80966	0.529729
9	6	0	-1.709173	-3.518169	-0.631966
10	6	0	-0.722563	-5.571535	1.160943
11	6	0	1.853397	-5.291854	2.262678
12	6	0	-3.281767	-1.18663	3.175363
13	6	0	-4.142265	0.798375	-0.922735
14	6	0	-0.110945	-3.64886	-3.049463
15	7	0	-1.114111	-2.05835	-5.032042
16	6	0	-2.752366	1.917323	-5.691651
17	6	0	-0.409691	0.407832	-5.185997
18	6	0	-4.426483	0.05114	-7.135301
19	6	0	-3.654267	-2.498037	-6.01738
20	6	0	-3.687769	2.730889	-3.057667
21	8	0	1.723544	1.238062	-4.728754
22	6	0	-7.245976	0.521578	-7.099397
23	1	0	-2.367773	3.615872	-6.785081
24	1	0	-3.606216	-4.158362	-1.124184
25	1	0	5.866224	1.400883	0.703679
26	6	0	2.640038	4.155617	5.448896
27	8	0	0.737688	3.43574	6.530791
28	8	0	4.391152	5.341804	6.877128
29	6	0	6.653762	6.399655	5.784428
30	1	0	-0.879188	4.306075	1.982033
31	1	0	1.019737	4.134277	-0.72756
32	1	0	4.263545	5.286897	1.953487
33	1	0	6.105867	-4.294764	5.171174
34	1	0	7.282492	-3.391587	2.203352
35	1	0	8.007483	0.416257	4.630961
36	1	0	5.115612	0.062941	6.246213
37	1	0	-2.079932	-5.885061	2.686086
38	1	0	-0.772249	-7.349127	0.104553
39	1	0	2.650384	-7.013914	3.048751
40	1	0	-5.057566	-2.230031	3.039015
41	1	0	-2.016908	-2.201735	4.430596
42	1	0	-3.677734	0.629885	4.051181
43	1	0	-5.609945	-0.519825	-1.507915
44	1	0	-5.047085	1.947304	0.527891
45	1	0	1.819009	-3.058757	-2.689322
46	1	0	-0.050421	-5.598907	-3.706515
47	1	0	-3.792588	0.099294	-9.098741
48	1	0	-3.622244	-4.005618	-7.420202
49	1	0	-4.939061	-3.080547	-4.521976
50	1	0	-2.414346	4.195569	-2.392754

51	1	0	-5.495364	3.686185	-3.328722
52	1	0	-8.033542	0.433123	-5.200494
53	1	0	-8.223873	-0.893975	-8.233956
54	1	0	-7.704393	2.367646	-7.889718
55	1	0	6.192738	7.971178	4.54428
56	1	0	7.74745	7.089112	7.374438
57	1	0	7.746066	4.985822	4.77637
**1-9_en**		Standard Orientation (Ångstroms)			
Center number	Atomic number	Atomic Type	X	Y	Z
0	6	0	0.716635	3.048718	1.720304
1	6	0	0.455151	0.247321	1.263386
2	6	0	2.676597	-0.881757	1.698324
3	6	0	4.741069	0.933938	2.414012
4	6	0	3.253989	3.369612	3.125567
5	6	0	3.518071	-3.452317	2.086375
6	6	0	6.170536	-3.281017	3.16905
7	6	0	6.328034	-0.602207	4.284914
8	6	0	-1.988441	-1.114912	0.778263
9	6	0	-1.57647	-3.648375	-0.718337
10	6	0	-0.415107	-5.852321	0.762761
11	6	0	2.20533	-5.59205	1.759957
12	6	0	-2.984037	-1.841045	3.430499
13	6	0	-4.175855	0.564775	-0.341409
14	6	0	-0.117245	-3.427535	-3.215816
15	7	0	-1.296091	-1.668204	-4.941345
16	6	0	-3.13478	2.271723	-5.037474
17	6	0	-0.706026	0.829284	-4.838379
18	6	0	-4.81045	0.509237	-6.603776
19	6	0	-3.865104	-2.114485	-5.845594
20	6	0	-3.940426	2.733144	-2.278106
21	8	0	1.412795	1.70404	-4.396162
22	6	0	-7.640268	0.83176	-6.354881
23	1	0	-2.884582	4.102714	-5.93919
24	1	0	-3.474107	-4.305686	-1.183522
25	1	0	5.917504	1.373212	0.770027
26	6	0	2.918141	3.522831	5.935169
27	8	0	1.128606	2.629154	7.078721
28	8	0	4.740881	4.627635	7.338442
29	6	0	6.871384	5.878936	6.184512
30	1	0	-0.864353	3.825206	2.785566
31	1	0	0.814733	4.082692	-0.058606
32	1	0	4.238574	5.03927	2.460864

33	1	0	6.573336	-4.757837	4.543913
34	1	0	7.557769	-3.469918	1.648038
35	1	0	8.264836	0.058699	4.482173
36	1	0	5.455905	-0.594924	6.153574
37	1	0	-1.673621	-6.402726	2.305926
38	1	0	-0.454403	-7.49483	-0.494165
39	1	0	3.099548	-7.357101	2.311076
40	1	0	-4.742012	-2.912317	3.283266
41	1	0	-1.618447	-2.962625	4.471843
42	1	0	-3.358525	-0.152147	4.540043
43	1	0	-5.648744	-0.737589	-0.949842
44	1	0	-4.995041	1.522114	1.288497
45	1	0	1.804823	-2.794196	-2.889789
46	1	0	-0.018435	-5.284145	-4.099234
47	1	0	-4.297358	0.820615	-8.578185
48	1	0	-3.841027	-3.43932	-7.42246
49	1	0	-5.040185	-2.93856	-4.374701
50	1	0	-2.691334	4.178927	-1.528845
51	1	0	-5.801756	3.620381	-2.332678
52	1	0	-8.300317	0.463758	-4.441476
53	1	0	-8.625586	-0.476562	-7.605664
54	1	0	-8.223258	2.737837	-6.873334
55	1	0	6.263214	7.576667	5.200125
56	1	0	8.099535	6.398136	7.740515
57	1	0	7.887546	4.636074	4.907432

**1-9_tddft**		Standard Orientation (Ångstroms)			
Center number	Atomic number	Atomic Type	X	Y	Z
0	6	0	0.716635	3.048718	1.720304
1	6	0	0.455151	0.247321	1.263386
2	6	0	2.676597	-0.881757	1.698324
3	6	0	4.741069	0.933938	2.414012
4	6	0	3.253989	3.369612	3.125567
5	6	0	3.518071	-3.452317	2.086375
6	6	0	6.170536	-3.281017	3.16905
7	6	0	6.328034	-0.602207	4.284914
8	6	0	-1.988441	-1.114912	0.778263
9	6	0	-1.57647	-3.648375	-0.718337
10	6	0	-0.415107	-5.852321	0.762761
11	6	0	2.20533	-5.59205	1.759957
12	6	0	-2.984037	-1.841045	3.430499
13	6	0	-4.175855	0.564775	-0.341409
14	6	0	-0.117245	-3.427535	-3.215816

15	7	0	-1.296091	-1.668204	-4.941345
16	6	0	-3.13478	2.271723	-5.037474
17	6	0	-0.706026	0.829284	-4.838379
18	6	0	-4.81045	0.509237	-6.603776
19	6	0	-3.865104	-2.114485	-5.845594
20	6	0	-3.940426	2.733144	-2.278106
21	8	0	1.412795	1.70404	-4.396162
22	6	0	-7.640268	0.83176	-6.354881
23	1	0	-2.884582	4.102714	-5.93919
24	1	0	-3.474107	-4.305686	-1.183522
25	1	0	5.917504	1.373212	0.770027
26	6	0	2.918141	3.522831	5.935169
27	8	0	1.128606	2.629154	7.078721
28	8	0	4.740881	4.627635	7.338442
29	6	0	6.871384	5.878936	6.184512
30	1	0	-0.864353	3.825206	2.785566
31	1	0	0.814733	4.082692	-0.058606
32	1	0	4.238574	5.03927	2.460864
33	1	0	6.573336	-4.757837	4.543913
34	1	0	7.557769	-3.469918	1.648038
35	1	0	8.264836	0.058699	4.482173
36	1	0	5.455905	-0.594924	6.153574
37	1	0	-1.673621	-6.402726	2.305926
38	1	0	-0.454403	-7.49483	-0.494165
39	1	0	3.099548	-7.357101	2.311076
40	1	0	-4.742012	-2.912317	3.283266
41	1	0	-1.618447	-2.962625	4.471843
42	1	0	-3.358525	-0.152147	4.540043
43	1	0	-5.648744	-0.737589	-0.949842
44	1	0	-4.995041	1.522114	1.288497
45	1	0	1.804823	-2.794196	-2.889789
46	1	0	-0.018435	-5.284145	-4.099234
47	1	0	-4.297358	0.820615	-8.578185
48	1	0	-3.841027	-3.43932	-7.42246
49	1	0	-5.040185	-2.93856	-4.374701
50	1	0	-2.691334	4.178927	-1.528845
51	1	0	-5.801756	3.620381	-2.332678
52	1	0	-8.300317	0.463758	-4.441476
53	1	0	-8.625586	-0.476562	-7.605664
54	1	0	-8.223258	2.737837	-6.873334
55	1	0	6.263214	7.576667	5.200125
56	1	0	8.099535	6.398136	7.740515
57	1	0	7.887546	4.636074	4.907432
